# Gas sensors based on mass-sensitive transducers. Part 2: Improving the sensors towards practical application

**DOI:** 10.1007/s00216-020-02627-3

**Published:** 2020-07-31

**Authors:** Alexandru Oprea, Udo Weimar

**Affiliations:** 1 Institute of Physical and Theoretical Chemistry, Eberhard Karls University, Tübingen, Germany; 2 Center for Light-Matter Interaction, Sensors & Analytics, Eberhard Karls University, Auf der Morgenstelle 15, 72076 Tübingen, Germany

**Keywords:** Gravimetric gas sensors, Functionalized sensing materials, Deposition methods, Targeted applications

## Abstract

Within the framework outlined in the first part of the review, the second part addresses attempts to increase receptor material performance through the use of sensor systems and chemometric methods, in conjunction with receptor preparation methods and sensor-specific tasks. Conclusions are then drawn, and development perspectives for gravimetric sensors are discussed.

## Short introduction to the second part of the review

As discussed in the introduction to the first part of the review [1], the development of gas sensors (GS) was driven by the increasing need for the detection of environmental, industrial and domestic chemical hazards. As a result of sustained investigations performed in the 1980s and 1990s, a greater understanding of gas sensing mechanisms based on mass-sensitive transducers (MST) was achieved with regard to both reception and transduction processes. However, for practical purposes, the application of this knowledge did not translate to the production of commercial devices. The main reason for this failure was the poor performance of the receptor materials, especially their low specificity. Section 2 of the second part of the review tackles this issue and presents the modalities investigated in studies aimed at increasing receptor specificity. Two main approaches are considered: increasing the specificity of the receptor–analyte interaction, and employing gas sensor systems (GSS) containing several individual devices with limited specificity in combination with chemometric methods in order to increase the overall gravimetric selectivity. The main task of either GS or GSS is the same, namely, to provide a specific and proportional response to the concentration of the analytes in the gaseous sample, ultimately enabling the determination of the sample composition or sample classification. In the third section, methods currently utilized for receptor material preparation/deposition are discussed, while the fourth section reviews attempts to improve gas detection specificity, beyond the receptor, through suitable processing of sensor arrays (SA) and GSS data. The fifth section is dedicated to the practical applications of GGSs. The first part discusses the targeted analytes, their main properties, hazards involved and legally allowed concentrations, while the second part presents a survey of applications. The conclusion focuses on the degree to which the advancements in the field of GGSs meet the expectations they have raised.

## Increasing the specificity of the receptors

**Fig. 1:**
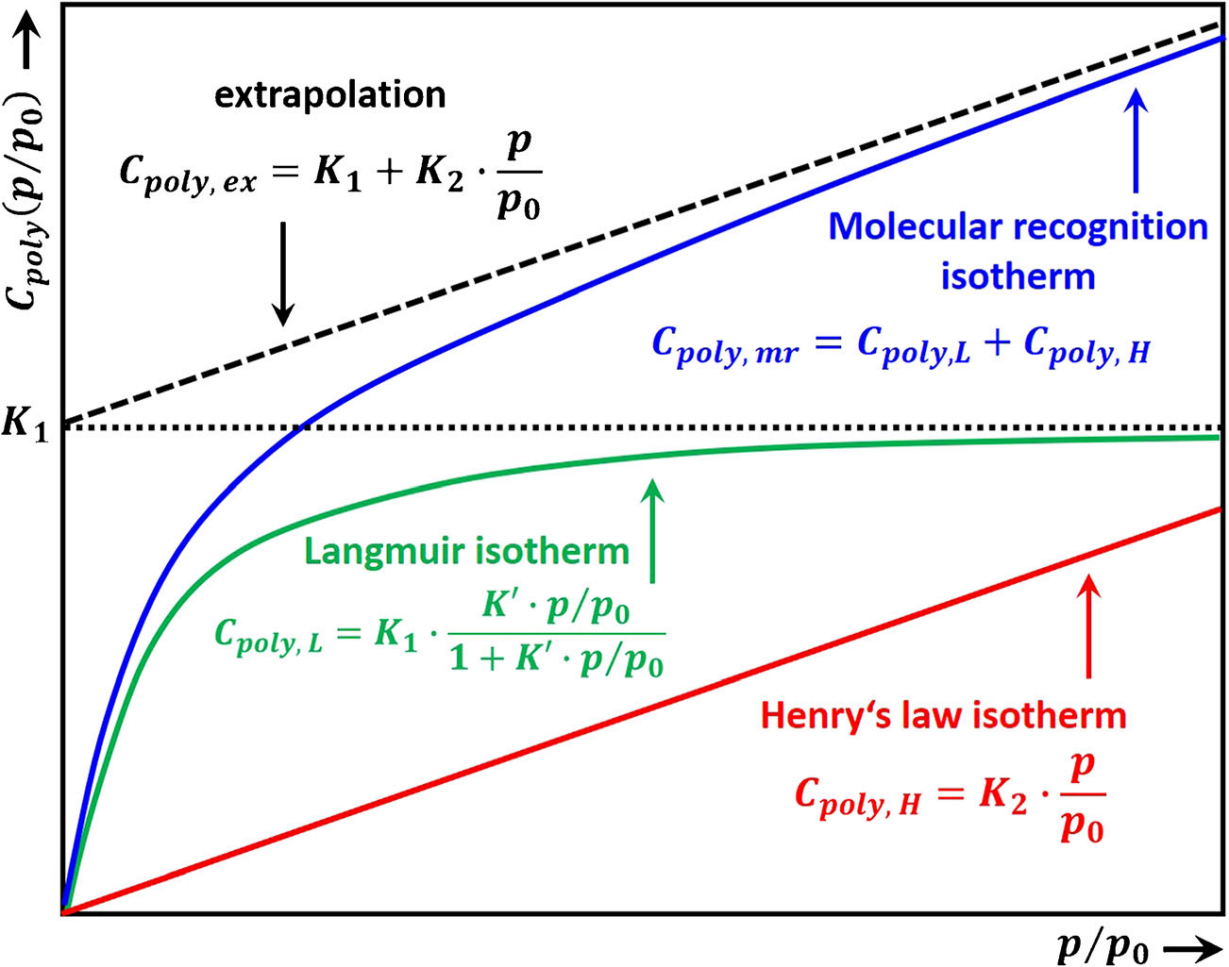
The adsorption isotherms at low analyte concentrations. Molecular recognition results in superposed Henry and Langmuir isotherms. Reproduced with the kind permission of ACS Publications from reference [2]

The interactions addressed in the section “Specific interactions and their role in receptor sensitivity and selectivity” of the first part of the review confer certain selectivity to the sensing process. However, when examining the tables with the values of solvation parameters for the specific versus nonspecific contributions (given by the product *l* ∙ *log L*^16^), it becomes obvious that in most cases, the specificity is rather low and is usually restricted to classes of analytes and not to a certain one. Because overcoming these limitations in the context of thermodynamics is very challenging, attempts have been made beyond thermodynamic-controlled specificity, searching for receptor materials whose selectivity derives from special features of the analyte–receptor interaction. In this respect, Hierlemann et al. devised combined optical and gravimetric experiments^1^ on the same receptor samples to directly probe the strength and specificity of the analyte–receptor interaction [2]. Strong correlations between changes in the infrared spectra and gravimetric sensor signals on the one hand, and the analyte type and concentration on the other hand, occurred when suitable analyte–receptor^2^ combinations were chosen. On the contrary, when analyte detection was performed with common polymers, poly(ether urethane) and poly(isobutene), the receptor response was weak and nonspecific, as expected. It is worth noting here that the laws of thermodynamics always hold, and the dispersion interaction will contribute significantly to the sensor output, regardless of how well tailored the receptor is. Therefore, in the analyte adsorption isotherm, the dispersion interaction is seen as a nonspecific contribution following Henry’s law superposed to the specific contribution (a Langmuir dependency on analyte concentration for low exposure levels) [2, 3], as depicted in Fig. 1. Moreover, the stronger the analyte–receptor interaction, the less reversible and slower the sensor.

### Molecular recognition

The specificity ensured by the complementarity of the solvation parameters, as they appear in the “linear solvation energy relationship” [4, 5] (see the first part of the review, section “Bulk receptors in the frame of the linear solvation relationship”), can be improved by mimicking the biological systems (like DNA chains) [6]. Several examples of biological binding of gases, among which that of O_2_/CO by heme molecules is the most well known, are given by Rudkevich in his review “Emerging Supramolecular Chemistry of Gases” [7]. The advances in the chemistry of the biological world led to the “molecular recognition” and “supramolecular chemistry” concepts and models. In its common meaning, molecular recognition describes the specific interaction between two molecules (one larger—the molecular receptor, and one smaller—the substrate^3^) through complementary non-covalent bonding (also included here are the specific interactions addressed in the first part of the review such as metal coordination or hydrophobic interaction). According to Lehn, “mere binding is not recognition”, so that molecular recognition is “a process involving both binding and selection of substrate(s) by a given receptor molecule” [8]. In Lehn’s view, molecular recognition involves a “double complementarity principle extending over energetical (electronic) as well as geometrical features, the celebrated ‘lock and key’, steric fit concept enunciated by Emil Fischer” in 1894 [9]. The result of molecular recognition is a supramolecular system which stores a certain amount of information (architecture of ligands, binding sites, etc.) [8]. Similarly, Cram regards molecular recognition as a guest–host interaction resulting in a guest–host complex [10]. The most relevant complementary characteristics stem from size, shape, charge, dipolar momentum and acidity-basicity of the hydrogen bond. Starting from the old “lock-and-key” picture by Fischer [9], Rebek synthesized molecular clefts for selected analytes as model receptors with high selectivity [11]. Even the formation of the U-shaped model molecule from the building blocks (2x Kemp’s triacid and naphthalene-2,7-diamine in the simplest example) was due to chemical affinity, complementarity and steric barriers. The free carboxylic groups of the cleft prepared in this way can specifically “catch” two isopropanol molecules, forming hydrogen bonds with the hydroxy groups of the alcohol. Here the appropriate size of isopropanol molecules plays an important role. However, the complementarity required for the formation of the guest–host complexes with specific receptors, leading to molecular recognition, is generally less available for the gaseous analytes because of limited dimensions, shapes and polarizability [7]. The topic of “Molecular recognition and supramolecular chemistry in the gas phase” is specifically referred to by Schalley [12].

Many natural, biological or common synthetic sensing materials employed for gas-phase detection possess certain intrinsic complementarity with respect to some target gases, so that in this case the selectivity is ensured by appropriate selection of the receptor–analyte pair through a trial procedure. In order to achieve the sensing performance required by practical applications, it is necessary to use different approaches, based on rational design and preparation (often through synthesis) of tailored receptor material for the given analytes. The natural/biological and synthetic receptors are presented in the respective sections below. The examples selected from the literature to better illustrate the receptor–analyte interaction are only roughly presented in those sections (they will mainly address the receptor material features and target analyte nature or composition). The best-performing sensors are discussed in more detail in the section dedicated to applications, pointing to the sensing parameters. Some of the experimental approaches fit more than one type of molecular recognition; in the following, a classification choice has been made, or they were considered twice.

### Natural and biological receptors

**Fig. 2:**
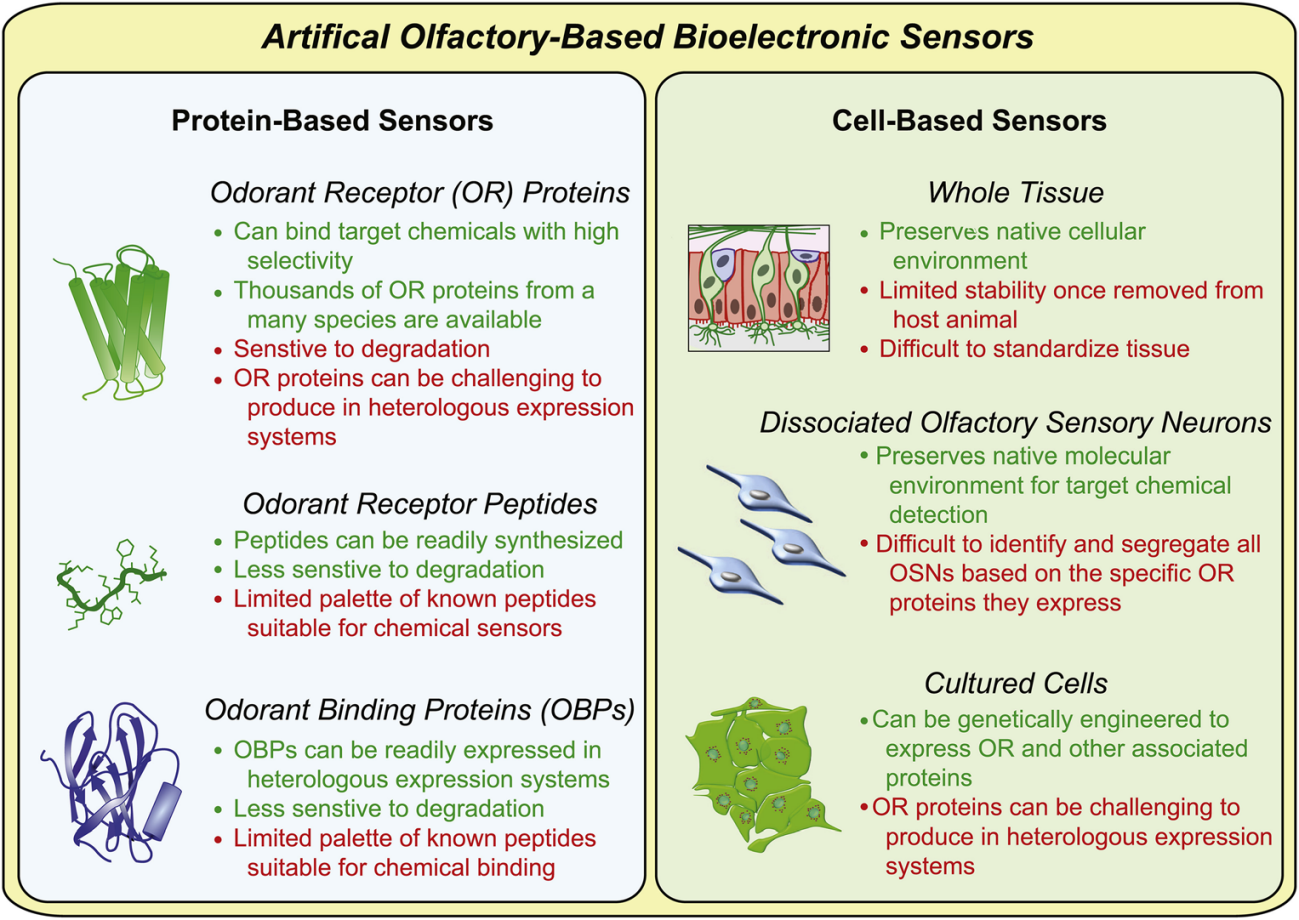
Sensors based on biological receptor materials. Advantages in green and drawbacks in red. Reproduced with the kind permission of Elsevier B.V from reference [42]

The living world abounds in good receptors for gases and vapors, integrated in sophisticated olfactory systems which are able to recognize the chemical nature of the gaseous environment. The path from reception to olfactive perception is complex and includes chemical, biochemical, physiological and, at least for humans, psychologic stages [13–16]. In the case of mammals (including humans) the olfactory process starts in the nose and ends in the brain [17]. First, the odorant is reversibly attached to the small soluble proteins—the odorant-binding proteins (OBP) [18–27]—secreted in the nasal mucus^4^. Thus, the OBPs are accessible to a large number of olfactory receptors (OR) in the nasal epithelium. The actual reception takes place when the chemical components of the odors are released from the OBPs to bind on the heptahelical protein coupled with the G-protein in the cilia of the sensitive neurons in the olfactory epithelium [31–33]. The specificity of the G-protein-coupled receptors (GPCR), which is rather limited, is encoded on the specific multigene family [34, 35]. Discrimination of the odors at the physiological level makes use of a combinatory approach [36–38]. Accordingly, one gaseous analyte is recognized by several GPCRs, but it is specifically detected only by one combination of them. Using olfactory organs based on dozens to thousands of olfactory codes (genes), animals can discern two to three orders of magnitude larger numbers of smells^5^. After probing their ability to specifically bind gases/vapors [28, 39–41], different elements of the biological odorant recognition systems, supported by adequate mathematical algorithms, have been exploited towards gas sensors, as sketched in Fig. 2 [42–46]. The simpler receptor materials (peptides, proteins, and even ORs) are more stable than the complex ones (olfactory neurons, nasal epithelium, cell cultures of olfactory tissues) and are therefore more often used in the applications. Many practical approaches employ impedimetric [39, 47, 48], field-effect [49, 50], and electrical transducers, but there are also several examples of GS based on MSTs.

Sankaran et al. used OBP LUSH peptides from *Drosophila*, self-assembled on a quartz (Q) thickness shear mode resonator (TSMR), to detect heavy alcohols (3-methyl-1-butanol and 1-hexanol) associated with *Salmonella* contamination in packaged beef. In principle, sensitivity of ~0.1Hz/ppmv with a lower limit of detection^6^ (LDL) below 5 ppmv should be enough to accomplish the task. However, no experiments were performed to identify *Salmonella* in real samples. The separation of the two alcohols addressed above using principal component analysis (PCA, see section “Evaluating the performance of the gas sensors and sensor arrays” below) was good, but humidity was not considered at all.

GGS sensor arrays consisting of gold nanoparticles modified with different peptides were employed by Compagnone et al. to detect food aromas (cis-3-hexenol, terpinen-4-ol, ethyl acetate and isopentyl acetate at 0.1% volume in ethanol, acetonitrile, acetone and hexane as solvents) [51]. Their PCA discrimination from the headspace atmosphere, created through N_2_ bubbling of solutions, was rather good. Water was excluded from the solvent list, even though the sensor array has been calibrated against humidity, because *H*_2_*O* slowed the reception mechanism.

**Fig. 3:**
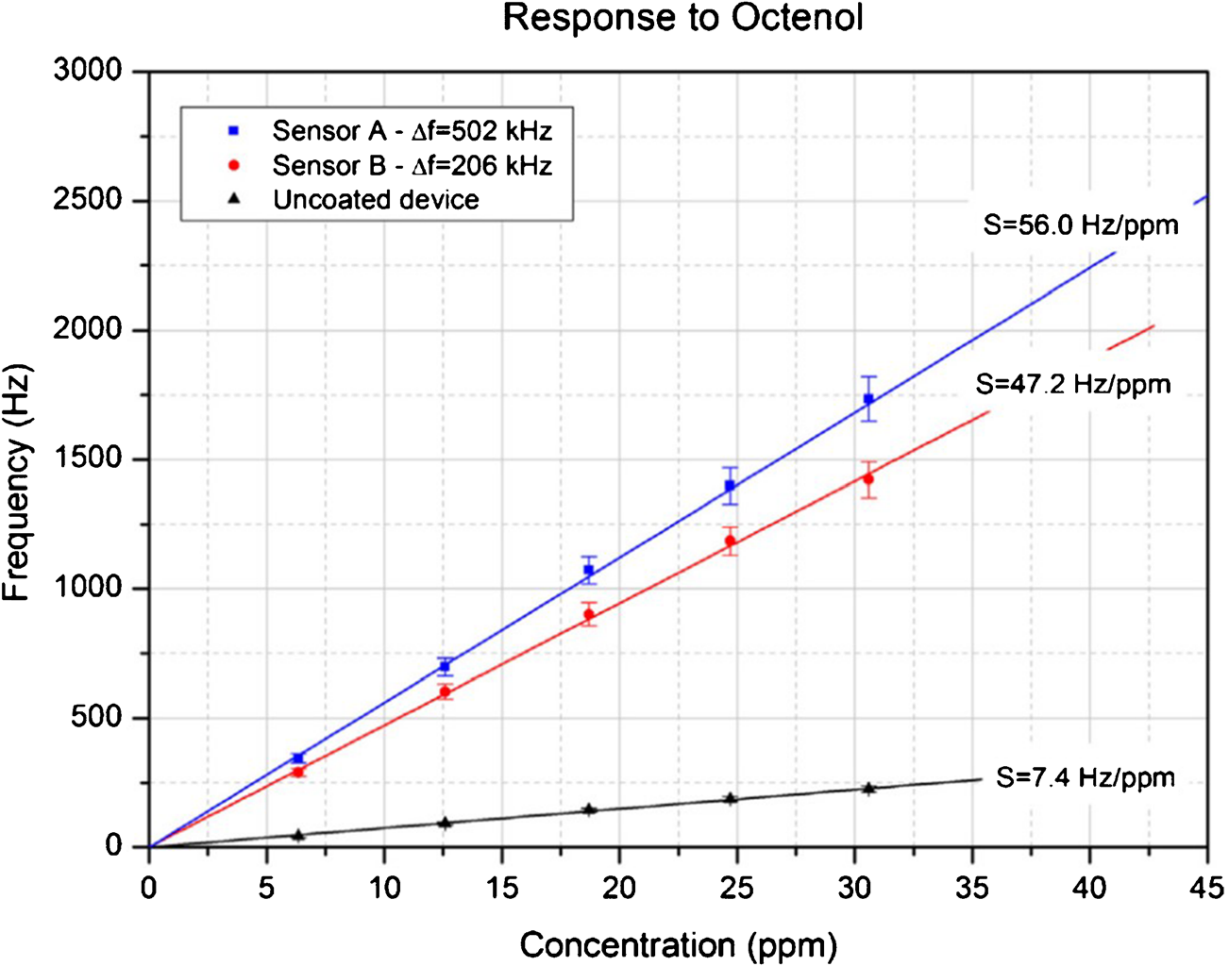
Calibration curves of three sensors coated with different OBPs (see the text for assignation) towards octenol. Reproduced with the kind permission of Elsevier B.V. from reference [52]

Sensitive detection of VOCs (octenol, carvone) with wild-type (wt) double-mutant (dm) bovine (b) and porcine (p) OBPs, respectively, was reported by Di Pietrantonio et al. [52]. The authors used two-ports 392 MHz surface acoustic wave (SAW) transducers drop-coated with OBP solutions in an array configuration (three sensing devices and an uncoated reference device). They obtained linear calibration curves (see Fig. 3) having maximal sensitivity of 25.9 Hz/ppmv and LDL of 0.39 ppmv for octenol when measured with wtpOBP-based sensors. The influence of temperature changes and humidity background was compensated by the differential readout of the sensors, each with respect to the reference SAW device. Zhao et al. proposed a thin film bulk ultra-acoustic (~1.5 GHz) resonator (FBAR) as transducer for a protein (AaegOBP22)-functionalized odorant biosensor. Saturated vapor (200 ppmv^7^) of the *N*,*N*-diethyl-3-methylbenzamide (DEET) target analyte was clearly detected, but with rather large noise [54].

Relatively recent approaches have evaluated the potential specificity and binding strength of peptides and proteins towards given gaseous analytes through molecular simulation methods [55], virtual screening [56, 57] and in silico “experiments” [58]. Synthetic poly(peptides) with the sequence “RVNEWVIC”, found to be selective for acetic acid by Wu et al. [55], were practically tested by Panigrahi et al. with GGSs based on TSMRs [59]. Sensitivity towards acetic acid of about 0.1Hz/ppmv and LDL of 2 ppmv satisfied application requirements. The humidity influence was assumed to be minimal based on chemo-physical justifications, but no dedicated measurements were carried out. The achievements based on virtual screening and in silico approaches will be addressed below in the section “Gravimetric sensor systems”, as they better fit this topic.

### Synthetic/tailored receptors

Because the long-term stability of the biological receptors is rather poor, many researchers have replaced them with synthetic materials having engineered sensing properties. The most successful classes are discussed below.

#### Molecularly imprinted polymers

**Fig. 4:**
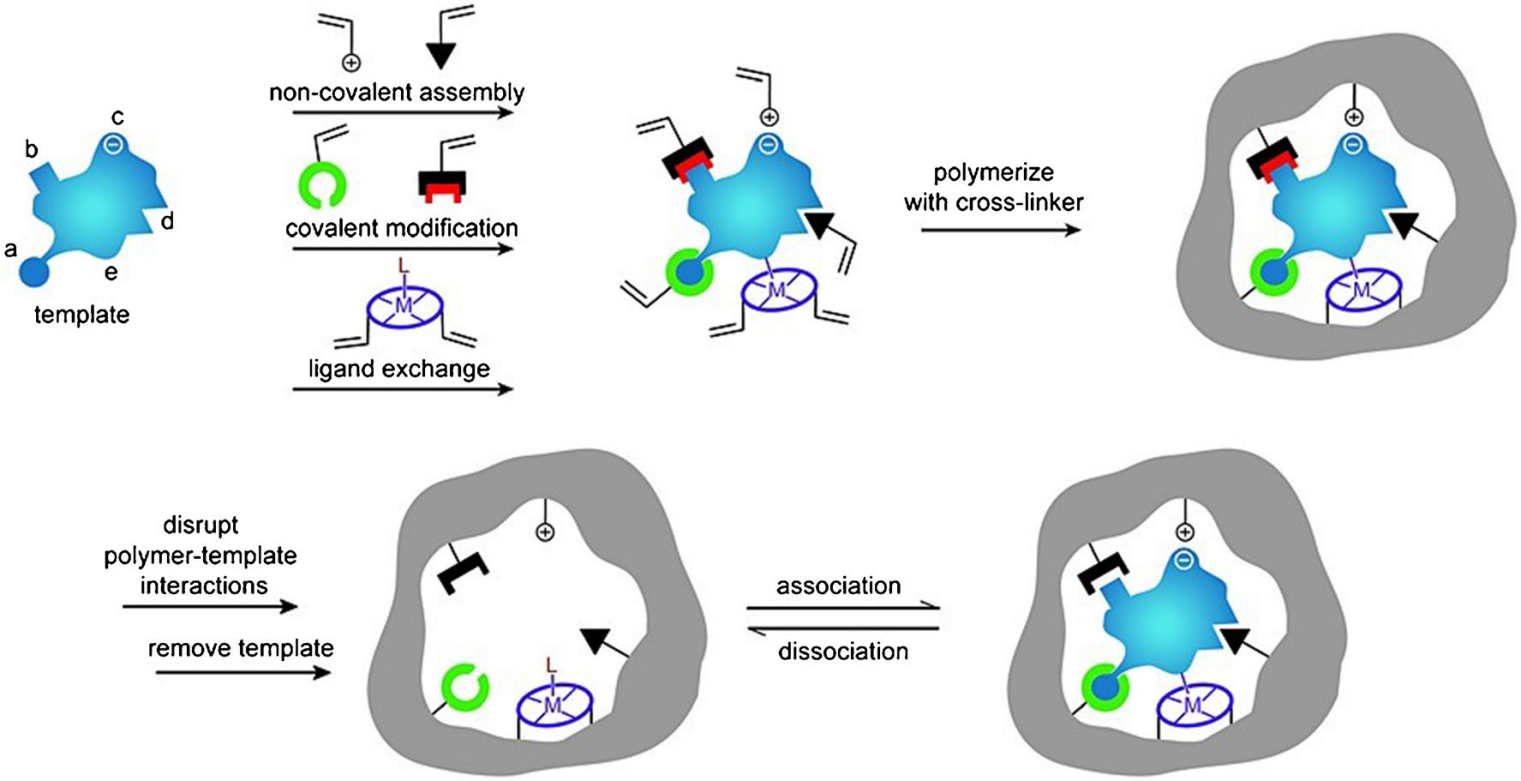
MIP preparation routes. The routes are described in the text. Reproduced with the kind permission of John Wiley and Sons from reference [63]

Molecularly imprinted polymers (MIP) have been devised as synthetic hosts able to provide increased affinity and specificity towards target/guest molecules. They mimic the biological systems from which they were inspired, with certain advantages in terms of chemical stability, long-term preservation of the strength and specificity of the binding sites, mechanical reliability, pressure and temperature durability, ease of preparation and low cost [60, 61]. However, they have some drawbacks (are large, rigid and insoluble) with respect to the biological receptors, which are usually smaller, flexible and mainly soluble [60]. The first approach to MIPs dates back to 1931, when Polyakov’s group succeeded in synthesizing silica gel with unusual adsorption properties towards benzene, toluene and xylene [62] (this work is briefly described in the ample review by Whitcombe et al. [63]). Since then, a huge number of investigations have been performed and their achievements reported in the literature. Several relevant review are available [12, 60, 61, 63–79]. Very roughly, the main idea behind MIP preparation is to add a template (target/guest molecule or a suitable substitute) and functionalized monomers to the MIP precursors to enable polymerization, and to remove the template after polymer synthesis. The resulting host material—the MIP—would have numerous suitably shaped cavities possessing the complementarity required in molecular recognition. There are a few dedicated routes for the manufacture of MIPs, which are sketched in Fig. 4.

The covalent route [80] relies on reversible covalent binding of the template with the reacting monomers. The stoichiometry is ensured by the covalent type of the bonds. After copolymerization with the cross-linker, the template is disrupted by chemical cleavage (acid hydrolysis for instance). Unfortunately, the number and variety of the MIPs obtained in this way is rather limited by the scarce choice of suitable templates and compatible monomers. The non-covalent route [81] uses a liquid phase process involving the template, functional monomers, cross-linker, initiator and a solvent. The template, which must be stable under preparation conditions, spontaneously binds the functionalized monomers (self-assembly process) by specific weak interactions. With the aid of the initiator, which provides free radicals for the early stages of the chain polymerization reaction when thermally or optically triggered, the template-functional complex copolymerizes with the cross-linker towards a template containing MIP. In a final preparation step, the template is removed by dissolution, and the MIP is able to rebind the template at the recognition sites. Besides the role played in the synthesis, controlling the morphology of the polymer matrix, the cross-linker stabilizes the imprinted binding locations and confers mechanical stability to the MIP, while the solvent determines its porosity [70]. There are a few other ways to realize the imprint: stoichiometric non-covalent approach, semi-covalent approach, metal ion-mediated approach [63] and the sol-gel route [82]. With the early polymer imprinting techniques, the choice of the reactants and reaction conditions was mainly a matter of “chemical intuition”, and the desired MIP specificity was gathered by long and unsystematic investigations, involving significant experimental efforts. The combinatorial methods [83] have improved the efficiency of the screening in molecular imprinting, making use of automated trial procedures. The rational MIP design, using thermodynamic and physicochemical foundations, constituted a real step forward in the field [84, 85]. The state of the art is represented by modeling of the template interaction with the possible functionalized monomers in the framework of molecular mechanics/dynamics, empirical/semiclassical quantum mechanics or ab initio quantomechanical formalisms (Hartree-Fock, Møllere-Plesset, DFT) [86–88]. In spite of the level reached by the theoretical and experimental approaches to MIP preparation, the specific recognition of target molecules is rather limited because of the relatively large amount of cross-linkers typically used in the synthesis routes, which allow for significant contributions from nonspecific binding mechanisms (mainly through dispersion interactions).

Fu and Finklea generated two types of shape-selective cavities (with hydroquinone and phenol non-covalently bound templates) in a poly(acrylic) or poly(methacrylic) polymer matrix [89]. The polymers were coated on the sensing TSMRs in a differential sensor system through an interfacing poly(isobutylene) film. Good response towards volatile organic compounds (VOC), linear calibration curves, short response (5/12 s) and acceptable recovery (11/90 s) times were achieved. The sensitivity was proportional (but not directly proportional) to the sensing layer thickness. This imprinting procedure conferred additional sensitivity and selectivity and changed the cross-sensitivity ratios among the tested analytes (trichloroethylene, benzene, toluene, heptane and carbon tetrachloride). Unfortunately, the humidity was omitted from the cross-sensitivity test. Starting from similar monomers, methacrylic acid and acrylamide, Bunte et al. obtained MIPs imprinted with 2,4,6-trinitrotoluene (TNT) and 2,4-dinitrotoluene (DNT) with high sensitivity and specificity [90]. An analogous approach was successfully used by Kikuchi et al. for the selective detection of terpenes (limonene, limonene oxide and α-pinene) [91]. From 4-vinyl pyridine (4VP), 1,4-divinyl benzene (DVB) and 2,2-azobis(isobutyronitrile) (AIBN) as functional monomer, cross-linker and initiator, Hwang et al. synthesized MIPs imprinted with acetoin and phenol for selective detection of isopropyl methyl ketone (IMK) and toluene, respectively [92].

#### Cavitand receptor materials

Compounds whose molecules contain internal free regions, such as porphyrins [93–95], metal-free^8^ phthalocyanines [96], calixarenes [97–100] and corroles [101], can in principle capture foreign atoms with appropriate size and chemical properties. For example, the cavity diameter of the calix[*n*]arenes with *n* = 4, 6, 8 influences the sensitivity towards chloroform, reaching the maximum for *n* = 8 [102]. Q-TSMR arrays coated with porphyrins have been successfully employed for the quality control of chocolate [103] and identification of microorganisms [104]. The approaches ware based on specific combinations of VOCs present in the vicinity of analyzed systems which could be recognized with multivariate data analysis (MDA). For all data evaluation methods^9^ addressed from here on, please refer to the section “Evaluating the performance of the gas sensors and sensor arrays” below. Another cavitand material type, GUMBOS (acronym for “group of uniform materials based on organic salts)” based on cyclic tetrapyrroles such as phthalocyanines and porphyrins, have been produced and used with quartz-TSMRs (Q-TSMRs) for detection of VOCs (methanol, ethanol, 1-propanol, 2-propanol, 1-butanol, acetone, chloroform, toluene, etc.) [105]. The phthalocyanines (Pc) without central atom(s) are currently not reported as gas-sensitive (by any transduction modality), while several metal Pcs have been successfully used for such purposes. One should note that in the case of metal–Pcs, or more generally, of all functionalized cavitands, the favorable chemical interaction can prevail over the geometric matching, and the ad/absorption does not take place, as expected, at the location with complementary shape, but at the chemically suitable sites. For instance, Harbeck et al. synthesized 2,3,9,10,16,17,23,24-octakis-(7,11-dioxaheptadecane-9-oxo) phthalocyanine and its Ni and Co derivatives and investigated their sensing potential towards several classes of VOCs with multi-reflection attenuated total reflection (ATR) Fourier transform infrared (FTIR) spectroscopy and TSMRs [106]. From the assignation and intensity of the ATR-FTIR bands relevant for the sensing process^10^ and TSMR large responses towards nonpolar VOCs (*n*-hexane), the authors deduced an analyte binding to the substituent alkoxy groups of the Pc receptor through van der Waals interactions. The polar compounds (methanol, ethanol, acetonitrile, ethyl acetate) were better detected with fluorinated alkyloxy-substituted Pcs [107, 108].

#### Functionalized receptor materials

It is difficult to find materials which have, at the same time, good compatibility with the transducer, increased physical and chemical stability, and good gas-sensing properties. The functionalized receptor materials are a class of materials generally possessing the first two requirements, but which gather the gas sensitivity/specificity either by attaching chemical active groups, particles or nanostructures, or through intentional modification of the local material structure. One always seeks better molecular complementarity of the receptor to the analyte and, by that, a convenient increase in the receptor performance. The functionalization can be made individually, using the chemical affinity of the added entities to the host material, or by devising classes of materials which provide functionalization sites and high gas sorption capabilities.

**Fig. 5:**
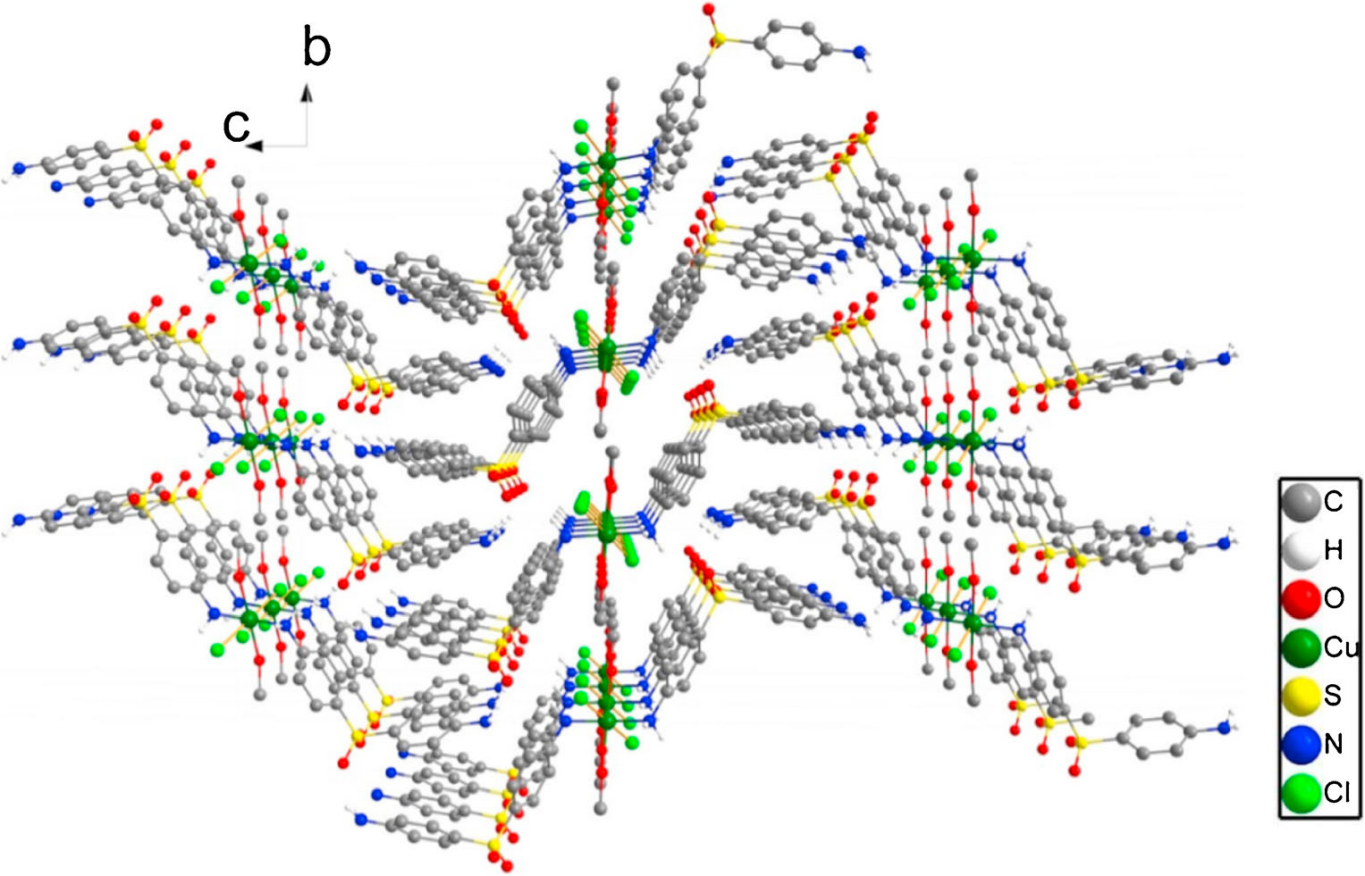
Stacked chart of the Cu(II) complex for formaldehyde detection synthesized by Wang et al. Reproduced with the kind permission of Elsevier B.V. from reference [120]

*Functionalization of different plain host materials.* Historically, the specific functionalization was the first modality employed by the researchers to improve the gas-sensing performance of plain sensing polymers, cavitands and two-dimensional (2D) or one-dimensional (1D) materials [109, 110]. A systematic investigation on poly(siloxanes) by Hierlemann et al. proved that a suitable choice of the side groups attached to the polymers increases the partition ratio of the targeted analytes [111]. Many researchers have reported good results in gas/vapor sensing with modified polymers [112–114]. To upgrade the sensing performances of cavitands, they were functionalized with side groups like tetra-tert-butyl [115] and alkoxy [106] for phthalocyanines or dihydroxyphenyl for corroles [101], or combined in complex compounds/mixtures [93, 95, 105, 116]. Even though low-dimensional materials have been regarded as intrinsically more sensitive and selective than bulk materials, many researchers are striving to further improve their sensitive features by attaching different nanoparticles or functional structures. Zong et al. successfully functionalized hollow mesoporous silica spheres with poly(dopamine) to detect formaldehyde released from food 11 [117]. Bonding p-hexa-fluoroisopropanol aniline to mesoporous TiO_2_-SiO_2_, Zhu et al. measured concentrations of nerve agent simulant dimethyl methyl phosphonate (DMMP) down to 100 ppbv [118]. The sensing material was coated on Q-TSMRs. Data regarding the influence of humidity on the sensor response are missing. Ogimoto et al. demonstrated the suitability of mesoporous functionalized SiO_2_ nanoparticle and films for the detection of low levels of ammonia in human breath [119]. A Cu(II) complex [Cu(DDS)_2_(Cl)2(MeOH)_2_]^12^ ( see Fig. 5) for formaldehyde sensing was designed and synthesized by Wang et al. directly on Cu-coated Q-TSMRs, ensuring intimate coupling between receptor and transducers [120]. According to the authors’ DFT calculations, the reported sensitivity (LDL of 50ppbv) and selectivity are due to the reversible binding of formaldehyde on the amino groups of the complex compound. The reported low cross-sensitivity to humidity is misleading because the water concentration in the test mixture was only 100ppmv.

**Fig. 6:**
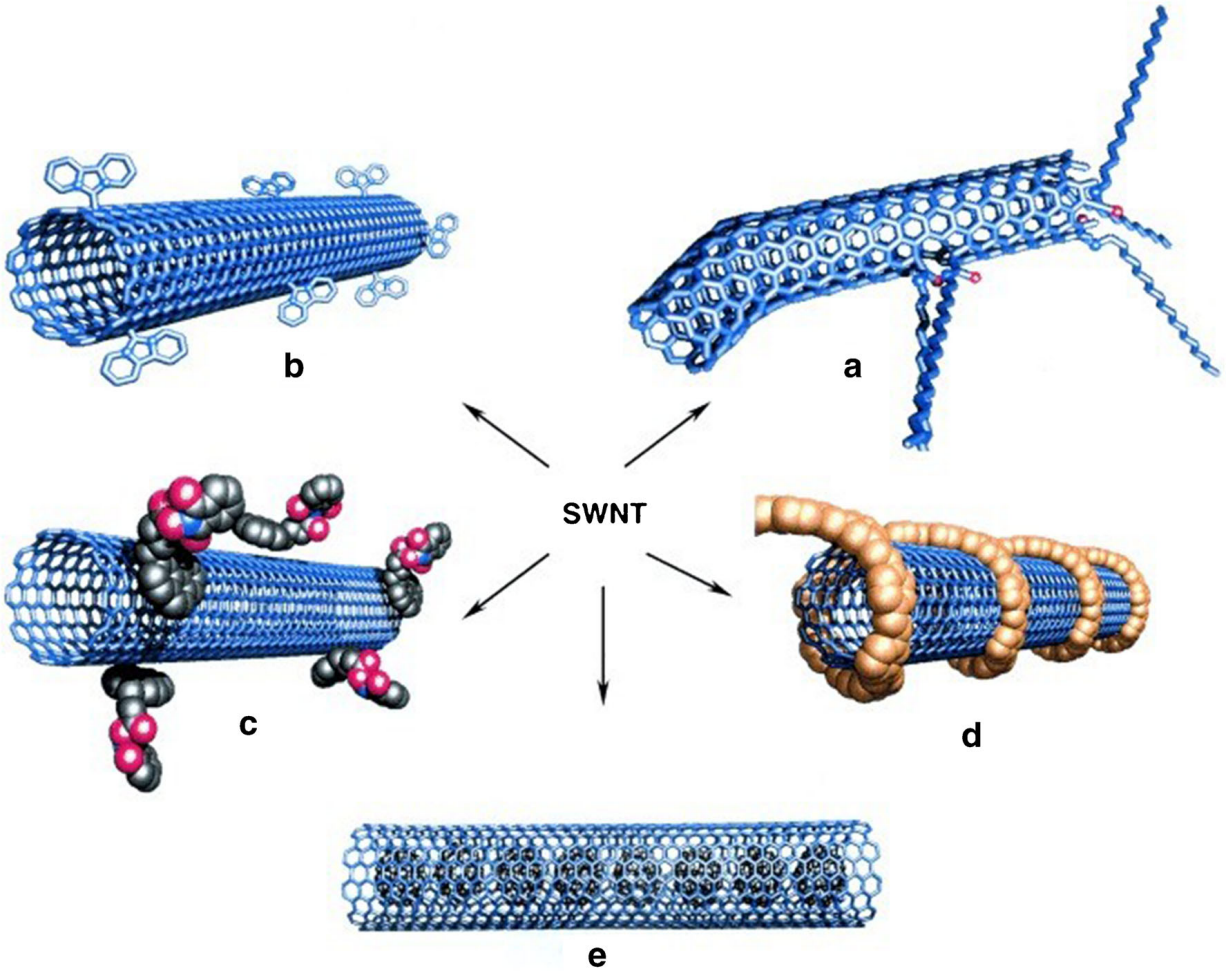
“Different functionalization approaches for single-wall nanotubes (SWNT) of carbon. **A**) defect-group functionalization, **B**) covalent sidewall functionalization, **C**) non-covalent exohedral functionalization with surfactants, **D**) non-covalent exohedral functionalization with polymers, and **E**) endohedral functionalization with, for example, C60. For methods B ± E, the tubes are drawn in idealized fashion, but defects are found in real situations” (original caption). Reproduced with the kind permission of Wiley VCH from reference [132]

Although they are good conductors, low-dimensional carbon materials^13^ [121] like carbon nanotubes (CNT) [122–124], graphene [125–129] and reduced graphene oxide (RGO) [130, 131] are often functionalized [132] (see Fig. 6) [133], and used not only for conductometric [134] or field-effect [135] gas sensors, but also for gravimetric ones. Asad et al. devised SAW H_2_S gas sensors based on single-walled CNTs decorated with Cu [136]. These GGSs have LDL below 1 ppmv at room temperature and a reduced cross-sensitivity to humidity (2 ppmv H_2_S roughly corresponds to 40% relative humidity [RH])^14^. Phthalocyanine- and porphin-functionalized CNTs were used by Ndiaye et al. to detect aromatic VOCs with detection limits below the threshold limit values (TLV)/time-weighted averages (TWA) for these analytes [137]. RGO is the most widely used low-dimensional carbon-based material for gas sensing, mainly functionalized. Yu et al. employed Au nanoparticles (AuNP) to upgrade porous sheets of RGO coated on micro-cantilevers towards VOC detection [138]. The sensors were selective for trimethylamine (TMA). The authors reported a lower influence of 100 ppmv humidity on the response of AuNP-RGO sensing layers (0.4 Hz) than for AuNP-GO layers (2.8Hz). However, when extrapolating to 50% RH, as typically present in the environment, one gets a frequency shift of ~40 Hz, which is five times larger than the response to 10 ppmv TMA (roughly the TLV-TWA for this analyte). Almost the same group of authors used carboxyl-functionalized AuNPs grown in situ on RGO for NH_3_ sensing with a similar cantilever [139]. The selectivity to ammonia was satisfactory at the evaluated concentration of 300 ppmv, but again one obtains sensor responses extrapolated to 50% RH larger by an order of magnitude than TLV-TWA of NH_3_ (~30 ppmv).

*Functionalization of specially devised receptor materials*. Two large categories are relevant here, the zeolites [140] and the porous coordinative polymers [141], usually addressed as metallo-organic frameworks (MOF). Except for natural zeolites, which are increasingly less used for gas sensing, these materials are synthesized to target the required properties for the desired application^15^. Often, established members of these families are functionalized and utilized as receptor materials. The zeolites are crystalline materials (aluminosilicates and similar ones) with regular nano and meso porosity, having a rigid three-dimensional (3D) structure [153]. Though encountered as natural mineral, for the purposes of gas sensing [154, 155] they are chemically synthesized [140] and coated on transducer devices [156]. Urbiztondo et al. tuned the properties of zeolites either through synthesis or by modifying commercial products through ion exchange before deposition as colloidal suspensions on cantilevers [157]. A detection limit of ~0.5ppmv o-trinitrotoluene was obtained with the Co^2+^-BEA zeolite (for BEA zeolite see reference [156]). Using a La-modified AlPO-5 zeolite, Wang et al. measured NH_3_ concentrations down to 60 ppbv [158]. The electrodeless Q-TSMR was read out in a wireless setup. A significant influence (about a factor 2) of the background humidity on the responses towards ammonia might limit the sensor applicability.

MOFs were devised to expand the capabilities of zeolites. In the MOF structure, metal ions or clusters are linked by organic ligands [143, 144, 159–163]. The MOFs became interesting for chemical sensing [164, 165] in general and for the particular case of gas sensing [166–169] due to their large specific area and selective gas adsorption, especially when functionalized [163, 170–172]. Lv et al. coated micro-cantilevers with Ni-MOF-74^16^ and succeeded in sensitive detection of CO (LDL=10 ppbv) in the absence of humidity. The same group of authors used home-synthesized MOF-5^17^ to detect aniline with an LDL lower than 1,4 ppmv, also with micro-cantilever transducers [174]. However, the cross-sensitivity to humidity was two orders of magnitude larger. Yamagiwa et al. reported the detection of VOCs with [Cu_3_(BTC)_2_(H_2_O)_3_]∙xH_2_O (BTC = 1,3,5-benzenetricarboxylate) and [Zn_4_O(BDC)_3_] (BDC = 1,4-benzenedicarboxylate) [175]. The transducers employed were Q-TSMRs or silicon micro-cantilevers. The sensitivity enabled measurements in the TLV-TWA range for the selected analytes, but the selectivity, expressed through comparative absorption isotherms only, seems to have been rather poor. The influence of humidity was not addressed. He et al. synthesized a covalent organic framework (COF) through the Schiff base condensation of 1-(4,7-bis(4-aminophenyl)-1H-benzoimidazole-2-yl)ethan-1-ol (BABE) with 1,3,6,8-tetrakis(4-formylphenyl)pyrene (TFPy), which they abbreviated BABE-TFPy COF [176]. It was sensitive (LDL~ 1 ppmv) to 2-chloroethyl ethyl sulphide (CEES), a mustard gas simulant. DFT calculations revealed evidence of a double hydrogen bond between the receptor material and the analyte (one between the OH group of BABE and thioether of CEES and another between the NH group of BABE and Cl of CEES), which provided good selectivity excepting humidity^18^. Wang et al. reported high sensitivity (100Hz/ppmv, LDL~60ppbv) towards ammonia for a La-doped AlPO-5 framework (refer to [153] for the material) coated on TSMR transducers [158]. The logarithmic dependence of the calibration curve in the TLV-TWA range (25 ppmv) would not be a real issue, but the high cross-sensitivity to humidity (rather doubling the response to ammonia) requires hardware/software compensation. General investigations on the kinetics of the gas uptake in ultra-microporous frameworks with fast SAW sensors were reported by Paschke et al. [177]. The authors showed the ability to infer the diffusion rates of gases in the addressed sensing materials.

#### Composite, polymorph and unusual receptor materials

Composite receptor materials are customarily prepared from different classes of compounds with dissimilar properties. Amorphous materials, which do not stress the MST, can be mixed with rigid or less adhesive materials to obtain better sensor performance. Such an approach is even more relevant for dielectric or chemoresistive sensors, where the components of the sensing mixture can individually perform the reception and transduction functions. The composite film from cellulose acetate and a representative compound (1-*n*-butyl-2,3-dimethylimidazolium hexafluorophosphate) of GUMBOS are sensitive to VOCs and have been utilized as GGS with a TSMR transducer [178]. Lal and Tiwari demonstrated the suitability of poly(epichlorohydrin) (PECH) alkyd resin used as composite with nanaoclay for the selective recognition of chemical warfare agent (CWA) simulants (1-chloro-2-[(2-chloroethyl)sulfanyl]ethane (SM) and DMMP [179]. In a more sophisticated approach, Chen et al. proposed a mixture consisting of MIP (methacrylic acid with ethylene glycol dimethacrylate copolymer imprinted with hexanal) and hydrophobic silica nanoparticles [180]. Besides high specificity for hexanal, the receptor exhibited low cross-sensitivity to humidity (visible on the dynamic response with humidity in background, but not certified in a dedicated experiment with incremental humidity). A GO and poly(styrene) composite was found to be sensitive to ammonia down to a few ppmv [181]. CGO/chitosan nanocomposites for amine vapors were reported by Zhang et al. [182]. Sensitivity of 2–5 Hz/ppmv with Q-TSMR transducers, LDL below 3 ppmv and rather low cross-sensitivity to other VOCs would make these sensors appropriate for amine detection, but the large influence of background humidity is a real issue. SAW transducers covered with ZnO/SiO_2_ composite films were employed by Wang et al. to detect ammonia well below 10 ppmv. The sensing mechanism ascertained by the authors involves a charge transfer between the analyte and ZnO from the sensing layer, inducing an increase in the sensing layer conductivity, and by that, a change in the resonance frequency of the transducer^19^. This should explain the low cross-sensitivity to other tested gases. However, the influence of the background humidity on the sensor response was not discussed and the sensing mechanism was not experimentally proved. A protonated poly(ethylenimine)-graphene oxide (P-PEI-GO) nanocomposite thin film was deposited by dipping onto Q-TSMRs for humidity measurements by Tai et al. [183]. The sensitivity increased linearly with the number of coated layers, but was strongly nonlinear with respect to humidity. The sensing layer stability over 30 days was good.

**Fig. 7:**
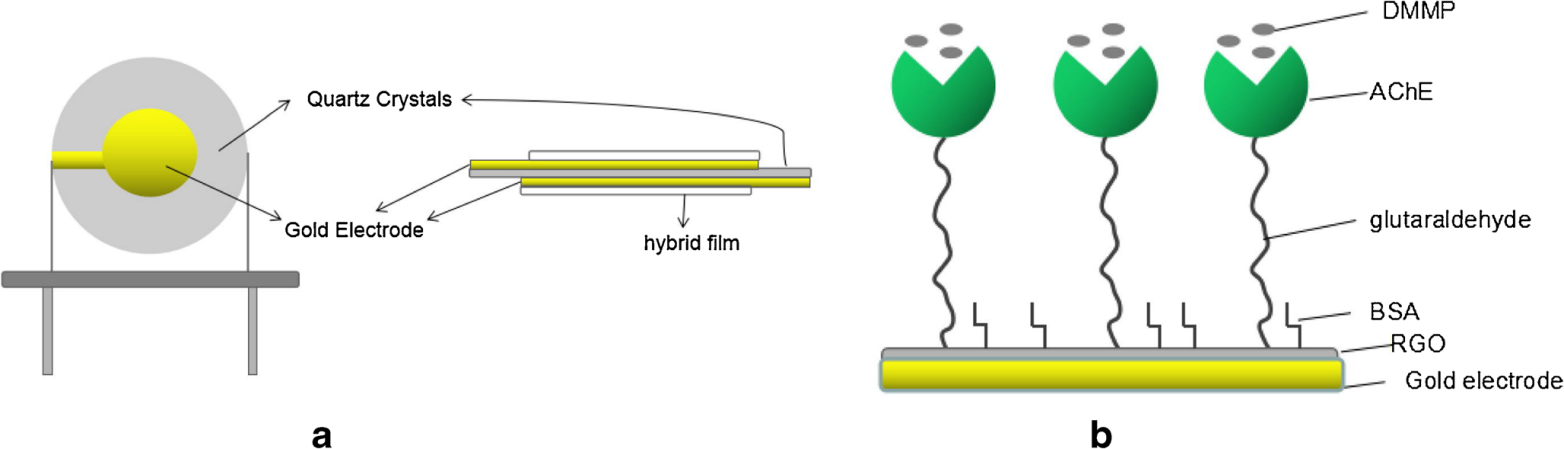
Polymorph layered receptor based on reduced graphene oxide and acetylcholinesterase for the detection of dimethyl methyl phosphonate. **a**. The GGS sketch. **b**. The polymorph structure. Reproduced with the kind permission of Elsevier B.V. from reference [184]

The polymorph receptors consisted of several thin layers and were used to better^20^ attach the sensing material to the transducer surface. Biological layers (acetylcholinesterase [AChE]) immobilized on TSMRs previously coated with reduced graphene oxide (RGO) were successfully employed for gas sensing (CWA simulant DMMP) [184]. Figure 7 illustrates this bimorph sensing film. GO and ZnO layered receptors deposited by Yuan et al. on TSMRs showed increased sensitivity and fast response to humidity [185]. Almost the same group of authors reported on RH evaluation with GO/poly(ethyleneimine), with similar performance [186]. In order to strongly reduce the cross-sensitivity to humidity of the poly(dopamine) (PDA) sensing layer for formaldehyde GGSs, Wang et al. prepared a superhydrophobic coating of PDA with polymerized *n*-octadecylsiloxane (PODS) nanostructures [187]. The bimorph receptor displayed good immunity to water vapor (contact angles to water larger than 140°). Its response towards 97% RH was equal to the one to 5 ppmv HCHO only, preserving at the same time the HCHO sensitivity.

Ionic liquids have also been tested as sensing materials [188–190]. In the case of six imidazolium-based ionic liquids, good signals and specificity to ethanol were achieved [190]. Unfortunately, an inappropriate choice of the concentrations for humidity tests hid a huge cross-sensitivity to this analyte. Reference [188] demonstrates the discrimination of VOCs with high-temperature ionic liquids. Linear discriminant analysis (LDA) gave good results, but in the absence of humidity.

### Detection of enantiomers in the gas phase

**Fig. 8:**
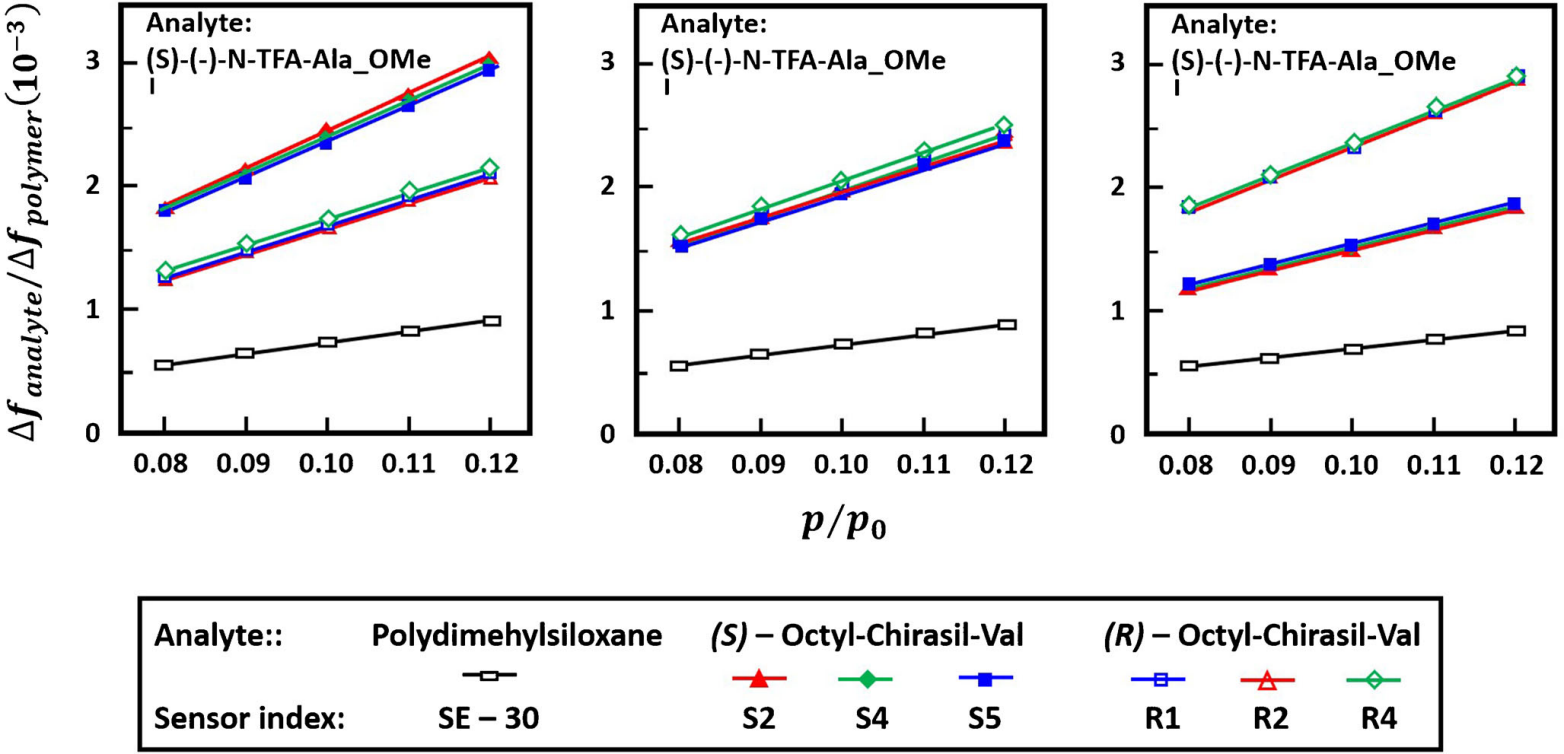
“Normalized (with regard to the frequency shift due to polymer deposition) TSMR responses to different concentrations (p/p0: adjusted pressure with respect to the saturation vapor pressure at 298K) of (R)-, (S)- and racemic *N*-trifluoroacetyl-alanin methyl ester. The responses of three (R)- (open symbols), three (S)- sensors (filled symbols) and one SE-30 sensor are displayed. Sensor index is given at the bottom” (original caption). Reproduced (the remake was necessary due to reduced resolution of original figure, whose faithful copy is) after [206]. The use of the figure was allowed by Springer Nature

For analytes possessing chiral symmetry [191–193], discrimination of the enantiomers in gaseous mixtures is possible by using receptors with complementary chirality, as generally accounted for by molecular recognition principles [12, 80, 194–201]. The sensing materials employed to achieve this aim largely belong to classes already addressed above. They are either suitably chosen or specially devised [163, 202–204]. Making use of receptors based either on both enantiomers of Chirasil-Val derivatives [205, 206] or on cyclodextrin derivatives [207], Bodenhöfer et al. were able to selectively detect the enantiomers of amino acids and lactate, and the chiral gaseous anesthetics isoflurane, enflurane and desflurane, respectively. In all cases the transducer was a TSMR. These authors also evaluated the chiral discrimination factors^21^ of the receptors for the given analytes and the changes in the corresponding differences in the sorption free enthalpies, enthalpies and entropies^22^. The detection specificity for the R (right/rechtus) and S (left/sinister) enantiomers of *N*-trifluoroacetyl-alanin methyl ester (N-TFA-Ala-Ome) with enantioselective receptors like (R, S)-octyl-Chirasil-Val is shown in Fig. 8. The exposure to racemic mixtures led to similar responses from both types of receptors. The enantiomeric discrimination factor was found to be^23^, in mean value, *α*_*Sensor*_ = 1.6,3 and the corresponding enantiomeric difference of the free enthalpy, ∆∆*G*^0^ =  −1050 ∓ 100 *J*/*mol  at* 303*K*. A witness polydimethylsiloxane-coated TSMR had low and nonselective responses.

The chiral discrimination ability of cyclodextrins has been extensively addressed in the literature, and the discrimination features (discrimination factors and differential thermodynamic parameters) acquired in gravimetric, calorimetric, chromatographic and nuclear magnetic resonance (NMR) experiments have been reported [2, 208–212]. Other cavitands, such as porphyrins, have also been considered as enantioselective receptors, either alone [213] or in association with other materials [214]. The advances in the synthesis/preparation of gas-sensing materials brought into play more diverse chiral receptors: macrocyclic arenes [100], metal-organic cages/containers [204], metal-organic frameworks [163, 171] and thin film organic frameworks [145]. An extended review dedicated to the use of stereoregular chiral polymers for the separation of enantiomers (the liquid phase is also largely addressed therein) was elaborated by Shen and Okamoto [215].

### Gravimetric sensor systems

The experimental results obtained with the selectivity strategies listed above did not fully confirm the optimistic expectations they raised because of the unspecific interactions which still contributed significantly to the sensor response. Therefore, gravimetric sensor systems (GSS), containing sensors with different specificity and, possibly, hardware for online data processing, have been seen as an effective way to address the selectivity problems that typically occur in the gas-sensing field. Since the variability in the responses of a sensor system is much greater than that coming from a single sensor, diverse approaches have been employed to build GSSs. The sensor array (SA) is the simplest system variant, in which either discrete sensors are assembled mechanically and electrically together, or integrated sensors are micro-machined on a single chip [216–219]. The degree of sensor signal processing at the array level is limited and usually lacks the chemical recognition facility. Electronic noses (EN) are instruments/devices encompassing analyte sampling stages, large and complex sensor arrays, readout and processing electronics, and chemometric software [43, 220–225]. They are able to detect and recognize odors and flavors and their individual chemical components in the gas phase. Several examples of gravimetric SAs reported in the literature are given in the following (the first of them for a historical perspective). The chemometric methods they employ will be reviewed in the section “Evaluating the performance of the gas sensors and sensor arrays”. A discrete SA of TSMRs coated with six different poly(siloxanes) was employed by Hierlemann et al. to identify and quantify hazardous VOCs [226]. The values predicted by the array in test events (using partial least squares regression [PLSR]) were in good agreement with the true values. Kim et al. utilized polymer-coated cantilever arrays for qualitative and quantitative analysis of VOC mixtures [227]. The array output data were also evaluated with PLSR. Micro-machined cantilever arrays integrated on silicon chips using complementary metal–oxide–semiconductor (CMOS) technology were reported by Lange et al. [216]. They combine the advantage of direct signal amplification/processing with the increased discriminatory power enabled by multiple devices and sensing coatings. Thus, different features of the analytes and receptors are used simultaneously, and the reception specificity increases. Dickert et al. demonstrated the selective detection of 0–200 ppmv xylene in a common humidity background (up to 60% RH) with Q-TSMR arrays coated with compounds providing molecular complementarity to the targeted analytes [228]. The hardware sensing system was supported by MDA (PLSR) and artificial neural networks (ANN). The influence of humidity was practically rejected by the numerical algorithms. Discrete Love-wave sensor arrays coated with selected polymers (three poly(siloxanes), poly(ethyleneimine), poly(epichlorohydrin) and Carbowax) enabled the sensitive detection of DMMP (down to 40 ppbv) and good CWA discrimination by PCA combined with probabilistic neural networks (PNN) [229]. No false assignations occurred among the reported events. Roughly the same approach was employed by Senesac et al. for 11 inorganic and organic vapors and gases [230]. The responses of the 10 cantilevers in the array were processed by a back-propagation (BP) ANN. A comparison of gravimetric and chemoresistive SAs based on polycyclic aromatic hydrocarbons with different side groups was reported by Bachar et al. [231]. Both types of arrays provided consistent results when assisted by PCA and discriminant factor analysis (DFA). Lu et al. reported on microfabricated FBAR sensor arrays coated with cavitands (calix[8]arene, porphyrin, β-cyclodextrin, cucurbit[8]uril) for selective VOC detection [232]. The authors assessed the suitability of their devices for an integrated electronic nose, but they did not fabricate the EN or use MDA in their investigation. The exposure to the analytes (chloroform, acetone, methanol, hexane, etc.) was performed at very high concentrations (10% to 100% from each saturation vapor pressure) without background humidity. Though nice, the results seem not to be relevant for the conditions encountered in real applications. In a different approach, Mascini et al. demonstrated the possibility to “tailor” sensors for gas-sensing arrays using the virtual screening of a large database of tripeptides (8000 elements) in virtual interaction (molecular docking simulations) with VOCs (58 vapors) from five chemical classes [57]. Then, using a combinatorial method, 120 tripeptides with the highest interaction specificity were further employed to generate ~7900 virtual tetrapeptides, from which five were selected, prepared and covalently attached to gold nanoparticles and coated on TSMRs. The data outputted by the real arrays during gas exposure were evaluated in the PCA frame, confirming the good gas-sensing performance suggested by the design.

Based on atomistic molecular simulations^24^, Gustafson and Wilmer estimated the best choice among nine potential MOF receptors for an array theoretically designed to discriminate and recognize different target gases [234]. To illustrate the approach, CH_4_, N_2_ and O_2_ were selected. The aim was to reduce the effort spent in trial-and-error experiments and to improve the host–guest matching of the analyte–receptor pair, on the one hand, and to reduce the dimensionality^25^ of the array, on the other hand. The SA was numerically tuned for CH_4_ detection, but any target analyte is in principle eligible. Complementing somewhat the virtual screening of receptor materials, Speller et al. devised a hardware virtual sensor array whose virtual sensors were the overtone responses of single Q-TSMR GGSs [236, 237]. A virtual sensor array for VOCs was obtained by Zeng et al., modulating the temperature of a film bulk acoustic wave (FBAW) transducer covered with self-assembled organic films [238]. The exposure events to the same analyte at different partial pressures appeared as straight lines in the first two principal components (PC). Dissimilar analytes were separated mainly along PC1 and clustered very well when classified with LDA. Chen et al. realized a SAW virtual array, adding a chromatographic-like column to a SAW sensor and evaluating the retention times for different analytes specific to lung cancer [239]. A very ingenious paper cantilever array functionalized with polymers, operating in deflection mode and with a visual readout, was devised and tested by Fraiwan et al. [240]. The LDA canonical score plot proved the complete separation of the clusters for acetone, methanol, ethanol and tetrahydrofuran. Many other authors report the successful use of more or less conventional GGS arrays to solve analytical problems in gas detection: ionic liquids Q-TSMRs for VOCs [241–244] or for explosive vapors/gases [245], SiO_2_-NP functionalized with organic materials Q-TSMRs for breath analysis [246], AuNP-peptide Q-TSMRs for food aroma detection [51], poly(acrylic acid) MIP on Q-TSMRs for aldehyde [247] and organic acids [248] in body odor, peptide-modified ZnO-NP on Q-TSMRs for organic VOCs [249], biomimetic MIPs on Q-TSMRs for terpenes from herbs [250], GO and *N*-substituted pyrrole derivative-based films on Q-TSMRs for toxic gases (CO, NH_3_ and NO_2_) [251], metallo-porphyrins and AuNP-peptide on Q-TSMRs for chocolate quality control, [103], SWCNT/organic materials on Q-TSMRs for ambient air composition (as example) [252], porphyrin Q-TSMRs for vapor released by microorganisms [104], polymer films on Si cantilevers for VOCs, [253, 254] and MIP on SAW for CWA detection [255]. In order to increase the accuracy of gas sensing, arrays of GGSs together with other types of gas sensors have been devised and tested. They will not be fully addressed in this review, but examples include integrated micro-cantilevers with micro-calorimeters and capacitors [256–258], Q-TSMRs with chemo-resistors [231, 259], SAW, Q-TSMRs and silica optical fiber [260]. An extended overview is available in the table as Annex.

The studies in the field of EN are quite old, some of them resulting in commercial instrumentation [220]. A modular hybrid EN (MOSES ll) was devised by Ulmer et al., including QTSMRs together with metal oxide, electrochemical and calorimetric sensors [261]. It uses an Agilent/HP 7694 headspace autosampler when required. In evaluation tests, the VOC mixtures, coffee and tobacco flavors and other odors were well separated and recognized. McGill et al. reported an EN (NRL-SAWRHINO) based on SAW sensing devices coated with functionalized polymers for CWA [262]. The instrument employs, as a first stage, a trap-and-purge gas–solid chromatographic column. The discrimination and clustering in the first two principal components of the nerve and blister chemical agents, with various interferents in the background, was error-free. An EN using a chromatographic column and poly(isobutylene) virtual SAW array was developed by Chen et al. to detect lung cancer through 11 marker VOCs [239]. The postprocessing of the signals was conducted using a BP-ANN which delivered a graphical output towards an image recognition approach. There was no incorrect disease identification, but one ill and one healthy person (from 5 ill and 5 healthy) were categorized as suspects only. Starting from an eight-cantilever array functionalized with polymers, Lang et al. [263] built an EN for fragrance characterization and identification of disease-specific odors. The transducers were operated in proportional bending mode, induced by swelling of the sensing layers, with optical readout. The acquisition of beam deflection amplitudes at five successive time points during the exposure increased the dimension of the array output to 40. The nose was able to separate very well, in the plane of the first two principal components, the common VOCs, different natural scents and VOCs specific to diseases. With a cantilever EN employing ANN, Leis et al. were able to detect DMMP in ppbv range in ternary mixtures with water and ethanol in ppmv ranges [264]. Although scientifically relevant, this performance would not be enough in a practical application, where the humidity is much higher. Fernandes et al. used a SAW-EN to detect VOCs [265]. The PCA discrimination for equal analyte concentrations (50 ppmv) in mixtures was good, but when the concentrations were spread over large ranges, the clusters overlapped. A chromatographic column connected to Q-TSMRs was utilized by Rivai et al. to construct an EN for VOCs and odors [266]. Electronic signal conditioning and ANN pattern recognition allowed the authors to map a large number of odors and to discern more the 20 of them in a 2D PCA plot. Magna et al. utilized gas specificity induced by the preparation route of porphyrin-functionalized ZnO (coated on Q-TSMRs) to enable selective operation of an EN designed to detect VOCs [94]. Some studies addressing both sensor SA and EN were already considered in the SA paragraph above, while reports on practical applications with EN will be referred to in the dedicated section “Targeted analytes and applications”. Pertinent reviews devoted to bioelectronic noses, related biomaterials and artificial olfaction were published not too long ago by Wasilewski et al. [46, 224] and Barbosa et al. [45]. There are also studies investigating accessories for SA and EN as pre-concentration units [267, 268] or ASIC interfaces for TSMRs and micro-resistors (μR) [269]. In the literature, reviews can be found dedicated to gravimetric sensors, SA and EN based on given types of MSTs [219, 222, 270, 271] for instance.

## Deposition methods for sensing layers on MSTs

**Fig. 9:**
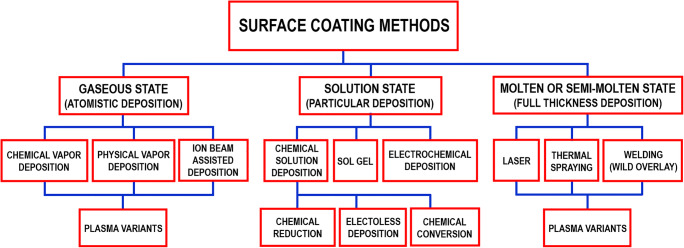
Schematic overview of several surface coating methods. Reproduced (the remake was necessary due to reduced resolution of original figure, whose faithful copy is) with the kind permission of the authors and AIMS Press from reference [272]

The most appropriate receptors for MSTs are thin sensing layers firmly attached to the transducer surface. The thickness should fit the MST operating requirements addressed in the section “Mass-sensitive transducers” of the first review part. Therefore, coating procedures for thin films are largely employed, and are well surveyed by the general literature [272–279]. A schematic overview of several surface coating methods as proposed by Oluwatosin Abegunde et al. is included in Fig. 9 [272].

A few of these will be referred to below, with additional references. A complete overview of the most commonly employed methods for the manufacture of GGSs is contained in Annex, where they are reported together with sensor performance.

**Fig. 10:**
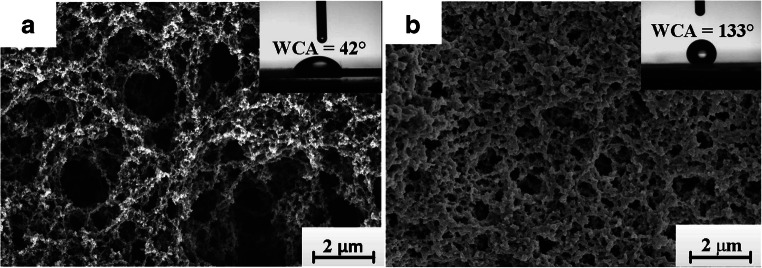
Hexanal MIP (**a**) and hydrophobic hexanal MIP–SiO_2_ NPs composite (**b**) deposited by drop coating. The inset pictures indicate the water contact angles (WCA) of the films. Reproduced with the kind permission of Elsevier B.V. from reference [180]

**Dip and drop coating/casting** are the simplest coating methods, but have lower precision and result in large spreads of the morphology and geometric parameters of the deposited layers [280]. Therefore, they are mainly utilized in the incipient phases of an investigation. Automated variants of the methods can enable the manufacture of less demanding films. Das et al. [281] reported a process involving dipping followed by polymerization to coat castor oil with different amounts of benzoyl peroxide on TSMRs sensitive to aliphatic amine vapors. Ayad et al. used drop casting to prepare chitosan/poly(aniline) nanofibers for TSMR detection of methyl/dimethyl amine and ethanol [282]. Poly(aniline) emeraldine salt thin films doped with different acids were also deposited by dip coating on TSMRs as VOC sensors [283]. Figure 10 shows the morphology of drop-coated Q-TSNRs with hexanal MIPs and their composite with hydrophobic silica, as prepared by Chen et al. [180].

**Fig. 11:**
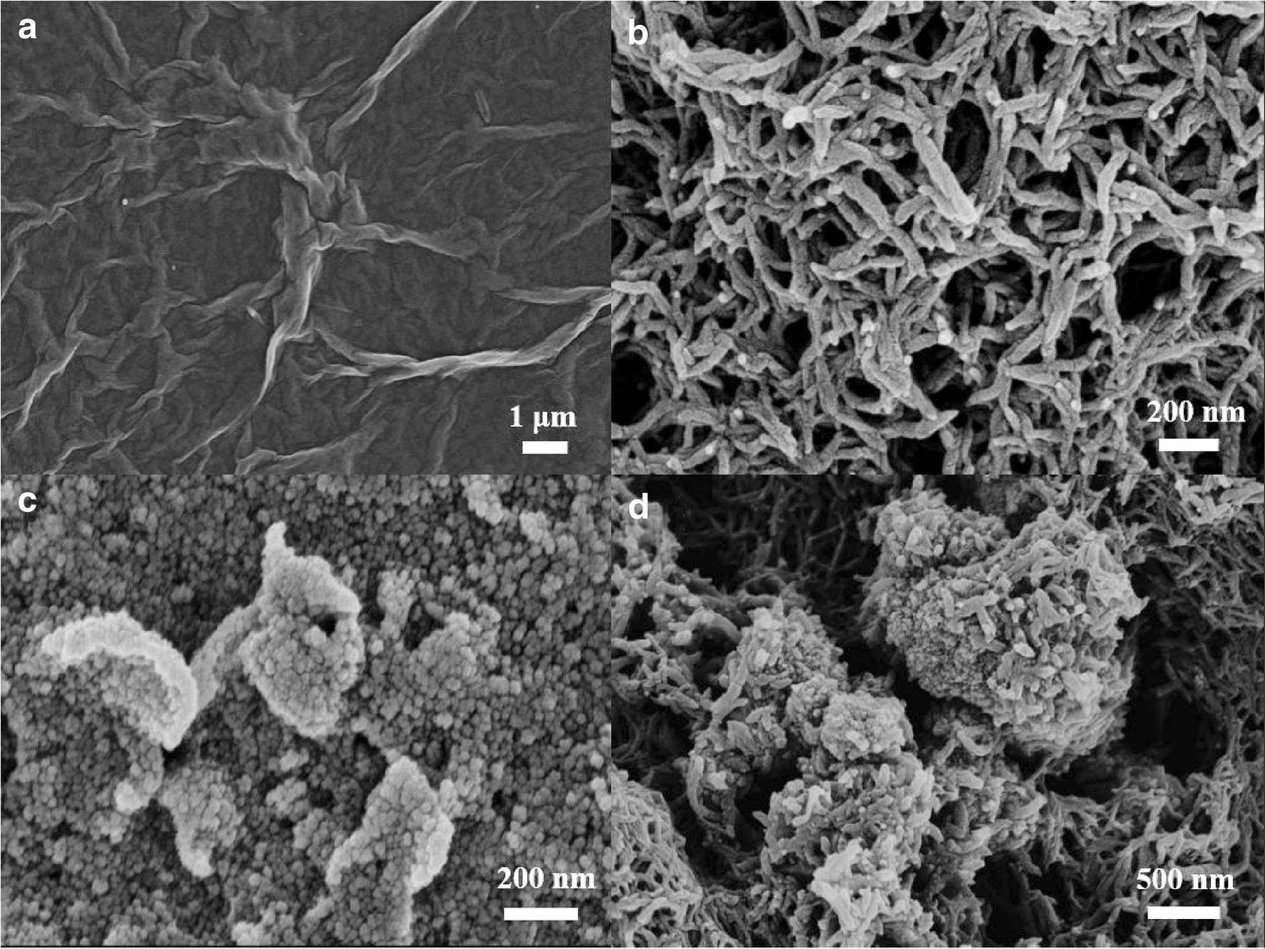
The morphology of GO (**a**), PANI (**b**) and GO/SnO_2_/PANI (**c**), (**d**) deposited by airbrush spray coating on Ag Q-TSMR electrode. Reproduced with the kind permission of Elsevier B.V. from reference [287]

**Fig. 12:**
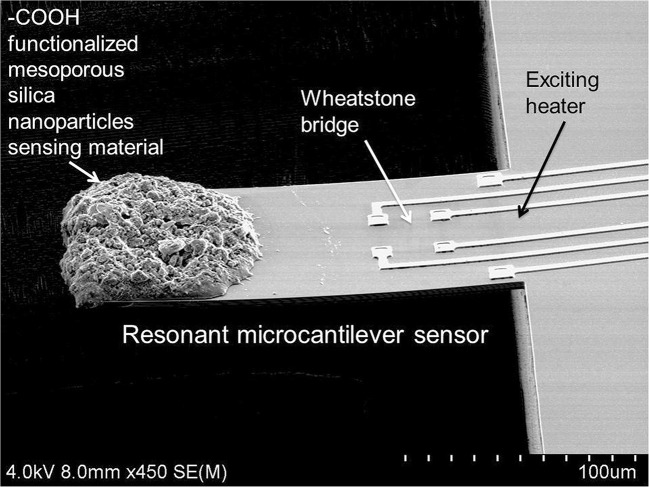
SEM image of carboxyl group-functionalized mesoporous silica nanoparticles (C-MSNs) inkjet-printed on the active area of a Si micro-cantilever. Reproduced with the kind permission of Elsevier B.V. from reference [289]

**Fig. 13:**
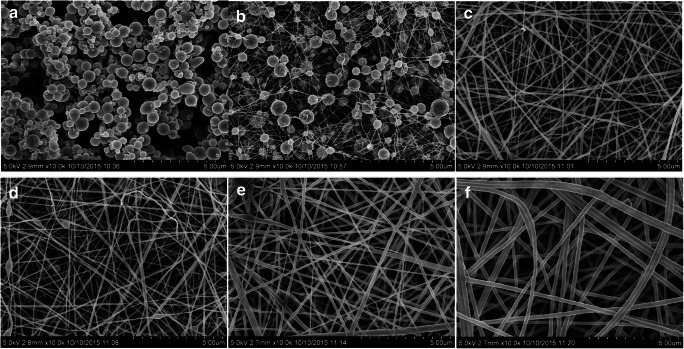
FE-SEM of PAA layers deposited by electrospinning/electrospray: NPs (**a**), bead and string (**b**), fibers (**c**–**f**) from different solutions. Reproduced with the kind permission of Elsevier B.V. from reference [295]

**Spray coating, inkjet printing, electrospray coating, spin coating and electrospinning** enable the covering of surfaces with materials solubilized or suspended as powders in carrier solvents. Through spray coating, solutions/suspensions are transformed into aerosol flow by a nozzle [284–286]. Spray coating is very popular for polymeric coating of TSMRs [185, 186]. Sometimes a manual version, airbrush coating, is preferred [243, 287]. The morphology of a layer family (GO, PANI, and GO/SnO_2_/PANI) deposited by airbrush spray coating on an Ag Q-TSMR electrode by Zhang et al. is shown in Fig. 11 [287]. Humidity evaluation with this layer is addressed in the paragraph “Humidity” below. Inkjet printing is the modern version of spray coating inspired by paper printing technologies [288]. It allows for better control of the coated area and film thickness and porosity, being suitable for small devices where precision is a key requirement. Figure 12 shows the SEM image of carboxyl group-functionalized mesoporous silica nanoparticles (C-MSNs) inkjet-printed on the active area of a Si micro-cantilever for ammonia detection [289]. Spin coating combines drop casting with high-speed rotation of the substrate. Because of centrifugation, rather uniform and smooth layers can be produced. Electrospray coating [290, 291] and electrospinning [292, 293] use electrical fields to improve the deposition and to produce fiber layers/structures. Jia et al. deposited nanofibers with different specific surface areas on Q-TSMRs from poly(styrene-block-maleic acid) by electrospinning [294] and poly(acrylic acid) (PAA) by electrospray/electrospinning for ammonia detection [295] (see Fig. 13). Electro-netting and electrospinning were employed by Wang et al. to coat TSMRs with two-dimensional layers of pure and NaCl-doped poly(acrylic acid) for trimethylamine detection [296]

**Fig. 14:**
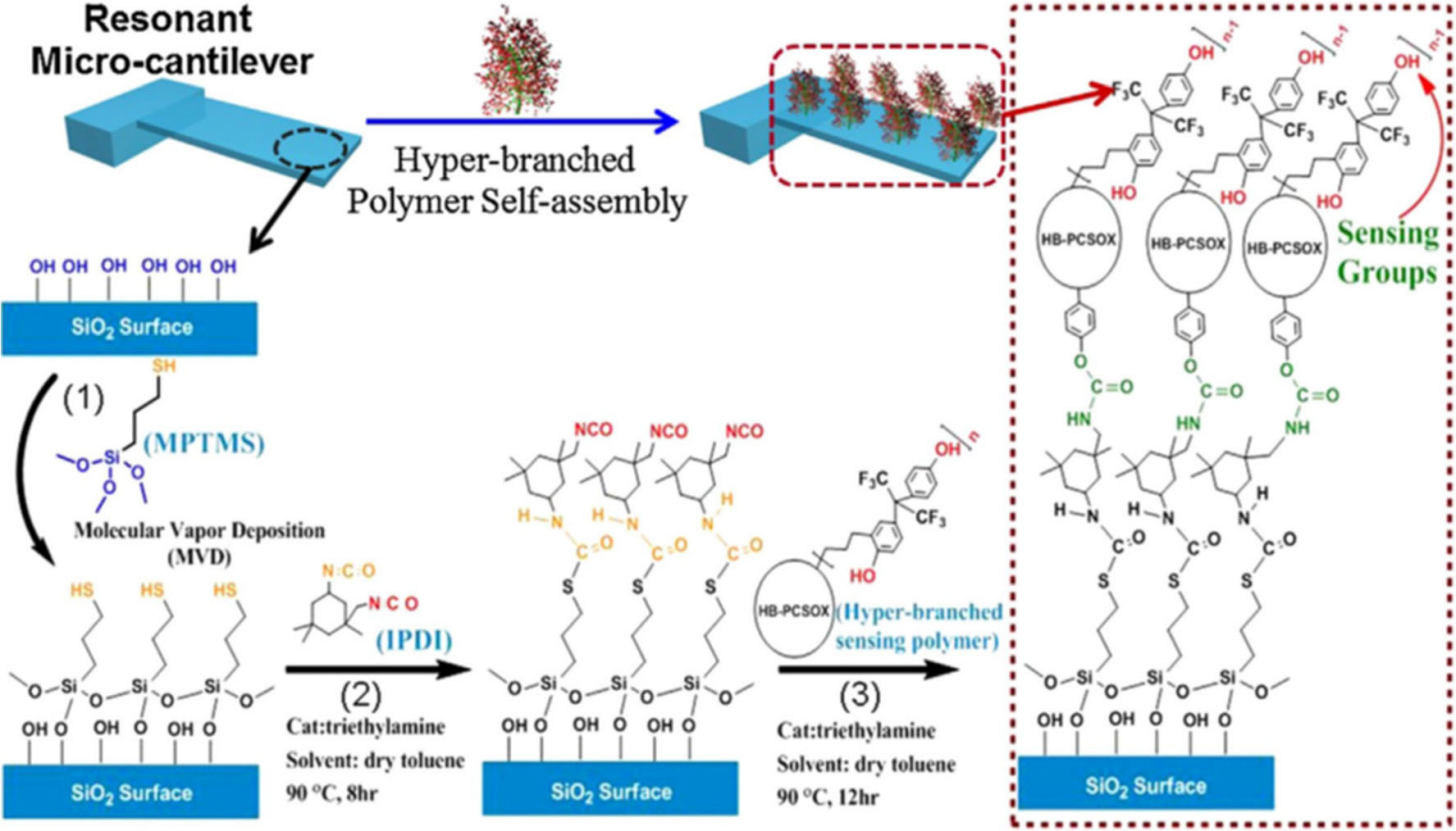
Batch self-assembly of a hyper-branched polymer on a Si micro-cantilever. Reproduced with the kind permission of Elsevier B.V. from reference [303]

**Self-assembly and Langmuir-Blodgett techniques** enable the formation/deposition of molecular monolayers, mainly of organic compounds, through supramolecular processes [297–301]. The methods have also been used for the preparation of gas-sensing monolayers [102]. Xie at al. reported ZnO colloid spheres for alcohol detection prepared via self-assembly [302]. Figure 14 shows the self-assembly of a hyper-branched polymer on a Si micro-cantilever for DMMP detection prepared by Guo et al. [303].

### Chemo-physical methods

The methods included here are numerous [273]: chemical vapor deposition (CVD), plasma-enhanced chemical vapor deposition (PECVD), sputtering (with the variants direct current [304], radiofrequency, reactive and magnetron), thermal evaporation (TE) (with the electron gun/beam variant), atomic layer deposition (ALD) [305–308], molecular beam epitaxy (MBE) [284], pulsed laser vaporization and deposition (PLD) [309], and other laser processing techniques [310, 311]. They are suitable for inorganic thin and very thin layers. Some of them, CVD, PECVD and TE, can also be employed for soft organic materials [312].

### Electrochemical methods

Almost all the MSTs contain metallic electrode structures. Certain types of electrodes are appropriate for electrochemical deposition of sensing materials, or at least for electrochemical polymerization. Because their electrodes cover a compact device area, TSMRs have been mostly used in electrochemically assisted processes. Pristine and Pd-doped ZnO nanorods have been electrochemically grown (in two steps) on quartz TSMRs and used for volatile organic compound (VOCs) detection at room temperature [313].

### Nonconventional deposition approaches

**Fig. 15:**
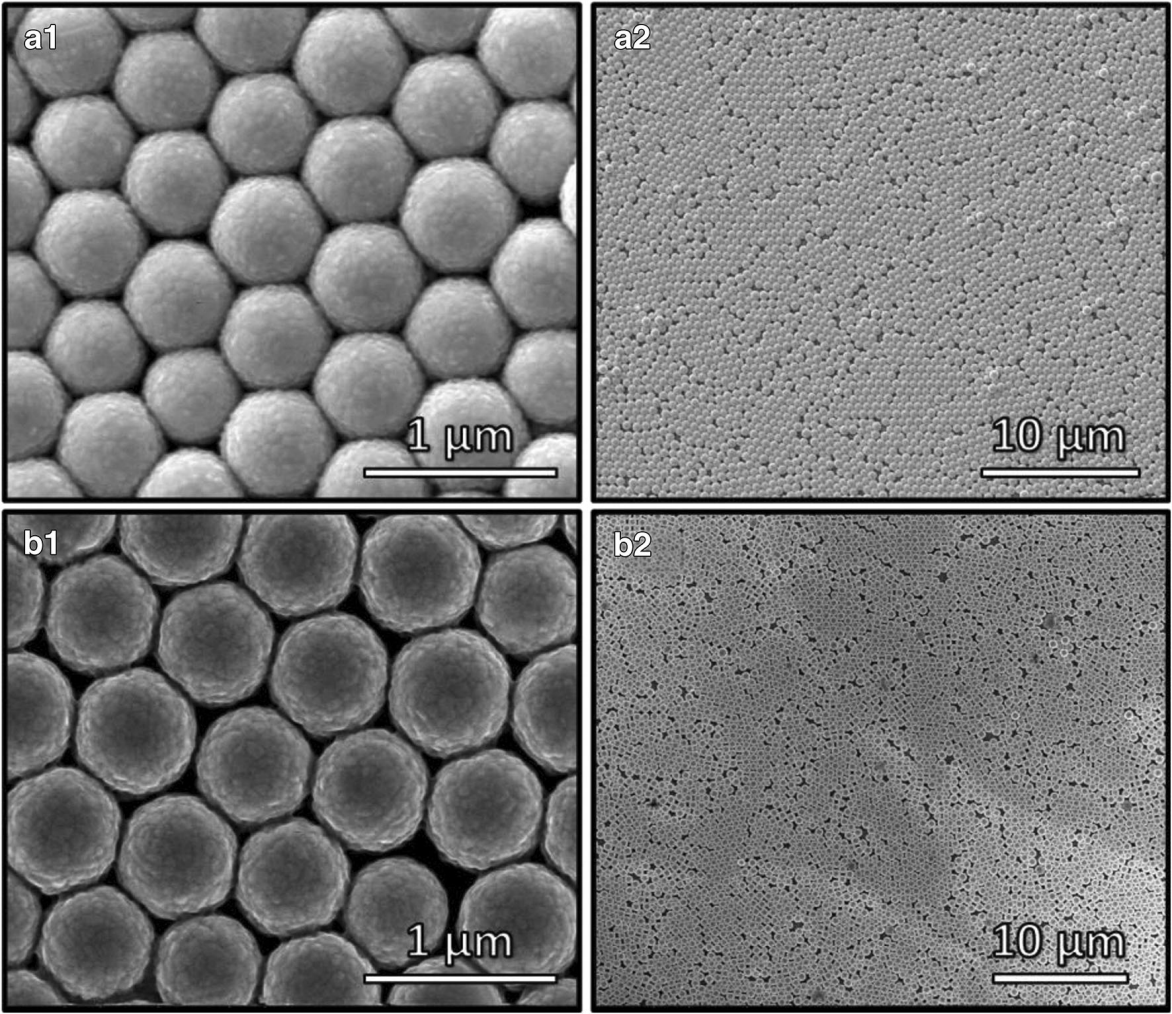
“SEM images representing (a1) close-packed Au-MNM, (a2) surface coverage of Au-MNM on the Ti electrode of QCM transducer, (b1) close-packed Ag-MNM, and (b2) surface coverage of Ag-MNM on the Ti electrode of QCM transducer” (original caption). Reproduced with the kind permission of ACS Publications from reference [315]

Many sensing layers, based on composite materials or stacking layers of dissimilar structure, have also been considered for gas sensing. In order to coat layers with complicated morphologies on MST surfaces, researchers have been pushed to find original deposition procedures. For example, Yan et al. employed a biosynthetic approach to prepare poly(dopamine) nanotubes sensitive to CHOH [314]. Sabri et al. produced poly(styrene) (PS) monodispersed nanosphere monolayers (MNM) on Q-TSMRs by dispersion polymerization, which were then coated by electron beam evaporation with Au and Ag thin films to obtain (Au-MNM) and Ag (Ag-MNM) nanostructures sensitive to Hg vapor [315]. Figure15 displays the SEM images of the films addressed above.

## Evaluating the performance of the gas sensors and sensor arrays^26^

The data provided by the GSs or SAs have very limited utility without appropriate handling. As ascertained in the paragraph “The performance of GGSs” of the first review part, in the case of individual sensors, this mainly involves the determination of the calibration curves (see the paragraph “Univariate and multivariate calibration” of this section), sensitivity, detection limits, response/recovery times and cross-sensitivity (the sensitivity towards gases/vapor other than the targeted one) [316]. On the other hand, SAs acquire large amounts of experimental data which need to be converted into chemical information such as chemical nature and concentration of the components in the gaseous samples. The best way to accomplish this task is to appeal to appropriate chemometric methods, which use mathematical/statistical approaches for data processing and, as such, significantly increase the accuracy of the information provided [316–330]. Kiralj and Ferreira give a nice and unique “etymological, linguistic, and bibliometric” perspective on chemometrics until 2006 [331]. Chemometric software packages such as Unscrambler^®^ (Camo Analytics, https://www.camo.com/unscrambler/), SIMCA^®^ (Umetrics/SartoriusStedim Biotech https://umetrics.com/products/simca) and PLS Toolbox^®^ (EigenVector Research Inc. http://eigenvector.com/software/pls-toolbox/) are available on the market. Not all chemometric methods are equally utilized in the field of GGSs, as obvious from the content of the articles published by different authors. The most relevant ones are briefly presented below and summarized in Table 1.

**Table 1:** Overview of the chemometric approaches employed in gravimetric gas sensing. The first (largest) table section is dedicated to standard methods

SA type	Receptor material	Target analytes	Method	Features	Main outcome	Remarks	Year	Reference
Q-SAW (ST)	Polymers and other organic materials	VOCs (11 compounds)	PCA, CA	12 sensors, 2 PCs	Discrimination, clustering	Historical relevance Hierarchical clusters	1986	[332]
Q-TSMR (AT)	Amide-based stationary phase	Chiral vapors	PCA +PCR	10 sensors, 2 PCs	Discrimination, prediction	Scores & loadings plot Calibration curves	1997	[205]
Q-SAW dual	Polymers and other organic materials	VOCs	PCA, CA	8 sensors, 2 PCs	Discrimination, clustering	Historical relevance Hierarchical clusters	1988	[333]
Q-SAW (ST)	Polymers	CWAs (2 classes)	PCA	3 sensors, 2PCs	Discrimination	EN	2000	[262]
SiN μ-cantilevers	Polymers (PDMS, PEUT)	Toluene, Octane	PCA, PLSR	2 Sensors, 2 PCs	Discrimination, Calibration	PCA scores, PLSR prediction	2001	[227]
Q-TSMR	Pyrrolic macrocycle	Fuji apples headspace VOCs	PCA, PLSR	8 sensors, 2 PCs	Discrimination	EN Libra Nose Classification	2004	[334]
Si-Cantilevers	Polymers	VOCs (3 compounds)	PCA, PLSR	6 sensors, 2 PCs	Discrimination, Calibration	Resonant & static actuation	2006	[253]
Q-SAW (ST)	Polymers	CWAs (4 compounds)	PCA	5 sensors, 2PCs	Discrimination	Good discrimination	2007	[335]
ZnO-SAW	Polymers	VOCs (3 compounds)	PCA, PLSR, PNN	8 sensors. 2PCs	Discrimination, Calibration	PCA scores, PLSR prediction, classification	2007	[265]
Q-TSMR	Conducting polymers of thiophene & derivatives	VOCs (7 compounds) & water	PCA, PLSR	8 sensors, 2 PCs	Discrimination, Calibration	PCA scores, PLSR prediction	2007	[336]
Q-SAW (ST)	Poly(siloxanes), poly(epichlorohydrin)	WASs (2 compounds)	PCA + ANN	3 sensors, 2 PCs	Discrimination/Classification	Pre-concentration stage	2008	[337]
Q-TSMR (AT)	GC stationary phase	Olive oil, headspace	PCA	5 sensors, 2 PCs	Classification	Scores & loadings plots	2010	[338]
Q-TSMR (AT)	Vic-dioximes	VOCs (11 compounds)	PCA	6 Sensors, 2PCs	Discrimination	Score plot	2011	[339]
Q-TSMR (AT)	Porphyrins, polymers	Microorganism activity through release of VOCs	PCA, PLSR, PLS-DA	8 sensors, 3 PCs	Discrimination	EN, Score plot, prediction	2011	[340]
Q-TSMR	rr-P3HT regioregular poly(3-hexyl thiophene)	VOCs	PCA	4 sensors, 2 PCs	Classification	PC2 less than 2% variance	2011	[341]
Lowe-wave Q-SAW (ST)	Polymers	CWAs	PCA	5 sensors, 2 PCs	Discrimination	PC2 1% variance	2012	[229]
Q-TSMR (AT)	Ionic liquids	Food quality, headspace	PCA	7 sensors, 2 PCs	Classification	Cinnamon Samples	2013	[342]
Q-TSMR	Not specified	VOCs	PCA + ANN	1 sensor, 2 PCs	Discrimination	EN Chromatographic column	2011	[266]
Q-TSMR	Porphyrins, peptides	Chocolate quality control (24 VOCs)	PCA, PLS-DA	8 sensors, 3 LVs	Discrimination, classification	EN, PLS-DA score plot	2015	[103]
Q-TSMR	Porphyrins	Bacteria and fungi (13 types)	PCA, PLS-DA	8 sensors, 3 PCs	Discrimination	EN, PCA scores and loadings plots	2016	[104]
Q-TSMR (AT)	GO & pyrrole derivatives	CO, NH_3_, NO_2_	PCA	3 sensors, 2 PCs	Discrimination	Mixtures analyzed	2017	[251]
μ-Cantilevers	Polymer	VOCs (8 compounds)	PCA	8 sensors, 2 PCs	Discrimination	Si and artificial diamond μ-cantilevers	2017	[254]
Q-TSMR (AT)	Short peptides	VOCs (13 compounds)	PCA	5 sensors, 2 PCs	Discrimination	PCA biplots	2017	[57]
Q-TSMR (AT)	CNTs, graphene, CO, PANI	Egg shelf life by released VOCs	PCA, LDA, PLSR	4 sensors,	Discrimination, Calibration	PCA, LDA plots, PLSR prediction	2018	[343]
Q-TSMR (AT)	Hairpin DNA-AuNPs	VOCs (8 compounds)	PCA	7 sensors, 3 PCs	Discrimination	Scores & loadings plots	2019	[58]
Q-TSMR	Metal porphyrins	Headspace of urine samples	PLS-DA, PCA	8 sensors, 2 LVs, 2 PCs	Discrimination	Score plots	2008	[344]
Q-TSMR	Metal porphyrins	Ethanol, toluene, mixture	PLS-DA	7 sensors, 2 LVs	Discrimination	Short time exposure protocol	2011	[345]
Q-TSMR (AT)	IL & conducting polymers	Benzene, formaldehyde, methane, natural gas	LDA	5 sensors	Discrimination	LDA canonical means plot	2011	[244]
Q-TSMR	Ionic liquid	VOCs (18 compounds)	QDA, PCA	1 sensor, 5 PCs for 99% variance	Classification	Virtual array, 7 harmonics Quality factor employed	2015	[236]
Paper cantilevers	Polymers	VOCs (4 compounds)	LDA	8 sensors	Discrimination	LDA canonical score plot	2016	[240]
Q-TSMR (AT)	Ionic liquid	VOCs (8 compounds)	QDA, PCA	1 sensor, PCs for 99% variance	Classification	Virtual array, 5 harmonics Quality factor employed	2017	[237]
Q-TSMR (AT)	Stationary phase	VOCs (4 compounds) + humidity	MLR, PLSR	9 sensors	Multivariate calibration	MLR, PLSR predictions and PLSR calibration	1987	[346]
Q-TSMR	Cyclodextrin, Calix[4]resorcinearenes	m-, p-Xylene, toluene, tetrachloroethylene	PLSR, ANN	4 sensors	Multivariate calibration	PLSR prediction	1999	[228]
Si μ-cantilevers	Polymers (PDMS, PEUT, PCPMS)	*n*-Octane, toluene	PLSR	4 sensors	Multivariate calibration	PLSR prediction	2002	[216]

### The structure and properties of the data provided by SAs

The bare output of the GGSs, usually delivered as electrical signals, is preprocessed by the readout electronics or hardware stages, which provide amplification, noise reduction and the conversion to digital signals. The obtained digital data can undergo simple upgrade (coding) as scaling (division by a value representative for the set, like statistical dispersion, matrix norm, etc.), centering (shifting of the mean to zero) [323], or even smoothening and derivation [347]. More complex data conditioning, such as Fourier transform or wavelet transform, often related to the spectroscopic analytical methods, are less commonly employed in gravimetric gas sensing.

In the SA evaluation approach, the data are obtained from series of *M* gas exposures (events) under the influence of *I* different conditions (factors) {*f*_*i*_}. The individual outputs of the *N* sensors in the array are expressed as features, {*x*_*n*_}. During an event, one has/sets a certain value for each factor and, correspondingly, one obtains a value for each feature. The raw factors have a chemical or physical nature (the concentrations of different gases and vapor in the test mixture, and the temperature, pressure, flow rate of the test mixture, respectively), while the features usually lose their physical identity in the preprocessing stages, becoming abstract numbers. Actually, the factors can also undergo some coding procedures when handled by different chemometric methods. From an algebraic point of view, the factors are independent variables, while the features are dependent ones (for linear algebra concepts please consider appropriate textbooks, like those of Strang [348], or Anton and Rorres [349]). The features belong to an *N*-dimensional space whose coordinates are not linearly independent because of some similarities in the sensor responses [350]. This complicates the estimation of the distance between events in the feature space and the analysis of the possible relationships among them. The SA data are usually organized in a data matrix [351] ***X*** = {*x*_*m*, *n*_}, where *m* ∈ [1, *M*] and *n* ∈ [1, *N*] index the events and the features, respectively. For instance, the matrix element *x*_*m*, *n*_ reflects the output of the sensor number *n* when exposed to the conditions of the event *m*. Each row *m* of the data matrix is a set of features (scores) recorded for a given event, implicitly containing the dependence on the factors acting during that event. It can be regarded as a row matrix (raw score vector) ***x***_*m*_, with the dimension *N*. A column of ***X***, in its turn, is a column vector (column matrix) ***x***_*n*_ in the event space bearing the information about all *M* events corresponding to the feature *n* (that is, all the information outputted by the SA sensor with the number *n*). The larger the variability^27^ among the components of ***x***_*n*_, the more information about the set of events is carried by the feature *n* with respect to the other features. In order to compare the data on different columns of ***X***, they must be normalized (divided by their matrix norm, that is, by the square root of the sum of their squares), because different sensors can give different types/ranges of responses. Care is required when identifying the meaning of the notations in the data reported in the literature. It is possible that the same character has two or more meanings. For instance, “*x*” could be used for independent variables in the experimental design and for dependent variables in PCA (see below). In following, this double use is avoided.

### Experimental design

Before evaluating the GSAs based on the experimentally acquired data, it is good to appropriately conceive the experiments themselves through an “experimental design” [328, 329, 352–354]. Accordingly, one has to identify the factors influencing the responses (features) of the SA in a screening process. The most relevant factors in gas sensing are the target analyte concentration and the influence of the main interferents (like humidity, concentration of the known gases in the sample, temperature and pressure). A good experimental design must provide a choice of factors to be considered and the number of experimental events (including replicates) needed for correct GSA characterization [352, 353]. Response surface methodologies are often employed. They consider that the response *x*_*m*, *n*_ of a given sensor *n*, in the experimental event number *m* (a certain gas exposure for instance), to the selected factors {*f*_*i*_} (gas concentrations for example) can be approximated through a polynomial of low order, containing linear and quadratic terms [323, 355]:

1 $$ {x}_{m,n}\cong {\sum}_{i=1,j=1,{k}_{i,j}=0}^{i=I,j=I,{k}_i+{k}_j\le 2}{c}_{m,n,\kern0.5em i,j}\cdotp {f}_{m,\kern0.5em i}^{k_i}\cdotp {f}_{m,j}^{k_j} $$

where *c*_*m*, *i*, *j*_ are polynomial coefficients, *I* the total number of considered factors, and *i*, *j*, *k*_*i*, *j*_, *m*, *n* indices (natural numbers). In Eq. (1) there are *1* free term, *I* linear terms, *I* quadratic one-variable terms, and $$ \left(\begin{array}{c}I\\ {}2\end{array}\right) $$ quadratic mixed (factor interaction) terms, leading to $$ G=2I+1+\left(\begin{array}{c}I\\ {}2\end{array}\right) $$ coefficients (parameters). A full experiment having *M* events (gas exposures) can be represented in the above approximation by an *M* × *G* design matrix ***D*** (*M* rows and *G* columns). Its elements, *D*_*m*, *n*_, are the monomials ${f}_{m,i}^{k_i}\cdotp {f}_{m,j}^{k_j}$ contained in Eq. (1) for the factor values taken during event *m* ∈ [1, M]^28^. The index *n* ∈ [1, *N*] of the column stands for combinations of the factor indices *i*, *j* in any order (preserved along the design procedure). Inspection of the design matrix or significance tests enables the identification of the relevant factors. Current experimental design approaches include full and fractional factorial designs [323, 352]. The full factorial design includes devised events including all *I* factors, each with different *L* values (levels). Though more accurate, the full factorial design requires a huge number of experiments (*M* = *L*^*I*^). Therefore, a screening procedure at two relevant levels is commonly used in a preliminary step. The fractional factorial design discards, in a systematic manner, part of the events considered in the full factorial one, reducing the dimensionality of the design matrix. Although the design of the SAs with regard to the receptor materials, structure and number of devices has frequently been considered in the literature devoted to GGSs, there are very few attempts to design experiments for the evaluation of SAs. For example, in a paper dating back to 1995, Hierlemann et al. employed Box-Behnken and factorial design to reduce the calibration time of the Q-TSMR-SA and optimize the experiments [226].

### Sensor data evaluation

The data collected during SA evaluation experiments require multivariate analysis because of their statistical nature, large number of factors being considered, and hidden correlations or structure [356, 357]. Dedicated multivariate methods provide the classification of the experimental events (cluster analysis [CA], discriminant analysis [DA], partial least squares discriminant analysis [PLS-DA]) [358–361], reduction of data dimensions without significant loss of information (principal component analysis [PCA] or factor analysis [FA]) [362–366] and multivariate calibration (multiple linear regression [MLR], principal component regression [PCR], partial least squares regression [PLSR]) [367, 368].

*Unsupervised multivariate methods* do not use a priori assumptions about the number, structure or identity of the data, unlike the *supervised methods*, which are based on previous information, typically gathered during training sets of experiments. This prior information can be the number and type of event classes.

#### Unsupervised multivariate data analysis

**Cluster analysis** [358] is a common unsupervised exploratory classification method. Events with similar patterns are included in the same homogeneous class/cluster. Because the patterns appear as points in the space of the features (the sensor outputs in the case of SAs), the clusters are spontaneously formed based on the distances between the events in this space. Generally, Euclidian metrics is employed to calculate the distances:

2 $$ {d}_{i,j}={\left[{\sum}_{n=1}^q{\left({x}_{i,n}-{x}_{j,n}\right)}^2\right]}^{\frac{1}{2}} $$

**Fig. 16:**
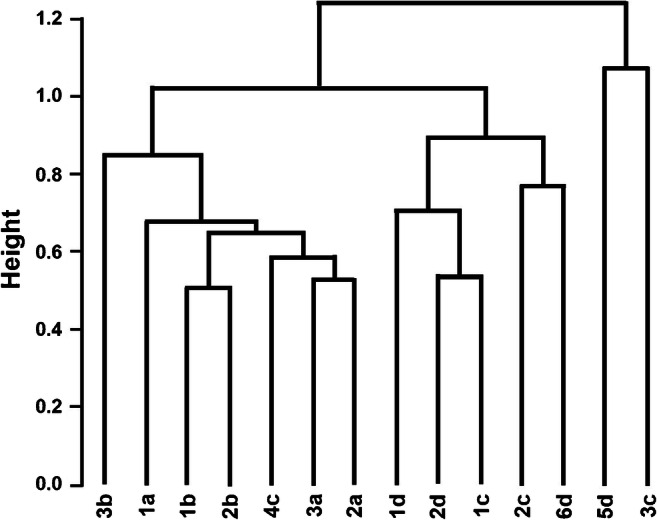
Hierarchical cluster analysis. Dendrogram obtained with 14 fluoroalkyloxy-substituted phthalocyanines. Reproduced with the kind permission of Elsevier B.V. from reference [107]

This supposes orthogonal, linearly independent coordinates, which is mainly not the case, the responses of the sensors being often correlated. Moreover, starting with four features, it is not possible to graphically visualize the clustering process. A frequent solution is the projection of data on orthogonal coordinate axes built up as a linear combination of the dependent variables in the feature space. Further simplification of the procedure may be achieved by using the PCA (see next paragraph and the examples in Table 1). Alternatively, the Mahalanobis distance may be employed [369–371]. Once the problem of correct calculation of distances is worked out, the classes can be hierarchically constructed, starting with one-element clusters and merging, step by step, the closest ones. The plot of the distances between clusters appears as a dendrogram (the plot resembles the branches of a tree), as displayed in Fig. 16. Alongside crisp/hard clusters habitually reported in the literature, fuzzy clustering has also been considered [372]. In this case, the events belong to different classes with different degrees of membership.

**Principal component analysis** (PCA) is the most widely used unsupervised exploratory multivariate method for evaluating the SA output [323, 362–365]. PCA replaces the *N* correlated coordinates in the feature space with a set of *A* ≤ *N* linear combinations of them, which are orthogonal to each other, and hierarchically ordered according to the amount of variability transferred from the original data. Named by Hotelling [373], principal components (PCs) define new abstract features, generally with no experimental meaning.

**Fig. 17:**
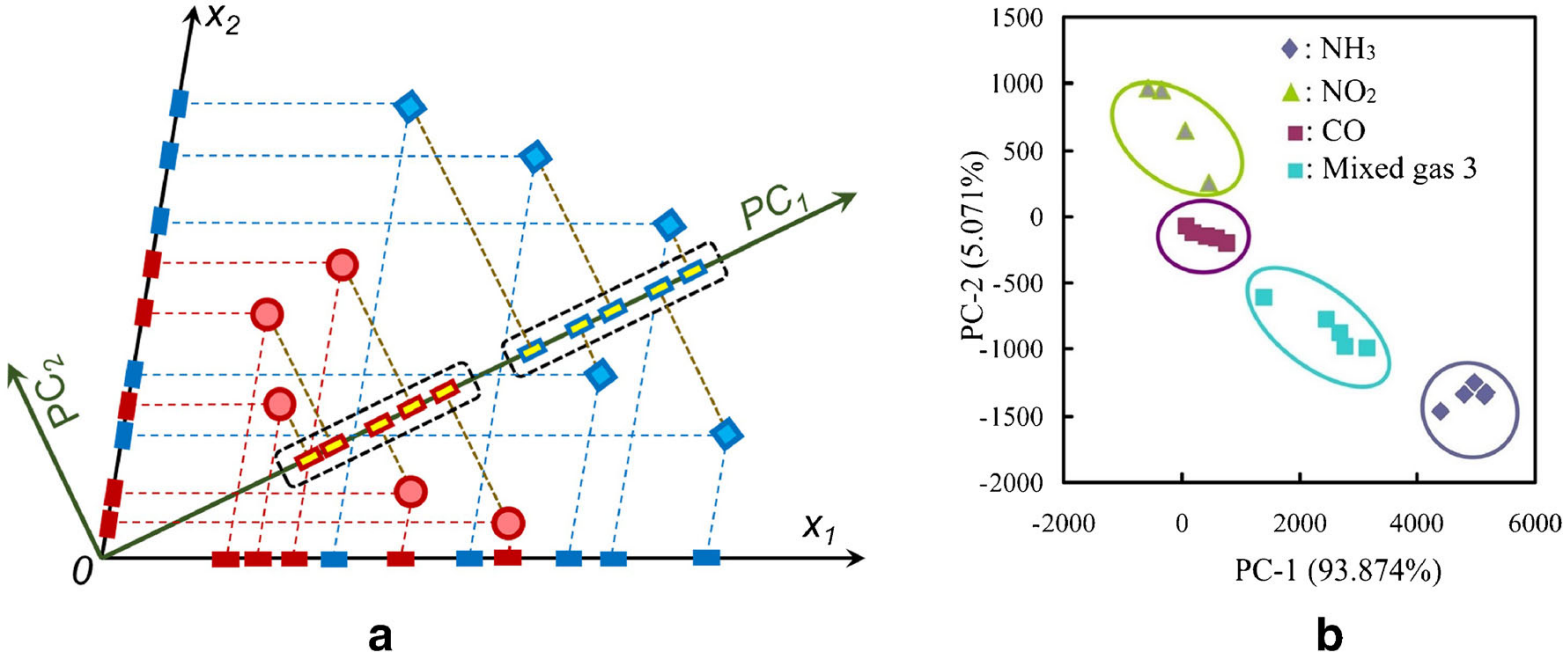
(**a**) Two classes of events in a bidimensional feature space, which appear as mixed on both linearly dependent axes x_1_ and x_2_, are discriminated on the principal components. Moreover, the whole variability is carried by *PC*_1_. The example could express the responses of two gas sensors which have a certain degree of similarity. (**b**) Example of  PCA score plot displaying analyte clustering. Panel (b) was reproduced with the kind permission of Elsevier B.V. from reference [251]

The event producing the original score vector ***x***_*m*_ will also produce an abstract score vector (single row matrix) ***t***_*m*_ = (*t*_*m*, 1_, *t*_*m*, 2_, …*t*_*m*, *α*_, .. *t*_*m*, *A*_) with dimensionality *A* in the new feature space. Formally, each element *t*_*m*, *α*_ of the vector ***t***_*m*_ can be expressed as a linear combination of the elements *x*_*m*, *n*_ of ***x***_*m*_:

3 $$ {t}_{m,\alpha }={\sum}_{n=1}^N{w}_{m,\alpha, n}\cdotp {x}_{m,n} $$

where *w*_*m*, *α*, *n*_ are the combination coefficients (weights of the original features) and *α* ∈ [1, Α] is the index of the *PC*s. In principle, one has to seek all coefficients *w*_*m*, *α*, *n*_ so that all PCs are orthogonal and *PC*1 carries the largest variability, *PC*2 the next largest, and so on (see Fig. 17a). It is worth noting that an arbitrary increase of all *w*_*m*, *α*, *n*_ will result in an arbitrary increase of all *t*_*m*, *α*_, leading to the failure of the procedure. Therefore, it is necessary to additionally request the normalization of the coefficient set {*w*_*m*, *α*, *n*_} for fixed *m* & *α*. In many cases, the first few *PC*s are enough to bear almost all information contained by ***X***, so that the matrix $\hat{\boldsymbol{X}}$ (the hut is a common notation for estimated values), estimated with the help of these *PC*s, differs from the original ***X*** by some small amounts only, the residuals, stored in the matrix of residuals $\boldsymbol{E}=\boldsymbol{X}-\hat{\boldsymbol{X}}$. In this way, PCA ensures a practical reduction of the dimensionality of the feature space. Furthermore, ***X*** can now be factorized using the values of the new features for the given events (the new “scores”) and the “loadings” (components) of the *PC*s in the old feature frame:

4 $$ \boldsymbol{X}={\boldsymbol{TP}}^T+\boldsymbol{E}=\hat{\boldsymbol{X}}+\boldsymbol{E} $$

The matrix ***T*** of scores (actually new sores) is *M* × *A*-dimensional, and that of loadings, ***P***, *N* × *A*-dimensional, so that its transpose ***T***^*T*^ is *A* × *N*-dimensional. PCA, in the limit of selected *PC*s, explains a fraction $\left[{\left\Vert \boldsymbol{X}\right\Vert}^2-{\left\Vert \boldsymbol{E}\right\Vert}^2\right]/{\left\Vert \boldsymbol{X}\right\Vert}^2={\left\Vert \hat{\boldsymbol{X}}\right\Vert}^2/{\left\Vert \boldsymbol{X}\right\Vert}^2$ of the variability contained by ***X*** [365]. The PCA estimations are mainly visualized as scatter plots of scores and loadings either separated or together in a common biplot. In the case of biplots, the graphical separation of the events might not be the real one due to the different units or scaling factors being used (commonly, the loadings are scaled to 1 while the scores bear the variability of the corresponding PC). There are several available approaches to find the PCs, including nonlinear iterative partial least squares (NIPALS) [362, 374], singular value decomposition (SVD) [349] and covariance matrix. The authors reporting on GGSs mainly use PCA to check event clustering and present their results as score plots. More than half of the papers listed in Table 1 include PCA approaches. A graphical example is reproduced in Fig. 17b.

**Factor analysis** (FA), despite its formal resemblance to PCA, adopts a different point of view [366, 375–377]. Instead of seeking a linear combination of dependent variables to obtain new ones (the *PC*s), orthogonal and ordered with respect to the carried variability (as PCA does), FA looks for a set of *m* independent (random) variables (common factors—) whose linear combinations render the dependent variables up to a residual variable (specific factor)^29^*e*:5

The coefficients *a*_*nm*_ are the factor loadings of the variable *x*_*n*_ on the *m*^th^ factor. By using the name “common factor”, one intends to point out the influence of such independent variable on all dependent variables. Equation (5) is apparently identical to the linear part of Eq. (1), but apparently only because the factors *f*_*i*_ in Eq. (1) are real factors, with physicochemical relevance, while the  factors of FA lack this character, being formal entities. In matrix form, Eq. (5) becomes $\boldsymbol{x}=\boldsymbol{A}\mathbf{\mathcal{F}}+\boldsymbol{e}$ and shows that FA is a model (regression—see next paragraph)-based approach, while PCA is not (in fact, not necessarily).

#### Supervised multivariate data analysis

**Discriminant analysis** (DA) [359] attempts to assign events (objects) whose features are categorical variables to predefined classes. The factors considered by DA are continuous variables. The method seeks discriminant functions of independent variables simultaneously, leading to the largest separation between classes and the smallest ones inside each class. Commonly used approaches include linear discriminant functions (LDA, initially introduced by Fisher for two classes only [378]^30^), soft independent modelling of class analogy (SIMCA), [379, 380] and *k*-means clustering [381]. Nice LDA discrimination of methanol, ethanol, acetone and tetrahydrofuran with a very simple paper cantilever SA was reported by Fraiwan et al. [240]. By using quadratic DA (QDA) and a virtual Q-TSMR-SA, Speller et al. obtained good separation of a large number of VOCs [236].

#### Univariate and multivariate calibration

Calibration entails establishing a connection between two sets (blocks) of variables. For SA data, they are the sensor responses {*x*_*m*, *n*_} = ***X***, one for each sensor *n* in the measurement number *m*, and the factors {*f*_*m*, *i*_} = ***F***, mainly the concentrations {*c*_*m*, *i*_} = ***C*** of different *I* gases in the test mixture employed during measurement *m*. The number *M* of calibration tests must exceed the number *N* of the sensors in the SA in order to allow a nontrivial solution for the approach (*M* ≥ *N*).

**Univariate calibration** relates one feature (sensor response) to one factor (target gas concentration). However, it does not involve only one event. To reduce the effect of experimental errors affecting both feature and factor, and to identify the type of relationship between them, a series of measurements (with replicates) is performed. In this way, one obtains a column vector of features ***x***, containing the responses of the sensor to the corresponding concentrations of the analyte (also written as a column vector). In many cases, the relationship sought appears to be a linear regression ***x*** = *s* ∙ ***c + e***_*x*_ which, through simple matrix algebra, results in [323]:

6 $$ s\cong \left({\sum}_{m=1}^M{c}_m\cdotp {x}_m\right)/\left({\sum}_{m=1}^M{\left({x}_m\right)}^2\right) $$

Here, the scalar *s* is the sensor sensitivity and ***e***_*x*_ the error-vector of responses. The index 1 of the first rank column matrices (expressing only one feature) was omitted. Because the task of the sensors is to provide the value of the factor (concentration) when the feature (response) is known, one needs to consider an **inverse calibration** ***c*** = *b* ∙ ***x + e***_***c***_, where ***e***_*c*_ is the error-vector of concentrations. In the limit of experimental errors, *b* is the inverse of the sensitivity, and its estimation is given by:

7 $$ b\cong \left({\sum}_{m=1}^M{c}_m\cdotp {x}_m\right)/\left({\sum}_{m=1}^M{\left({x}_m\right)}^2\right) $$

If the sensor has a nonzero baseline (the sensor response in the absence of the analyte is not zero, but ***x***_**0**_), then one has to subtract the baseline vector ***x***_0_ from the vector of the responses ***x*** in the calibration equation, so that ***c*** = *b* ∙ (***x − x***_0_) ***+ e***_*c*_. The vector ***x***_0_ contains the actual values of the baseline during the experiments *m* ∈ [1, *M*].

**Multivariate calibration** broadens the univariate calibration towards the case of multiple factors and features (several analytes and several sensors, possible in a SA)

**Multiple linear regression** (MLR) is a generalization of the univariate linear regression and is expressed by the matrix extension of the corresponding univariate equations^31^:

$\boldsymbol{C}=\boldsymbol{X}\cdotp \boldsymbol{B}+{\boldsymbol{E}}_{\boldsymbol{C}}\kern0.5em \boldsymbol{\to}\kern1em \hat{\boldsymbol{B}}={\left({\boldsymbol{X}}^{\boldsymbol{T}}\boldsymbol{X}\right)}^{-\mathbf{1}}{\boldsymbol{X}}^{\boldsymbol{T}}\boldsymbol{C}$. With the matrix $\hat{\boldsymbol{B}}$ obtained in the calibration procedure, one can predict (estimate) the unknown concentrations for a new sample containing the same analytes: ${\hat{\boldsymbol{C}}}_{\boldsymbol{new}}={\boldsymbol{X}}_{\boldsymbol{new}}\cdotp \hat{\boldsymbol{B}}$. The method has a non-negligible disadvantage. It requires knowledge of the responses and concentrations for all relevant compounds in the training sample.

**Principal component regression** (PCR) was developed to directly relate the factors (concentrations of the analytes) to the scores on the first PCs, through an abstract matrix ***R***, avoiding the large amount of data and computations demanded by MLR. Thus:

8 $$ \boldsymbol{C}=\boldsymbol{T}\cdotp \boldsymbol{R}+{\boldsymbol{E}}_{\boldsymbol{C}}\kern0.5em \to \kern1em \hat{\boldsymbol{R}}={\left({\boldsymbol{X}}^{\boldsymbol{T}}\boldsymbol{X}\right)}^{-\mathbf{1}}{\boldsymbol{X}}^{\boldsymbol{T}}\boldsymbol{C}\kern1.25em \&\kern1.25em {\hat{\boldsymbol{C}}}_{\boldsymbol{new}}={\boldsymbol{X}}_{\boldsymbol{new}}\cdotp \hat{\boldsymbol{R}}. $$

(The index “new” addresses the values in a new experiment, either validation or prediction).

**Fig. 18:**
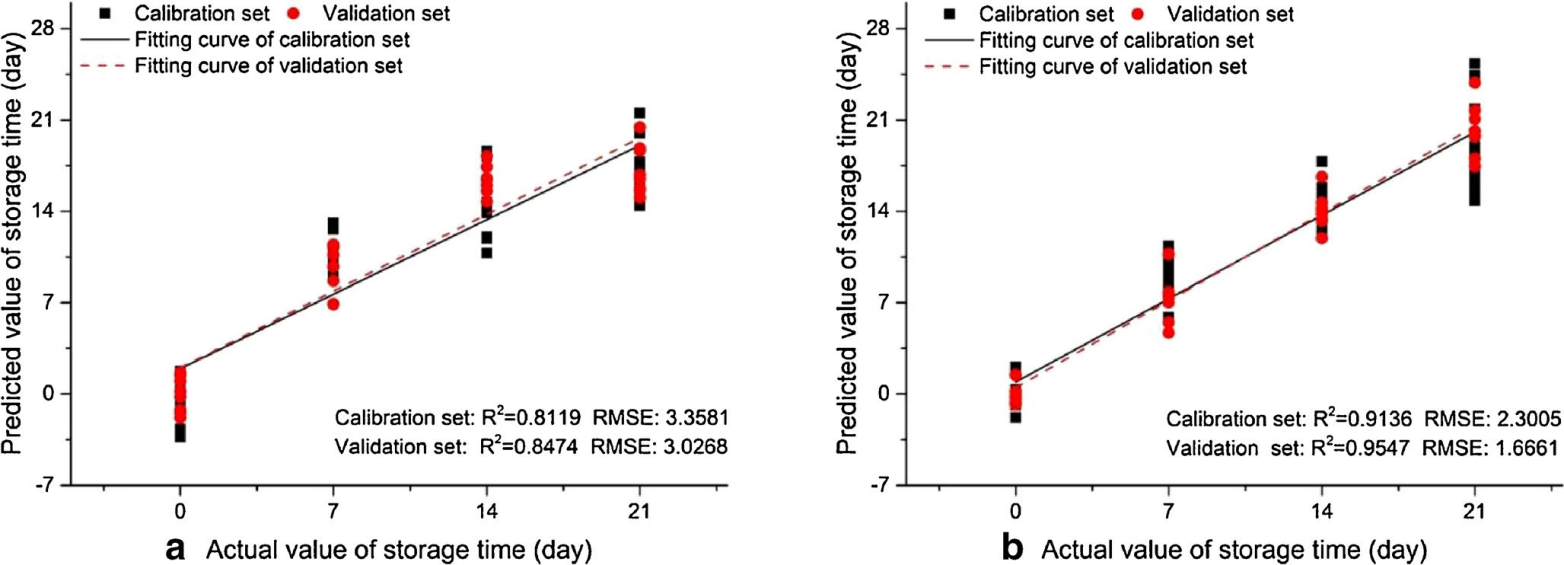
PLSR prediction of the on-shelf life of eggs. (**a**) PLSR results for original data, (**b**) PLSR results after an intermediate KPCA processing. KPCA (Consider references [384] and [385] for additional information on KPCA. See also the section dedicated to support vector machines below.) stands for kernel PCA. Reproduced with the kind permission of Elsevier B.V. from reference [343]

**Partial least squares regression** (PLSR)^32^ [346, 367, 382, 383] uses two matrix decompositions (often referred to as models), one for the features, as in PCA, and another for the factors (concentrations in the gas sensor case):

9 $$ \boldsymbol{X}={\boldsymbol{TP}}^T+{\boldsymbol{E}}_{\boldsymbol{X}}\kern2em \&\kern2em \boldsymbol{C}={\boldsymbol{UQ}}^T+{\boldsymbol{E}}_{\boldsymbol{C}} $$

where ***U*** and ***Q*** play roles analogous to scores and loadings, respectively, for the data block ***C***. The presence of the matrices of residuals for features and factors in Eq. (9) indicates that a reduction of dimensionality is performed, so that some unexplained variability remained. If the sensor responses and concentrations are directly proportional, then one would expect a linear relation ***u***_*i*_ ***= b***_*i*_***t***_*i*_ between the elements of ***U*** and ***T***. ***u***_*i*_ and ***t***_*i*_ are column vectors of ***U*** and ***T***, while ***b***_*i*_ is the equivalent of the regression coefficients in MLR or PCR [323, 367]. The best prediction $\hat{\boldsymbol{C}}$ is obtained when the matrix of the residuals ${\boldsymbol{E}}_{\boldsymbol{C}}=\boldsymbol{C}-{\boldsymbol{UQ}}^T=\boldsymbol{C}-\hat{\boldsymbol{C}}$ reaches its minimum for the given events. PLSR is well suited for overdetermined data sets (more events than features). PLSR is rather widespread in the GGS community. For instance, the application of PLSR to the shelf life of eggs gives good results, as Fig. 18 shows.

**Partial least squares discriminant analysis** (PLS-DA) is an algorithm (certain authors consider it a method) used to perform supervised classification adapting PLSR to clustering problems [360, 361, 386].

### Artificial intelligence in chemometrics

Artificial intelligence (AI) has emerged from the increasing capability of computers to perform complex tasks, some of them already listed/foreseen in the birth document of the concept [387]. “A historical survey of algorithms and hardware architectures for neural-inspired and neuromorphic computing applications” was published not too long ago [388]. The impact of AI on chemometrics is constantly growing [325, 389, 390], with the most widely employed being the artificial neural networks (ANN) [391–397], support vector machines (SVM) [398–404], genetic algorithms (GA) [405, 406] and expert systems (ES) [404, 407–409]. The use of AI in conjunction with GGSs is summarized in Table 2.

**Table 2:** Short overview of the use of artificial intelligence to solve the chemometric problems of gravimetric gas sensors. The final two rows are dedicated to SVM

SA type	Receptor material	Target analytes	Features	AI Type	Main outcome	Remarks	Year	Reference
Q-TSMR	Organic materials	VOCs	3 layers	BP – ANN	Pattern recognition	SA	1991	[410]
Q-TSMR	Modified poly(siloxanes)	VOCs	Box-Behnken & factorial design	FFN, PLSR	Long term stability	SA	1995	[226]
Q-TSMR	Poly(siloxanes)	Toluene octane mixtures	Levenberg-Marquardt algorithm	FFN	Dynamical models for nonlinear gas sensors	SA	1996	[411]
Q-TSMR	Polymers	VOCs	Kohonen	Kohonen ANN	Mapping chemical functionality	SA	1999	[412]
Q-TSMR (AT)	Modified cyclodextrins	Limonene enantiomers	Not specified	ANN	R-S discrimination, prediction	SA	2001	[210]
SAW	Poly(isobutylene)	VOCs (biological relevance)	Chromatographic column + SAW	BP – ANN	Classification / Lung cancer recognition	EN	2005	[239]
μ-Cantilevers	Polymers, macrocycles	VOCs (EtOH, MeOH, DCM, TCE)	Static bending, optical readout	BP – ANN	Analyte & concentration	SA	2006	[230]
Q-TSMR	Phthalocyanines	Sevoflurane	Levenberg-Marquardt algorithm	FFN	Linear MC	EN	2007	[413]
ZnO-SAW	Polymers	VOCs	PCA – PNN	PNN	Classification & calibration	SA	2007	[265]
Q-SAW (ST)	Poly(siloxanes), poly(epichlorohydrin)	Warfare agent simulants	PCA + ANN	BP – ANN	Classification	EN	2008	[337]
Q-TSMR	Ionic liquids	VOCs	4 classes output	BP – ANN	Classification	SA	2009	[241]
Q-TSMR	Phthalocyanines	Toluene, humidity	PCA + Levenberg–Marquardt algorithm	FFN	Calibration, Humidity rejection	SA	2010	[376]
Q-Lowe SAW	Polymers	CWA	PCA – PNN	PNN	Classification	SA	2012	[229]
Q-TSMR	Not specified	VOCs	Chromatographic column	BP – ANN	Classification	EN	2011	[266]
Q-TSMR	PDOT:PSS poly(3,4-ethylene dioxy-thiophene):poly(styrene sulfonate)	Humidity & pressure	Frequency shift and motional resistance employed	BP – ANN	Calibration	Single sensor	2016	[414]
Q-TSMR	PAA – MIPs	Organic acids in body odors	PCA + SVM radial basis kernel function	SVM (e1071)	Classification	SA	2014	[248]
Q-TSMR	PAA – MIPs	Aldehydes in body odors	PCA + SVM radial basis kernel function	SVM (e1071)	Classification	SA	2015	[247]

**Fig. 19:**
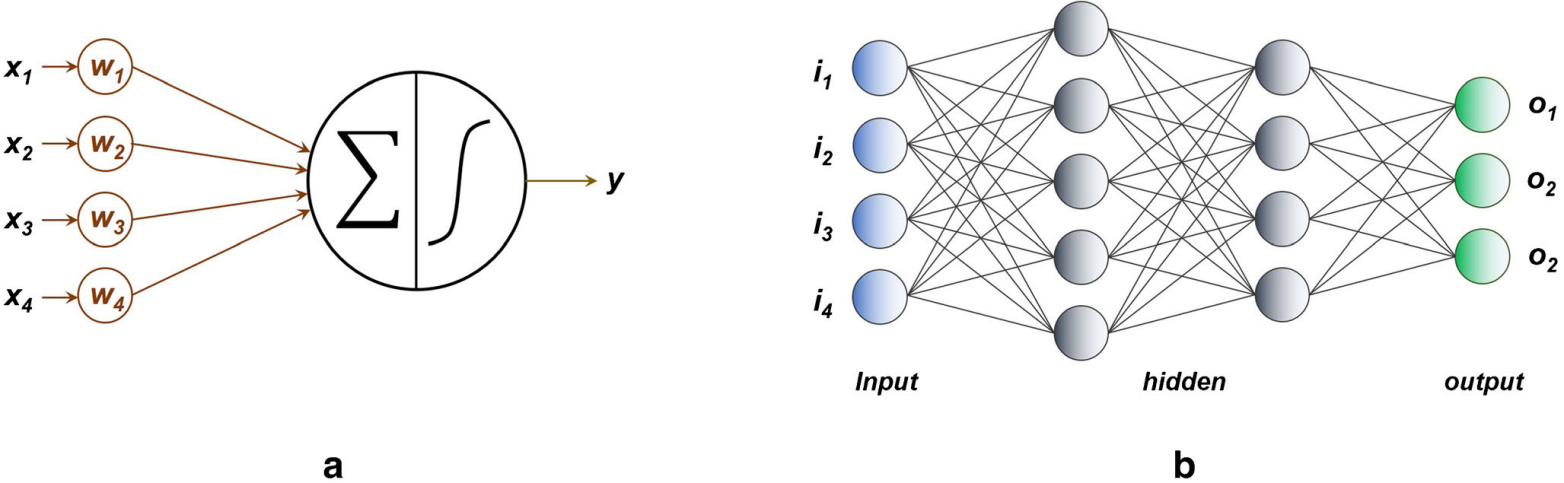
(**a**) The conventional sketch of an artificial neuron. The inputs {*x*_*n*_} (vector ***x***) are adjusted with the weights {*w*_*n*_} (vector ***w***) and added with the block ***Σ***. The result is squashed with the transfer function ***∫*** and delivered as output *y* (a scalar in the sketch). (**b**) Simple feed-forward ANN with four layers of which two are hidden. The data flow from left to right in the sketch

**Artificial neural networks** (ANN) are computing algorithms loosely mimicking the human brain and neural system. The origins of ANN are related to the relevant papers of McCulloch and Pitts and of Hebb, which provide a logical/mathematical understanding of the nervous [415] and sensorial [416] activity, respectively bridges “the gap between neurophysiology and psychology”, addressing synaptic plasticity and associative learning [417]. The building block of the ANN is the artificial neuron (AN) as a conceptual counterpart of its biological model (for the anatomy and physiology of biological neurons and neural systems, please refer to the literature [418, 419]). The first AN implementation (the logic threshold unit [LTU]) by McCulloch and Pitts has several Boolean inputs and a single digit output, which is 1 if an internally threshold is exceeded by the sum of all inputs and 0 otherwise. The “perceptron”^33^ proposed by Rosenblatt [420] and refined by Minsky and Papert [421] increased the functional capability of LTU. It allows for weighted inputs, expressing different degrees of importance, but retains the same two-level output triggered by a discrete decision function. The utility of separated neurons is reduced, and their applicative power comes out from interconnections in ANN [388, 391, 396, 422]. The network approach requires new or improved/adapted features of ANs. Figure 19a illustrates a widely used AN structure.

ANNs are built following two main architectures: feed-forward networks (FFN) and recurrent networks (RN). FFNs (see Fig. 19b) are constructed from two or more layers of ANs. The input layer is supplied with the data to be processed (a vector ***x***_*m*_ expressing the event *m* in the *N*-dimensional feature space of a SA, for example). The output of each AN from one layer is sent to all inputs of the ANs in the next layer up to the output layer, which delivers an output variable ***y***_*m*_ (here symbolized as a vector, which could indicate the class to which the event *m* belongs, if a classification problem is to be solved by the ANN). To fit the ANN to its task, an adaptive control is realized, usually by tuning the weights at each node. It has been shown that the functionality of ANNs can be increased significantly when recurrent architecture is chosen, that is, feedback loops are considered [422–425]. The most commonly employed feedback type is the “back-propagation” (BP). It involves two operating steps. In the first step the ANN accomplishes its task in a feed-forward mode, with all weights fixed. Afterwards, it estimates the error it made, providing error parameter(s). In the next step the weights are actualized to correct/minimize the error. An unsuitable choice of starting weights and of their tuning procedure might result in unstable ANN operation and eventually oscillating/divergent output (this is a common issue in the systems controlled by feedback) [396, 426]. The ANNs are able to learn, that is, to adapt their free parameters in such a way as to be able to perform certain tasks [427]. In supervised learning, a training set of known events is run by the ANN, which receives for each input vector the true output value(s). Based on these data, the algorithm adapts itself to provide maximal accuracy during a given number of training cycles. If it fails, it must be improved and checked again. In the case of unsupervised learning, no “true” output data are available, so the training is successful when producing consistent output, like good classification patterns. Zupan and Gasteiger proposed a short and intuitive approach to this topic [392]. An exhaustive overview on “deep learning in neural networks” up to 2015 was provided by Schmidhuber [428]. Very frequently researchers in the field of GGSs use standard chemometric methods, mainly PCA, together with ANN (see Table 2).

**Fig. 20:**
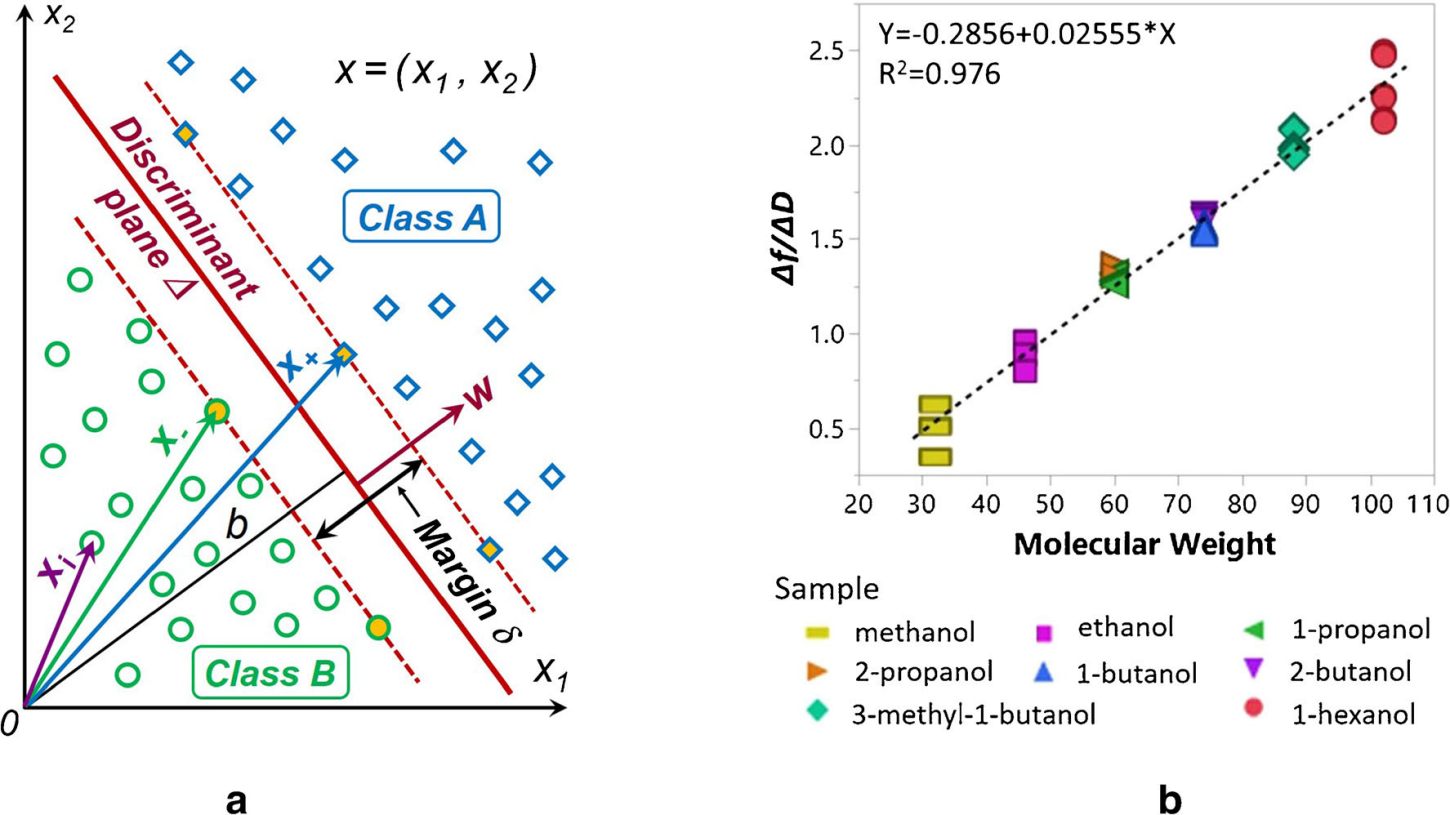
(**a**) Hard-margin SVM: two-dimensional sketch. The events are displayed as ***x*–** vectors with two components *x*_1_ and *x*_2_, and are classified as class A (green open circles) and B (blue open rhombs), respectively. The classes are disjoint and best separated by the plane ***∆***. The margin events (orange filled symbols) are specified by the support vectors (from which only ***x***_+_ and ***x***_−_ are depicted in blue and green, respectively). The vector ***w*** (perpendicular to ***∆***) mathematically describes the discriminant plane direction which lies at the distance *b* from the origin. The margin *δ* is measured along ***w***. ***x***_*i*_ is a generic event, as used in SVM formalism. (**b**) Analyte discrimination through the proportionality of the TSMR output parameter ratio (frequency shift over dissipation factor) and the molar mass. Panel (b) reproduced with the kind permission of Elsevier B.V. from reference [237]

**Support vector machines** (SVM) are supervised algorithms employed for non-probabilistic classification and regressions. SVM started from linear binary classification [398] (analogous to LDA) and evolved towards elaborate nonlinear algorithms. Initially a “training algorithm for optimal margin classifiers” [429] was conceived, whose task was to find the hyperplane in the feature space that best discriminated two classes of events^34^ (see Fig. 20a). Mathematically, this means that the sum of distances from this hyperplane to the closest event(s) of each class—the margin—has to be maximized [399, 430]. The vectors in the feature space [431] that specify the position of the margin events—the “support vectors”—play a special role in the approach, determining the location of the discriminant hyperplane [399, 430]. The “hard margin” addressed above can seldom be obtained because of the experimental noise or incomplete variability explanation. Therefore, Cortes and Vapnik relaxed the separation condition at the expense of classification accuracy, building up a soft margin classifier [432]. In this case, the set of the support vectors is completed by additional, non-margin, ones. The maximum margin hyperplane approach can also be extended for nonlinear classifiers, as emerged from the paper by Boser et al. [429]. First, one should note that the margin optimization problem for both hard and soft cases involves the event vectors as direct products 〈***x***_*i*_, ***x***_*j*_〉 only. Therefore, if one maps the event vectors from the *N*-dimensional feature space into a higher-dimensional space (see footnote 34) $\boldsymbol{x}\overset{\varphi }{\to}\boldsymbol{\varphi} \left(\boldsymbol{x}\right)$ the optimization problem will “move” into the new space, but will still be based on the direct products of the type 〈***φ***(***x***_*i*_), ***φ***(***x***_*j*_)〉 only. Provided the function *φ* is suitably chosen, a linear discriminant might be found in the new space. However, instead of looking for convenient mapping functions *φ*, it is much easier to seek “kernel functions” *k* of two event variables that straightforwardly map the pair (***x***_*i*_, ***x***_*j*_) to direct products 〈***φ***(***x***_*i*_), ***φ***(***x***_*j*_)〉. It remains now to replace the direct products 〈***φ***(***x***_*i*_), ***φ***(***x***_*j*_)〉 in the optimization problem with the corresponding kernel function *k*(***x***_*i*_, ***x***_*j*_) and to train the algorithm. Detailed explanations and examples are given by Burges [399] and Luts et al. [403].

Looking to Fig. 20a, one could think that the vector ***w*** indicating the direction of the discriminating plane ***∆*** would be the same as that of *PC*_1_ in a PCA approach to the data set. That is usually not the case, because *PC*_1_ is chosen to maximize the explained variability of the whole data set, while ***w*** is used to maximize the margin, which depends on only a few data points, pointed to by the support vectors. Generally, it worth noting that all AI methods involve adaptive and autonomous algorithms, while “traditional” multivariate data analysis is based on stated numerical procedures.

An example of combining kernel functions with PCA towards KPCA and further use of PLSR is given in Fig. 18 above.

The genetic algorithms and expert systems are not popular in the field of GGS.

A general approach to chemometric and AI topics is given in the *Handbook of Machine Olfaction* [433].

### Unconventional theoretical and numerical approaches to sensor data analysis

**Fig. 21:**
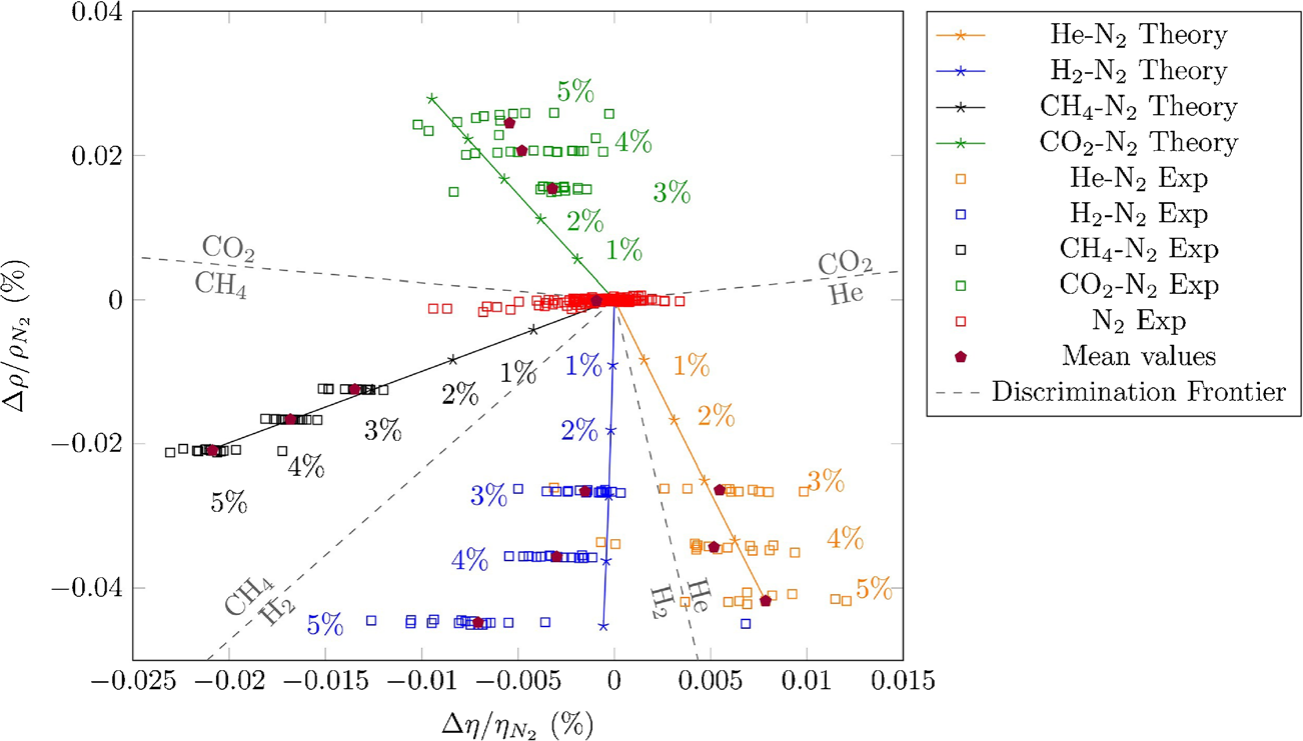
Physical detection and discrimination of gases with uncoated micro-cantilevers. Reproduced with the kind permission of Elsevier B.V. from reference [439]

Unconventional theoretical and numerical approaches to sensor data analysis are used by the researchers in their attempt to better understand, simulate and fit the experimental information. Davide et al. propose a block-structured mathematical model for the GGS based on TSMRs [434, 435]. From the adsorption isotherms of formaldehyde on bio-inspired poly(dopamine) Yan et al. derive the standard enthalpy of sorption which well correlates with the DFT simulations [314]. Sharma et al. used a relatively simple approach, based on the time dependence of the sensor response, to obtain analyte recognition [436]. Accordingly, the maxima of the sensor responses for an exposure to analyte concentration pulses (named “dynamic headspace technique” by the authors) are shifted in time, somehow as by a chromatographic column, improving the recognition procedure. This provided a better way to evaluate the amount of linalool, which is a relevant VOC for traditional tea flavor. Regmi et al. observed that the ratio of the change in resonance frequency to the change in motional resistance (the resistance in the equivalent circuit of the TSMRs which corresponds to the mechanical damping of the crystal) was concentration-independent but proportional to the molecular weight of the absorbed analyte [178]. This proportionality allowed the identification of the VOCs for which the device was sensitive according to their mass. The procedure was upgraded through the use of virtual sensor arrays [237] (see Fig. 20b). A molecular dynamics approach was successfully used by Khanniche et al. to fit the output of a TSMR covered with methylated mesoporous silica in interaction with vapors of the nitroaromatic compounds 2,4-dinitrotoluene (2,4-DNT) and trinitrotoluene (TNT) [437]. A humidity correction was performed so that the simulations agreed with the experimental data. Based on the information from the gravimetric responses of TSMRs coated with calixarene films and colorimetric analysis, Kostyukevych et al. proposed a nanostructure model of the material in which almost spherical stochastically distributed nanocavities enabled the gas (ethanol) adsorption [99]. Nimsuk and Nakamoto proved the utility of an original and unexpected approach to the classification of apple and muscat flavors with variable concentrations [438]. They applied the short-time Fourier transform to the SA data, which indicated different time constants of the sensors for different flavors. In the final step, a linear vector quantization (LVQ) ANN was used for successful discrimination of the two flavors. Iglesias et al. used blank Si micro-cantilevers to discriminate and quantify H_2_, CO, CO_2_, CH_4_, He and O_2_ diluted in nitrogen in percent ranges [439]. The physical sensing principle is based on the differences in the density and viscosity of these gases, which modify the resonant frequency and quality factor of the device upon environment composition. In the space of the relative density and viscosity (referred to nitrogen), each analyte appears on a certain straight line, whose angle with the carrier gas (N_2_) line indicates the analyte type, while the distance from the origin is proportional to the concentration (see Fig. 21).

## Targeted analytes and application

The spectrum of GGS applications reported in the literature is very large and overlaps that of other sensor types, even that of established ones such as the capacitive for humidity or chemoresistive for reducing gases. A full characterization of the sensors/SAs is not always performed or provided in the papers, probably because of the effort and time expense involved. The authors usually indicate the target analytes for their sensors and warn of possible interference, contamination or adverse action from other gases/vapors. In the case of critical requirements, researchers also prove the suitability of their devices for the given task. The review includes two paragraphs below dedicated to GGS and SA applications, one addressing targeted analytes and the other the expected sensor use. Subsequent rough classifications are also given. Almost all papers addressed in this section are listed in the Annex table.

### Targeted analytes

#### Relevance of gaseous analytes detected with GGSs and legal/institutional regulations concerning their concentration limits

There are several kinds of analytes appropriate for detection with GGSs. Some have injurious biological effects (are toxic, irritants, carcinogenic, mutagenic, reprotoxic) [440], while others are flammable or explosive [441]. There are many others which are chemically inoffensive but their concentration in the atmosphere or in closed spaces is relevant for industry, agriculture and other human activities. The physiological and clinical consequences of harmful gases/vapors, starting with cutaneous contact and inhalation, and following the whole metabolic process, are the subject of numerous articles [442, 443] and textbooks [440, 444] (also for veterinary use [445]). Efforts are spent in identifying hazards as sources of potential damage/harm to individuals and optimizing their assessment [446–449]. The exposure of persons, animals and the environment to harmful gases/vapors is legally regulated worldwide. Accordingly, several concentration limits are established for noxious agents which, according to medical investigations, would be still acceptable without adverse effects. They differ, more or less, from country to country, and are included in the documentation of the authorized national, European and international institutions such as the Occupational Safety and Health Administration (OSHA) and National Institute for Occupational Safety and Health (NIOSH) in the United States (USA), the European Chemicals Agency (ECHA) of the European Union (EU) through its Registration, Evaluation, Authorisation and Restriction of Chemicals (REACH) regulation, the German Institute for Work Safety (Institut für Arbeitsschutz [IFA]), British Health and Safety Laboratory (HSL), Japanese National Institute of Occupational Safety and Health (JNIOSH), Chinese Standard Committee of Public Health (CSCPH) and Council for Occupational Health and Safety of South Africa (SACOH). Through the implementation of the Globally Harmonized System of Classification and Labelling of Chemicals (GHS), an international agreement managed by the United Nations, the national/regional safety norms should converge towards joint regulations, simplifying sensor evaluation and certification procedures [450–452]. In the case of gases, both the exposure dose and maximal concentration are relevant for the evaluation of professional hazards. At present, the US standards are the most commonly used in the literature. The permissible exposure limit (PEL) is the legal exposure limit for employees in the USA. PEL values are given by the Code of Federal Regulations (CFR), Part 1910–Occupational Safety and Health Standards, Section 1910.1000, Air contaminants. The threshold limit value (TLV) of a toxic substance is the concentration at which a person can be exposed at work every day for whole life. TLV is a reserved term of the American Conference of Governmental Industrial Hygienists (ACGIH) and has no regulatory nature. It can be evaluated for 8-hour working days, 5 working days a week, as the threshold limit value–time-weighted average (TLV-TWA). The short-term exposure limit (TLV-STEL) indicates the concentration limit for 15 minutes of exposure, while the ceiling limit (TLV-C) is the upper concentration for any exposure which still does not affect the health. The recommended exposure limit (REL) and immediately dangerous to life and health (IDLH) are reserved terms from NIOSH as guidelines of this institution. In the framework of EU–REACH, two concentration limits have been defined: the derived no-effect level (DNEL) is the “exposure level above which humans should not be exposed”, and the predicted no-effect concentration (PNEC) is the concentration for which “no adverse effects” are expected. The Arbeitsplatzgrenzwert (AWG) is the German (IFA) equivalent of PEL. It replaces the older Maximale Arbeitsplatz Konzentration (MAK). International occupational exposure limits and many safety details can be retrieved from the large open-access database of the “Information system on hazardous substances of the German social accident insurance” GESTIS (Gefahrstoffinformationssystem) [453, 454]. As obvious from the tables associated with the norms, the values of the different limits are steadily decreasing. This fact reflects, on one hand, the increase in the known harmful consequences of diverse chemicals (thanks to progress in biology and medicine), and on the other hand a more restrictive definition of a healthy organism. Table 3 includes some example of concentration limits for the gases/vapor frequently addressed by researchers in the field of GGSs.

**Table 3:**
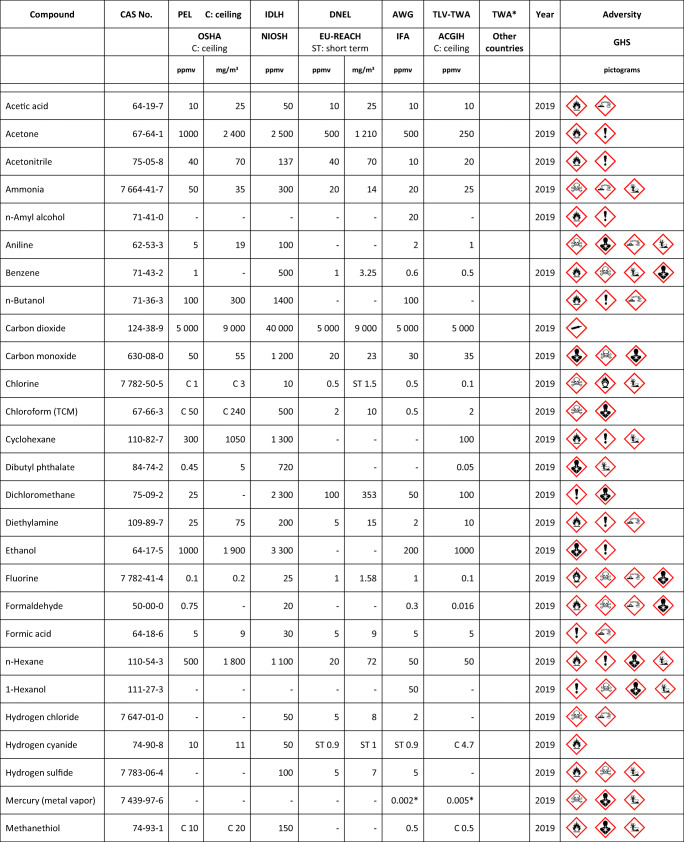

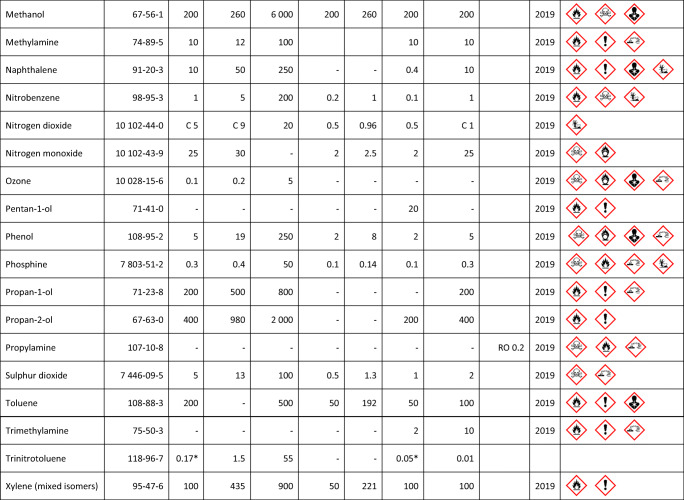
Some concentration limits for harmful gases. All data except IDLH values have been taken from the GESTIS databank (see the text). The IDLH values are reproduced from the online table of the NIOSH webpage. They are older than GESTIS data, that is, were published mainly before 1994 and only a few of them afterwards

*If no value is available for the selected norms (the previous columns), a known limit value from another country given for reference. The value is also taken from the GESTIS database and the country symbol is placed before

**Values converted from mg/m^3^ in ppmv by the review’s authors, assuming ppbv level of Hg vapor to ideal gas

Highly toxic substances, some possibly produced as chemical warfare agents (CWA) [455], are the subject of a different jurisdiction. The harmful effect of these substances is measured by the lethal dose/concentration (LD100/LC100) or median lethal dose/concentration (LD50/LC50), which gives the amount of the agent that would cause death with an indicated probability of 100% or 50% [456, 457]. The required toxicologic experiments are performed on test animals under legally/ethically acceptable conditions and extrapolated to humans. For safety reasons, the very toxic gases and vapors are replaced in gas-sensing tests with simulants, which are chemically very close to the substituted compounds but with no/low toxicity [456]. Lavoie et al. have given useful information on simulant types and their similarity to a large number of CWAs, suggesting chemoinformatic methods to detect them [458]. Additional information about toxic substances can be found on the NIOSH webpage or in EC No. 1272/2008 “Guidance on the Application of the CPL Criteria”. Table 4 includes a few CWAs and the simulants commonly investigated for gravimetric detection. As for flammable/explosive gases/vapors, there are no official standards concerning dangerous concentrations, because these concentrations express physicochemical properties of gaseous mixtures which cannot be influenced by norms. Legislation tries only to prevent and reduce the consequences of inadequate handling of related hazards. Several concentration limits are relevant for flammable mixtures. They emerge from the characteristics of combustion processes for different stoichiometric composition of the fuel (gas/vapor) and oxidant gas (oxygen), ambient pressure and presence of other gases (nitrogen in the case of air) [441, 459–462]. A too lean or too rich flammable mixture will not burn. The lowest concentration of fuel for which the fuel–oxidant mixture still burns is known as the lower flammability limit (LFL), and the highest as the upper flammability limit (UFL). Often addressed are the lower explosion limit (LEL) and upper explosion limit (UEL). These terms are not very appropriate. Indeed, according to the combustion speed, one has deflagration for subsonic processes (speed of combustion lower than the speed of sound in air) and detonation for supersonic processes. Both can produce an explosion, that is, blowing up the vessel in which the combustion takes place because of increased pressure. A flammability/explosion limit is always an IDLH level, since it is immediately dangerous to life and health. The LFL and UFL for some analytes are given in Table 5. More comprehensive tables can be found in the references above [441, 459–462]. A general perspective on hazardous chemicals is given in the “Hazardous Chemicals Handbook” of Carson and Mumford [463].

**Table 4:** Toxicity of selected CWAs and their simulants. Compiled from references [457] and [458]

CWA	CAS CWA	LD50	Simulant	Abbreviation	CAS Simulant	Similarity
		mg/min/m^3^				%
Sarin (GB)	107-44-8	35	Di-isopropyl fluorophosphate	DFP	55-91-4	87.5
			Di-isopropyl methyl phosphonate	DIMP	1 445-75-6	75.0
			Dimethyl methyl phosphonate	DMMP	756-79-6	66.7
Soman (GD)	96-64-0	35	Di-isopropyl fluorophosphate	DFP	55-91-4	84.0
			Di-isopropyl methyl phosphonate	DIMP	1 445-75-6	72.0
			Dimethyl methyl phosphonate	DMMP	756-79-6	64.0
Tabun (GA)	77-81-6	70	Diethyl ethyl phosphonate	DEEP	78_38_6	53.7
			Triethyl phosphate	TEP	38-40-0	52.5
			Diethyl 4-nitrophenyl phosphonate	Paraoxon	311-45-5	47.2
Distilled mustard (HD)	505-60-2	1000	2-Chloroethyl ethyl sulfide	CEES	693-07-2	64.7
			2-Chloroethyl methyl sulfide	CEMS	542-81-4	52.9
			Chloroethyl phenyl sulfide	CEPS	5 535-49-9	42.1

**Table 5:** Flammability limits of selected gaseous mixtures with air. Values taken from reference [461]

Compound	CAS	LFL	UFL	Remarks
		Volume %	Volume %	
Acetone	67-64-1	2.6	13.0	
Acetylene	74-86-2	2.5	100	
Ammonia	7 664-41-7	15.0	28.0	
Benzene	71-43-2	1.3	7.9	Both FL at 100°C
*n*-Butane	106-97-8	1.8	8.4	
Carbon monoxide	630-08-0	12.5	74.0	
Ethane	Z4-84-0	3.0	12.4	
Ethanol	64-17-5	3.3	19.0	UFL at 60°C
*n*-Hexane	110-54-3	1.2	7.4	
Hydrogen	1 333-74-0	4.0	75.0	
Methane	74-82-8	5.0	15.0	
Methanol	67-56-1	6.7	36.0	UFL at 60°C
*n*-Octane	111-65-9	0.95	–	
Propane	74-98-6	2.1	9.5	
Toluene	108-88-3	1.2	7.1	Both FL at 100°C

#### Overview of the target analytes detected by GGSs

In many cases, the specific detection of gases and vapor with GGSs is based on the weak interactions between the analyte and the receptor, as previously accounted for in the section “Receptors for MST” of the first (published [1]) part of this review and in the section “Increasing the specificity of the receptors” above. Combined with chemical and geometric complementarity, these interactions can result in molecular recognition and, consequently, in increased sensitivity and selectivity of the sensing process. In the context of the linear solvation energy relationship (LSER), numerous combinations of analyte–receptor material have been established, whose LSER parameters indicate the strength of different contributions to the recognition process [5, 464–466]. The chemical and physical properties of gases and vapors which might be relevant for gas sensing can be found in general chemistry textbooks [467–471] or technical literature [472–475]. The data from the technical literature will be used in the following for exemplifications without additional citation.

##### Volatile organic compounds

VOCs are the most commonly addressed target analytes for GGSs. In many cases, the sensitivity of one sensor for several VOCs is reported. This feature, frequently seen as a useful characteristic, indicates however limited sensor selectivity. Previous sections (“Increasing the specificity of the receptors” and “Evaluating the performance of the gas sensors and sensor arrays”) show how this drawback can be overcome by building up SAs and using chemometric methods.

**Fig. 22:**
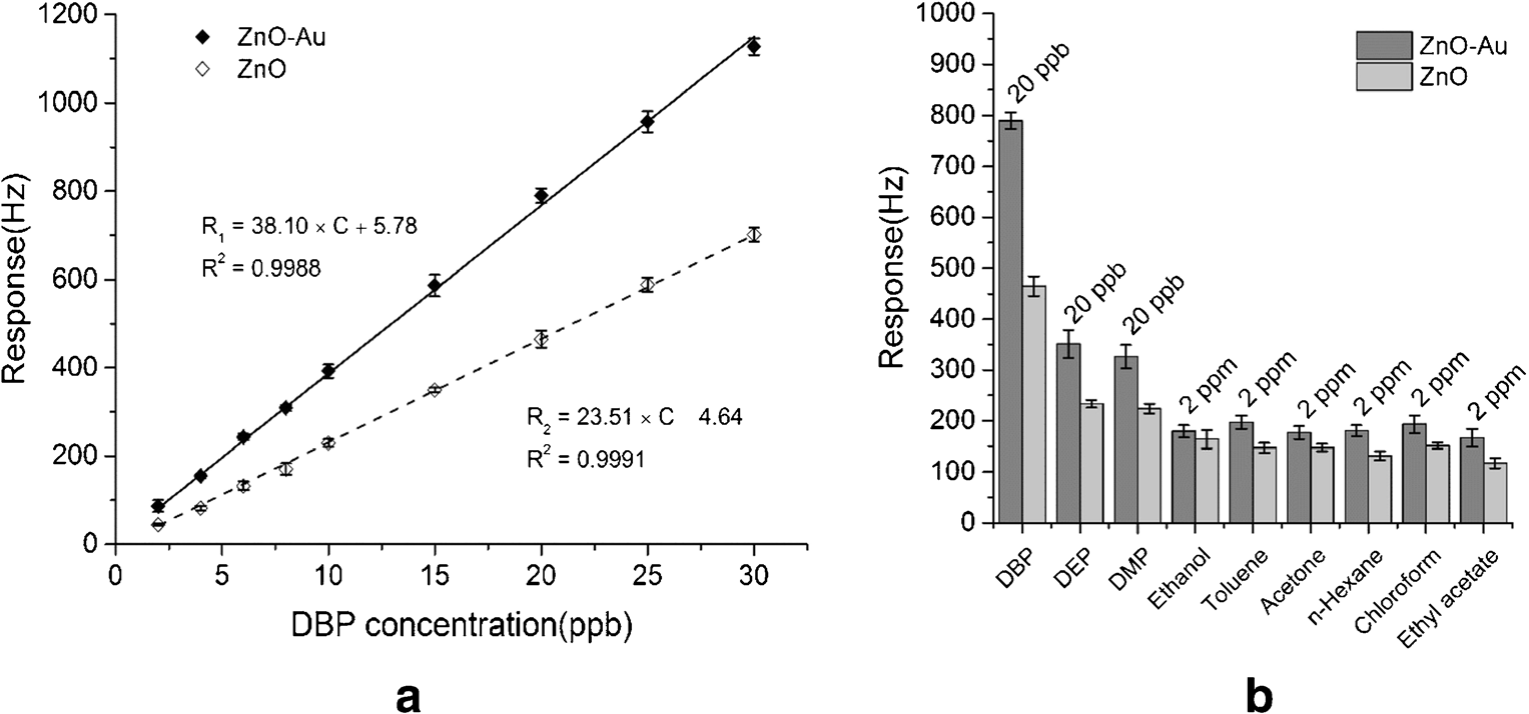
Dibutyl phthalate detection with Q-TSMRs coated with Au-decorated ZnO. (**a**) Calibration curve. (**b**) Cross-sensitivity to VOCs. DEP and DMP stand for diethyl and dimethyl phthalate, respectively. Reproduced with the kind permission of the authors and MDPI from reference [477]

**Fig. 23:**
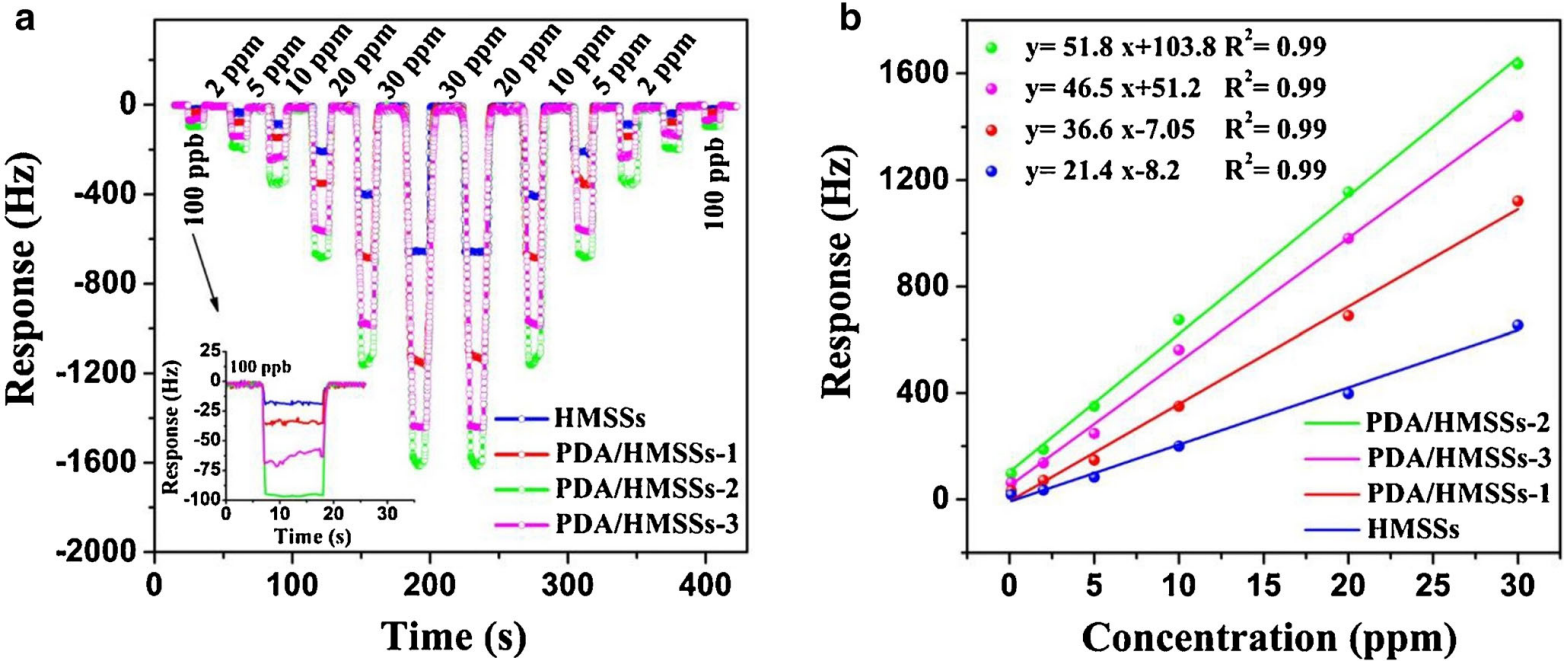
Formaldehyde detection with PDA-functionalized HMSS. (**a**) Dynamic response. (**b**) Calibration curves. Reproduced with the kind permission of Elsevier B.V. from reference [117]

- *Aromatic VOCs* are stable compounds, with low reactivity, mainly detected through hydrogen bonds with the receptor. Monocyclic arene vapors are mostly reported. They are rather volatile (12.7 kPa for benzene, 3.8 kPa for toluene and 0.79 kPa for xylene at 25 °C). Toluene is often reported, as the TLV-TWA is rather high (100 ppmv) while the vapor pressure (VP) is not too great. Poly(aniline) emeraldine salt thin films doped with different acids (hydrochloric, dodecylbenzene sulfonic, 1,5-naphthalene disulfonic) were deposited by dip coating on TSMRs as VOCs sensors [283]. Good sensitivity to aromatic VOCs in dry nitrogen was obtained, but it decreased in the presence of humidity. The influence of oxygen on layer stability, which might be an issue for the given material, was not discussed. Siloxanes including triMethylSilane (3MS), DiEthoxyMethylSilane (DEMS) and OctaMethylCycloTetraSiloxane (OMCTS), deposited on TSMRs through PECVD by Sabahy et al., were able to detect toluene at concentrations below TLV-TWA [312]. These siloxane layers were hydrophobic, with contact angles larger than 95°, and response achieved with only a few Hz at 35% RH variation. Even so, this reduced cross-sensitivity to humidity is still not enough for analyte concentrations below TLV, where the sensor responses to toluene are comparable to those of water. The same group of authors also used finite element theory to analyze the thermodynamics of the toluene sorption process in organic films [476]. Zhang et al. reported selective sensing of dibutyl phthalate (DBP) from 2ppbv to 30ppmv, with 0.66 ppbv LDL and 3810 Hz/ppmv sensitivity, performed with TSMRs covered with Au-decorated ZnO porous microspheres [477], as illustrated in Fig. 22. The cross-sensitivity to dimethyl and diethyl phthalates is significant, but the cross-sensitivity to other VOCs appears to be reduced in the plots, as they are given at levels much lower than the TLV-TWA of these compounds (see Fig. 22). The effect of humidity, which is not addressed, might also limit sensor performance. Öztürk et al. utilized Pd-doped ZnO nanorods electrochemically deposited on TSMRs to detect xylene and other VOCs [313]. The response to xylene was highest (3.3 Hz/ppmv, 5 ppmv LDL), but the sensitivity to other VOCs (mainly ethanol and propanol) was also good, leading to a critical selectivity issue. Very good sensitivity for toluene (LDL=1ppmv, that is 1% from TLV-TWA) was obtained by Yamagiwa et al. with both Q-TSMR and Si micro-cantilevers coated with self-assembled MOFs, but with reduced specificity, due to a strong response to ethanol [175]. Selectivity problems were also encountered by Kumar et al. in detecting toluene with phthalocyanine-covered Q-TSMRs, because the sensitivity to xylene was practically the same [115]. Bachar et al. compared chemoresistive and gravimetric (TSMR) SAs based on heavy polycyclic aromatic hydrocarbons [231]. The gravimetric SA exhibited different sensitivity for different compounds, while the chemoresitive SA did not. Both arrays were evaluated for alkanes, alcohols, mesitylene, ether and water with chemometric methods. They were able to separate the polar compounds (mainly alcohols) from the nonpolar ones (mainly alkanes), with the help of DFA, in humidity background from 5% to 80% RH.
- *Aliphatic VOCs.* Several classes of gases and vapors are included here. The aliphatic alcohols are polar and volatile (VP is 13 kPa for methanol and 0.65kPa for butanol), with different degrees of toxicity. The aldehydes are less polar. Formaldehyde is very toxic (TLV-TWA of 16 ppbv) and gaseous at room temperature, while acetaldehyde boils at just 20°C and is not as toxic as formaldehyde. Being slightly polar and gaseous or very volatile, the amines are toxic (TLV-TWA in 10 ppmv range). The alkanes are nonpolar and not very toxic but easily flammable. They are permanent gases for low molecular mass (until butane) at room temperature. Yang and He performed formaldehyde detection with graphene oxide coated on quartz TSMRs in the presence of humidity [478]. The specificity was good, except for ethanol, for which relevant cross-sensitivity was observed. For the same analyte, Wang et al. proposed a TSMR GGS having a novel copper (II) complex as sensing layer, which was very sensitive (LDL only 1.3% from PEL and 62% from TLV-TWA) and quite selective [120]. The cross-sensitivity to humidity was, however, significant for atmospheric values. Therefore, almost the same group of authors tested a hydrophilic poly(dopamine) passivated against humidity by a super-hydrophobic poly(*n*-octadecylsiloxane) film as sensing material for formaldehyde [187]. The influence of water was drastically reduced, but the detection limit decreased to 0.5 ppmv (value estimated by the authors of this review). Formaldehyde detection with hollow mesoporous silica spheres (HMSS) functionalized with poly(dopamine) (PDA) was reported by Zong et al. [117]. The authors employed Q-TSMRs MST operating at 10 MHz. The sensor performance is displayed in Fig. 23. A review of TSMR-based sensors for formaldehyde is provided by reference [479].

**Fig. 24:**
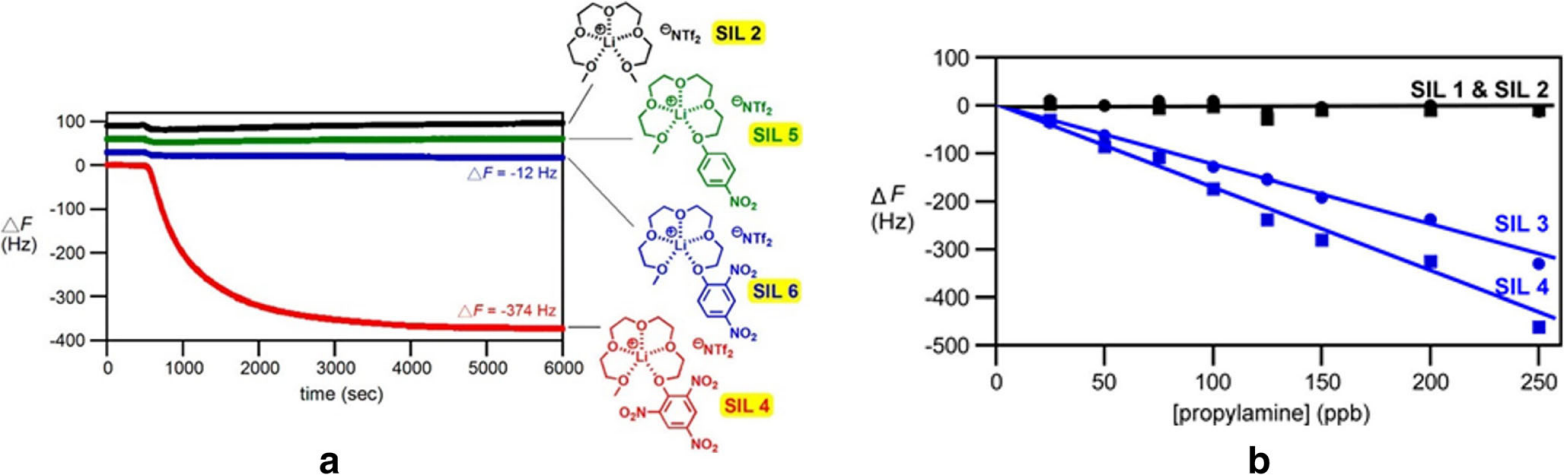
Propylamine detection with 9 MHz Q-TSMRs coated with ionic liquids. (**a**) Dynamic response. (**b**) Calibration curves. Reproduced with the kind permission of ACS Publications from reference [189]

Alcohols have been reported mainly in conjunction with practical applications such as VOC discrimination with LDA [240] and food quality evaluation [249, 480]. Amines have generated much interest. Das et al. demonstrate the use of polymerized castor oil for the detection of aliphatic amines (LDL of 10% from TLV-TWA) [281]. The sensitivity decreased slightly with amine molar mass, leading only to class recognition. Ammonia produces significant interference. Li and Chu obtained good sensitivity (see Fig. 24) to propylamine in measurements performed on Q-TSMRs coated with ionic liquids (5.4 ppbv LDL and 2000 Hz/ppmv sensitivity) [189]. The sensors were quite sluggish. Their response was not affected by traces of water, but a test of cross-sensitivity to humidity was not considered.

**Fig. 25:**
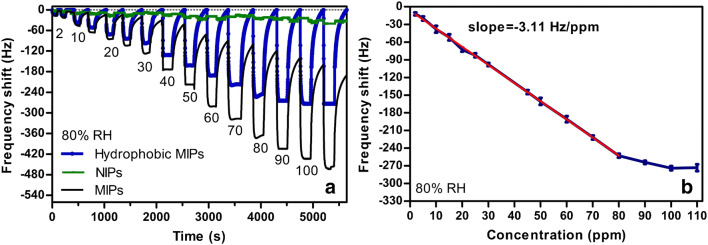
Hexanal detection with Q-TSMRs coated with hydrophobic MIP-SiO_2_ NP composite. (**a**) Dynamic response. NIP and MIP signals are provided by the non-imprinted and imprinted materials alone. (**b**) Calibration curve. Reproduced with the kind permission of Elsevier B.V. from reference [180]

Zhang et al. [182] and Chen et al. [481] reported on trimethylamine (TMA) detection with Q-TSMRs and GO-based layers (GO/chitosan and GO/Cu_2_O nanocomposite, respectively). Both receptor materials were sensitive (4.8 Hz/ppmv and 8.9 Hz/ppmv, respectively), but the GO/Cu_2_O nanocomposite had a much lower LDL (230ppbv) than that of GO/chitosan (1.3 ppmv). The cross-sensitivity to other amines was high, but the response to other VOCs was limited in the case of GO/chitosan films. The strong influence of humidity was a common issue for both sensors, acknowledged by the authors, who informed the reader that they would continue the investigation in this regard. To reduce cross-sensitivity to water, Chen et al. produced composite films of hexanal-imprinted MIPs and hydrophobic silica NPs [180]. The efficiency of the approach can be observed in Fig. 25. Methane detection with cryptophane-A/E films was reported by Wang et al. and Shen et al., respectively, using Q-SAW transducers at 300 MHz and 204 MHz [482, 483]. The sensitivity was sufficient to measure well below the LFL of 5%. However, in the case of cryptophane-A, 70% RH gave the same response as LFL methane concentration. For cryptophane-E, the cross-sensitivity to humidity was not reported. Molecularly imprinted polymers (poly(methyl methacrylate)) have shown good sensitivity for terpenes, but with significant cross-sensitivity to humidity [91].

**Fig. 26:**
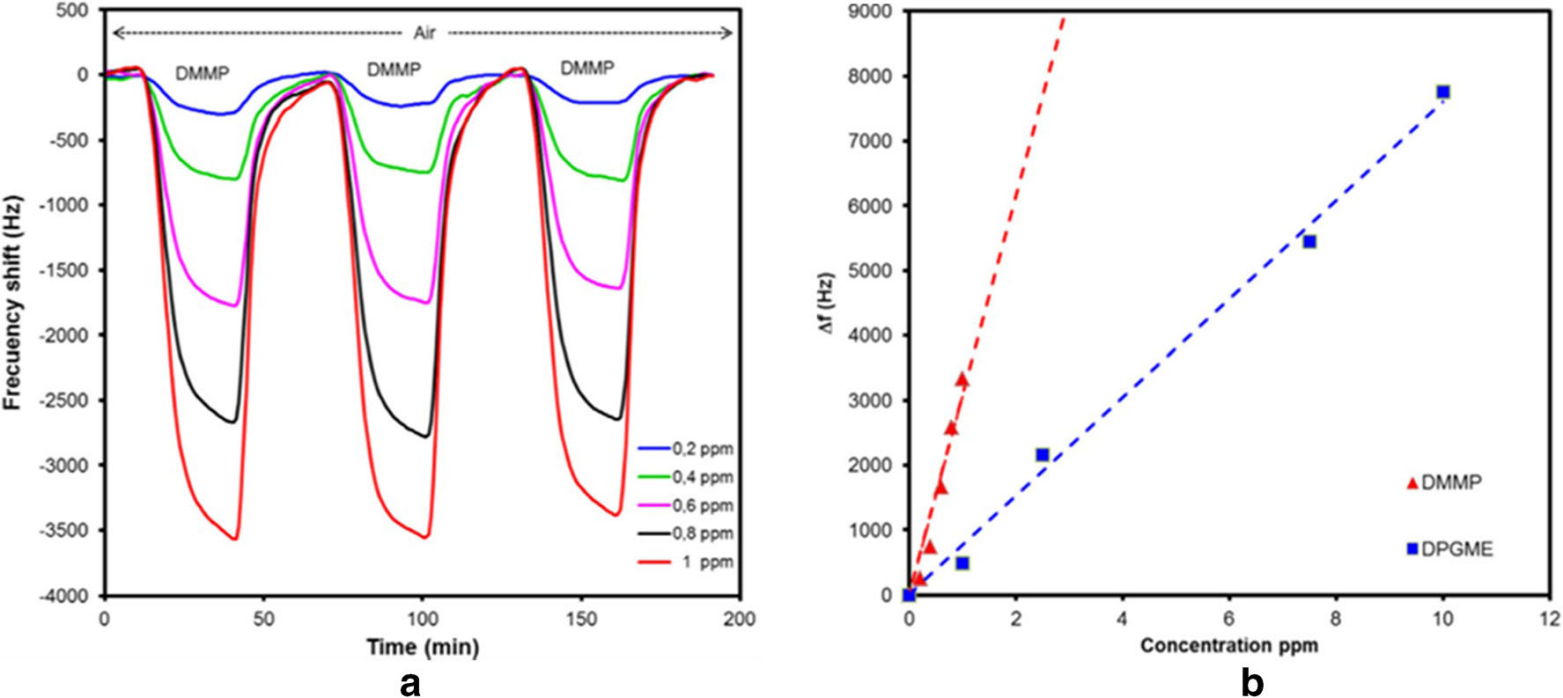
Dynamic response (**a**) and calibration curve (**b**) of a GO-coated Love-SAW sensor exposed to CWAs. Reproduced with the kind permission of Elsevier B.V. from reference [486]

- **Chemical warfare agents and other special analytes.** As specified above, the detection of CWAs has been investigated on simulants, most of them included in Table 4. TSMR with mesoporous TiO_2_ -SiO_2_ functionalized with p-hexa-fluoroisopropanol aniline (HFIP) showed acceptable sensitivity and specificity towards the nerve agent simulant DMMP [118]. The influence of humidity was not discussed. Sulfur mustard and DMMP sensors based on composite materials were reported by Lal and Ziwari [179] (see the subsection 'Composite, polymorph and unusual receptor materials' above) and Yang et al. [484]. Di Pietrantonio et al. used laser-induced forward transfer (LIFT) to deposit thin polymer layers on Q-SAW, which were very sensitive to the simulant DMMP [485]. The experiments were repeated on real CWA (sarin in this case) with even better performance (649 Hz/ppmv sensitivity and 150 ppbv LDL). Despite certain selectivity displayed by different polymers, a dedicated cross-sensitivity test was not performed, but was planned. Employing a Love-SAW array coated with six dissimilar polymers, Matatagui et al. also achieved very good performance for CWA detection (40,200 Hz/ppmv sensitivity and 40 ppbv LDL) [229]. Moreover, they were able to discriminate the tested agent simulants, with scores in the first two PCs nicely clustered for the whole concentration range. In a later investigation by almost the same authors, a GO sensing layer was chosen [486]. The good sensitivity of the device is demonstrated by the plots in Fig. 26. Chen et al. achieved successful detection of DMMP with a wireless Q-TSMR having both resonance frequency and dissipation output [487]. The MST was covered by hollow ball-like indium oxide and enabled 2.1 Hz/ppmv sensitivity and an LDL below 5 ppmv. The vapors of explosive nitroaromatic compounds 2,4-dinitrotoluene (2,4-DNT) and trinitrotoluene (TNT) were specifically recognized by TSMRs coated with methylated mesoporous silica [437]. This approach was already addressed in “Unconventional theoretical and numerical approaches to sensor data analysis” above. The same types of compounds were detected by Eslami and Alizadeh with poly(pyrrole) (PPy)–bromophenol blue (BPB) layers on Q-TSMRs [488]. Comparative responses for several analytes including TNT, [3-nitrooxy-2,2-bis (nitrooxymethyl)propyl] nitrate (PETN), 1,3,5-trinitroperhydro-

**Fig. 27:**
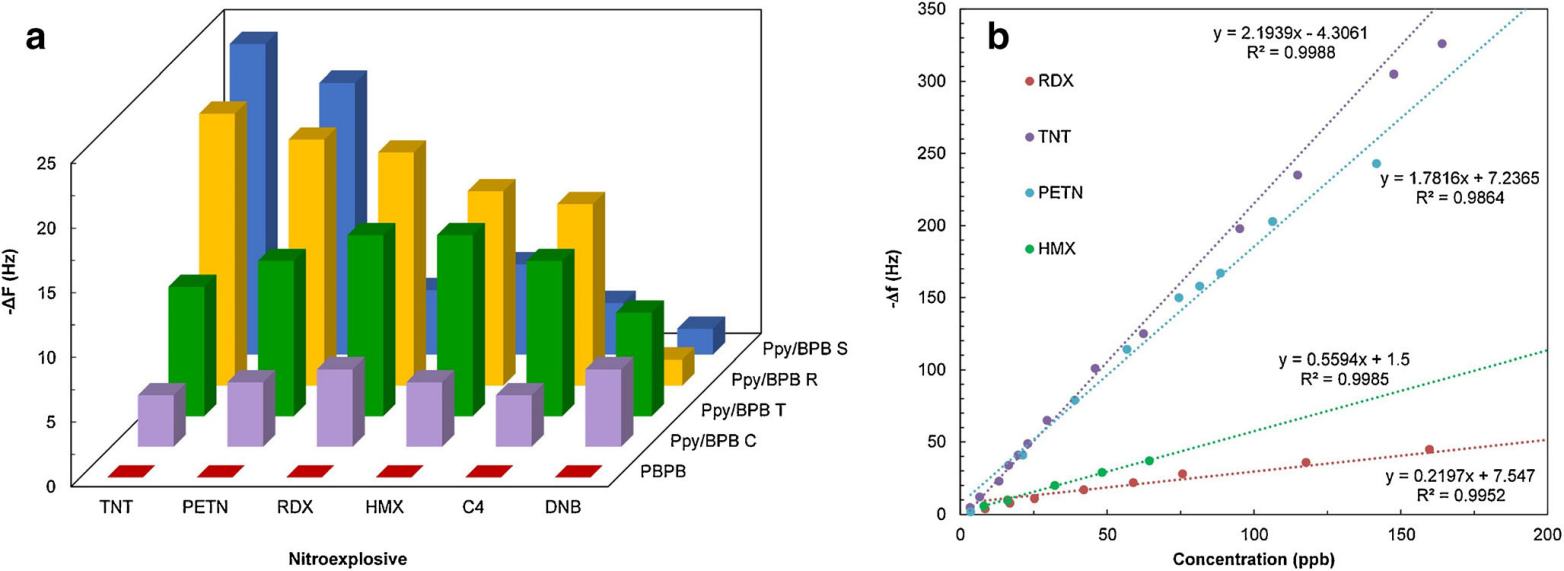
(**a**) “The response of differently prepared sensors including poly BPB (PBPB), PPy-BPB co-polymer (PPy/BPB C), PPy/BPB thin film (PPy/BPB T), PPy/BPB nanospheres (PPy/BPB S) and PPy/BPB nanorods (PPy/BPB R) toward nitroexplosives” (original caption). (**b**) The calibration curves of the PPy-BPB/QCM sensor for TNT, PETN, RDX and HMX with low LDLs (500 ppt for TNT, 800 ppt for PETN, 1 ppbv for RDX and 2 ppbv for HMX). Reproduced with the kind permission of Elsevier B.V. from reference [488]

1,3,5-triazine (RDX) and octahydro-1,3,5,7-tetranitro-1,3,5,7-tetrazocine (HMX) given by the selected sensing materials are included in Fig. 27. The detection of toxic hydrogen cyanide with CuO nanoparticles appears promising from the contribution of Yang et al. [489]. The material seems to have certain selectivity towards HCN due to the unexpected positive gravimetric response of the coated TSMRs, opposite to the “normal” response to the other tested analytes, which was negative. The authors attributed the sensor behavior to mass loss during recognition redox reaction. However, the response in atmosphere containing both analyte and interfering gas was not evaluated, despite possible compensation of the effects. Also, the humidity was kept constant, at a low level of 10%, hindering a more realistic characterization. In a follow-up paper, the authors reconsidered the investigation and obtained high cross-sensitivity to humidity [490]. Precursors of illicit compounds have been recognized and detected with high-frequency Q-TSMRs (195 MHz) coated with five microporous triptycene-based affinity materials [491]. Prantl et al. did not report metric and cross-sensitivity data, but the approach seems interesting.

**Fig. 28:**
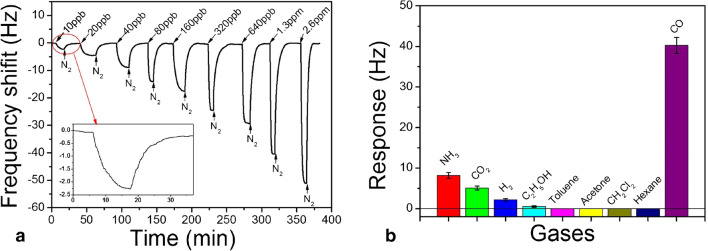
Dynamic response to carbon monoxide (**a**) and cross-sensitivity (**b**) of a micro-cantilever coated with Ni-MOF-74. Reproduced with the kind permission of Elsevier B.V. from reference [495]

**Fig. 29:**
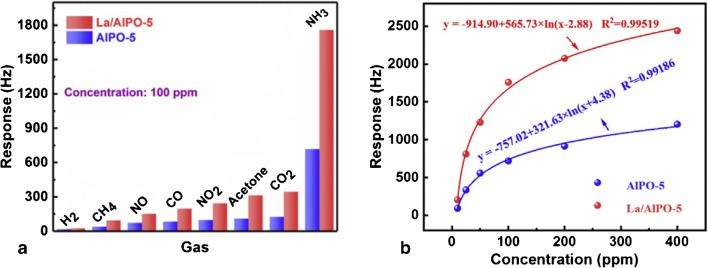
The responses to different analytes (**a**) and the calibration curves for ammonia (**b**) of Q-TSMRs coated with AlPO-5 and La-doped AlPO-5. Reproduced with the kind permission of Elsevier B.V. from reference [158]

**Fig. 30:**
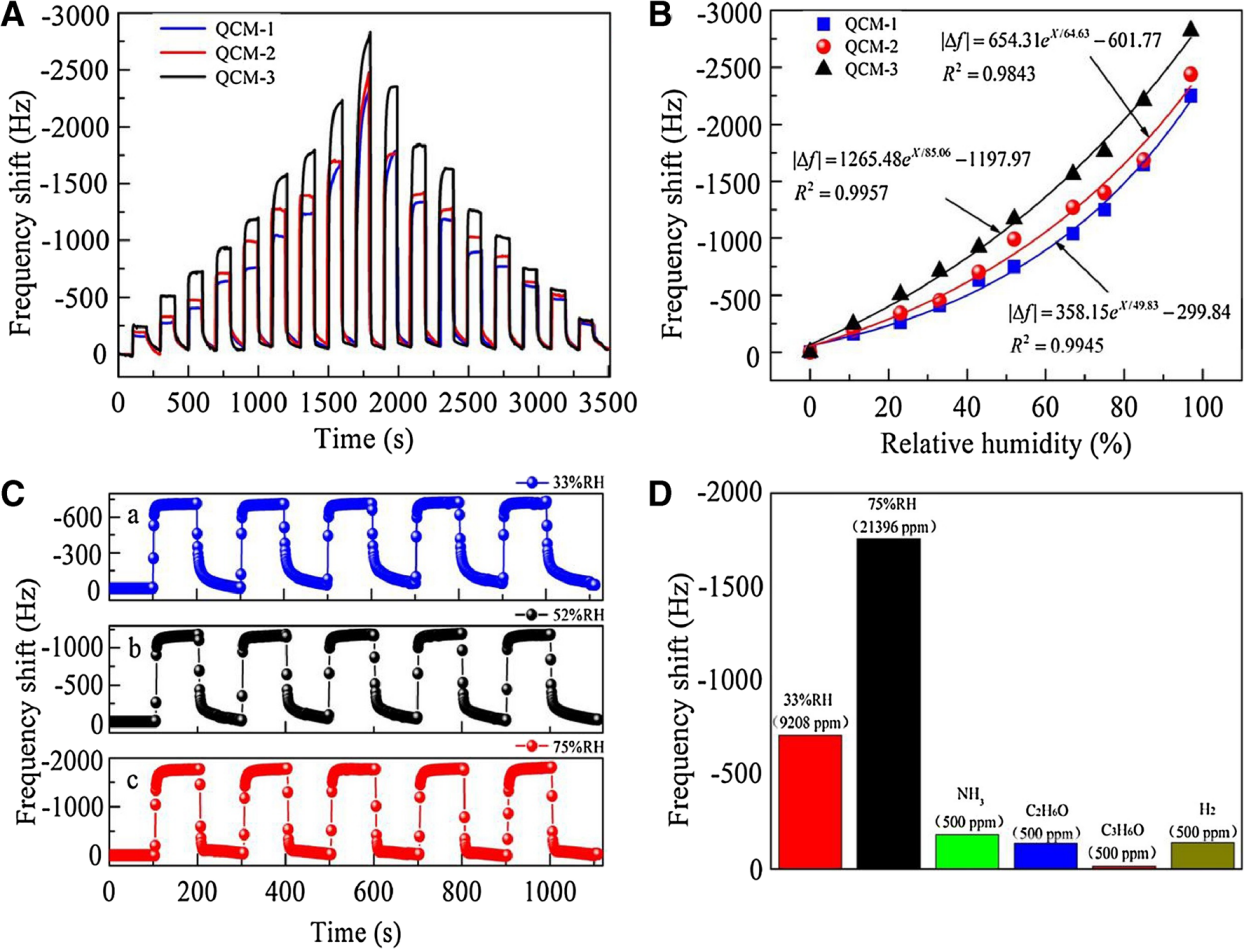
(**a**) “Dynamic frequency shift of the three QCM sensors switching under various RH levels.” (**b**) “Frequency shift of the three QCM sensors as a function of RH.” (**c**) “Repeatability of the QCM-3 sensor switched from 0%RH to 33%RH, 52%RH and 75%RH.” (**d**) “Selectivity of the QCM-3 sensors towards RH and various gas species." (Original caption; here RH stands for relative humidity). Reproduced with the kind permission of Elsevier B.V. from reference [287]

- **Inorganic gases.** From the class of inorganic gases, those most investigated are the hazardous gases (CO, CO_2_, NO_2_, SO_2_, HN_3_, H_2_S) whose presence in the atmosphere is of major concern. CO detection has been addressed several times by researchers in the field. Ippolito et al. employed for this purpose a heated SAW coated with In_2_O_3_ [304]. Its operating principle was already addressed in the paragraph “SAW transducers” of the first review part [1]. H_2_, NO_2_ and CO were detected at room temperature when the same type of SAW was coated with a poly(aniline)/In_2_O_3_ nanofiber composite [492]. Bayram et al. detected CO with ferrocene-branched chitosan derivatives on quartz TSMR, but with rather low sensitivity [493]. Tian et al. obtained acceptable SO_2_ and NO_2_ sensitivity and selectivity with electropolymerized ring-substituted (2-methyl, 2-metoxy) poly(aniline)s on TSMRs [494]. However, it was not possible to evaluate concentrations separately if both analytes were present in the sample. Using micro-cantilevers coated with metal organic framework crystals of Ni-MOF-74 (also Fe, Mn and Mg tested with less success), Lv et al. were able measure CO concentrations below 10ppbv due to the large BET surface area of the receptor material (1150m^2^/g) [495]. The signals were strong, but the response/recovery times were also long, due to rather high adsorption enthalpy of ~53kJ/mol (see Fig. 28). Good repeatability, selectivity, long-term stability (sensitivity degradation of 1% in 6 months) and manufacture reproducibility (12% spread over 4 samples) indicated a good sensor performance. The calibration curves were strongly nonlinear, favoring the low concentration levels. The influence of humidity, however, was not addressed. Wang et al. detected (see Fig. 29) low ammonia levels (60 ppbv LDL) with high sensitivity (100Hz/ppmv) with 10 MHz Q-TSMRs covered by La-doped framework AlPO-5 [158]. Cross-sensitivity to humidity, carbon dioxide, acetone and nitrogen dioxide were discussed. Hydrogen sulfide detection has been addressed by two authors. Asad et al. evaluated H_2_S concentrations between 5 and 200 ppmv using a LiNbO_3_ SAW operated at 104 MHz and room temperature [136]. The sensing material, Cu-NP-SWCNT, was drop-cast on the MST surface, and conferred high sensitivity (3750 Hz/ppmv) and reasonable selectivity. Responses ~10 times lower than those for hydrogen sulfide were obtained for ethanol, hydrogen and acetone. At operating temperatures below 100 °C, the sensor response was influenced by humidity, but thanks to the high-temperature piezoelectric material of the transducer, operation at over 100°C could be considered and the water vapor removed from the sensor surface. The H_2_S sensor prepared by Li et al. is based on a Q-SAW-MST, spin-coated with CuO prepared by a sol–gel route [496]. It has 4kHz/ppmv sensitivity and 0.5 ppmv LDL in a humid environment. The authors tried to explained this behavior through the shift in the equilibrium of the reaction *H*_2_*S* + *CuO* ⇌ *CuS* + *H*_2_*O* due to increased water concentration, but they produced no evidence in this respect. The cross-sensitivity to VOCs and inorganic gases was low. Hydrogen is not toxic, but the evaluation of its concentration in the range of LFL (4%) has fire safety relevance. Viespe and Miu reported good hydrogen detection (59 ppmv LDL and 0.51 Hz/ppmv sensitivity) with Pd/Zn bilayers produced by pulsed laser deposition (PLD) on 70MHz Q-SAWs [497]. Using only Pd films on silicon micro-cantilevers and static bending operating mode, McKeown et al. detected 250 ppmv of hydrogen in air [498].
- **Humidity.** Humidity is the third/fourth component of atmospheric air (around 1% volume for 50% RH at room temperature, like argon). It is not toxic, but water vapor concentrations that are too low or too high cause discomfort. Additionally, it is the main interferent in the case of environmental gas sensors. This issue, as already addressed at the beginning of the first review part [1], emerges from naturally large values of absolute humidity in the atmosphere, much larger than the detected levels of almost any other analyte. Traditionally, one evaluates the relative humidity, that is, the ratio of the water partial and saturation vapor pressure at the measurement temperature. This brings additional inconvenience, because the absolute water concentration for a given relative concentration increases with temperature following Antoine’s law (as does the saturation vapor pressure) [469]. Although polymer capacitive sensors are the state of the art in humidity sensing, several studies still investigate sensors based on MSTs [499]. This interest is probably because most of the plain sensing materials have large dispersion interaction, which favors the detection of the analyte with highest concentration, usually humidity. Yuan et al. used spray coating to deposit thin nanocomposite layers of poly(ethylenimine)-graphene oxide on quartz TSMRs which were sensitive to humidity [186]. The resulting sensors exhibited low cross-sensitivity to formaldehyde, carbon dioxide, ammonia and sulfur dioxide, reduced response and recovery times, and good long-term stability. TSMRs based on a graphene oxide/tin dioxide/poly(aniline) (GO/SnO2/PANI) composite fabricated via in situ oxidative polymerization by Zhang et al. showed wide pore size distribution and large surface area but low contact angle, being well suited for humidity detection [287] (see Fig. 30). Xuan et al. achieved good humidity estimation with GO deposited by spin coating or drop casting on 140 MHz and 225 MHz ZnO-SAWs manufactured on glass substrates [500]. The sensors had exponential calibration curves, which could be linearized for low humidity (7 Hz/ppmv=1.448 kHz/%RH in this range). Many authors have reported on humidity sensors based on different materials such as mesoporous SnO_2_–SiO_2_ [501] or porous poly(methyl methacrylate) [502]. The measurement of humidity at atmospheric concentrations seems to be an easy task, even in the presence of other gases, because of the large absolute values of water concentration (see above and the paragraph “The performance of GGSs” of the first review part), which leads to an increase of the relative response to humidity with respect to almost any other compound at the TLV-TWA level. However, condensation, possibly connected to the presence of interfering gases, can result in significant inaccuracy [503]. In order to facilitate sensor calibration at low humidity, Tsukahara et al. developed an original method to produce water vapor at the ppmv level [504], which used the controlled retention of water on the inner surface of a pipeline.

### Targeted applications

The reports on GGSs seldom specify only a targeted application for the sensors studied. As shown in the previous paragraph, most of authors indicate the main analyte and sensor performance, leaving open the choice for its future use. There are, however, several exceptions. Among these, food quality control, medical instrumentation, detection of very dangerous industrial emissions and ambient air quality are the most relevant.

#### Freshness, quality and flavor of food

**Fig. 31:**
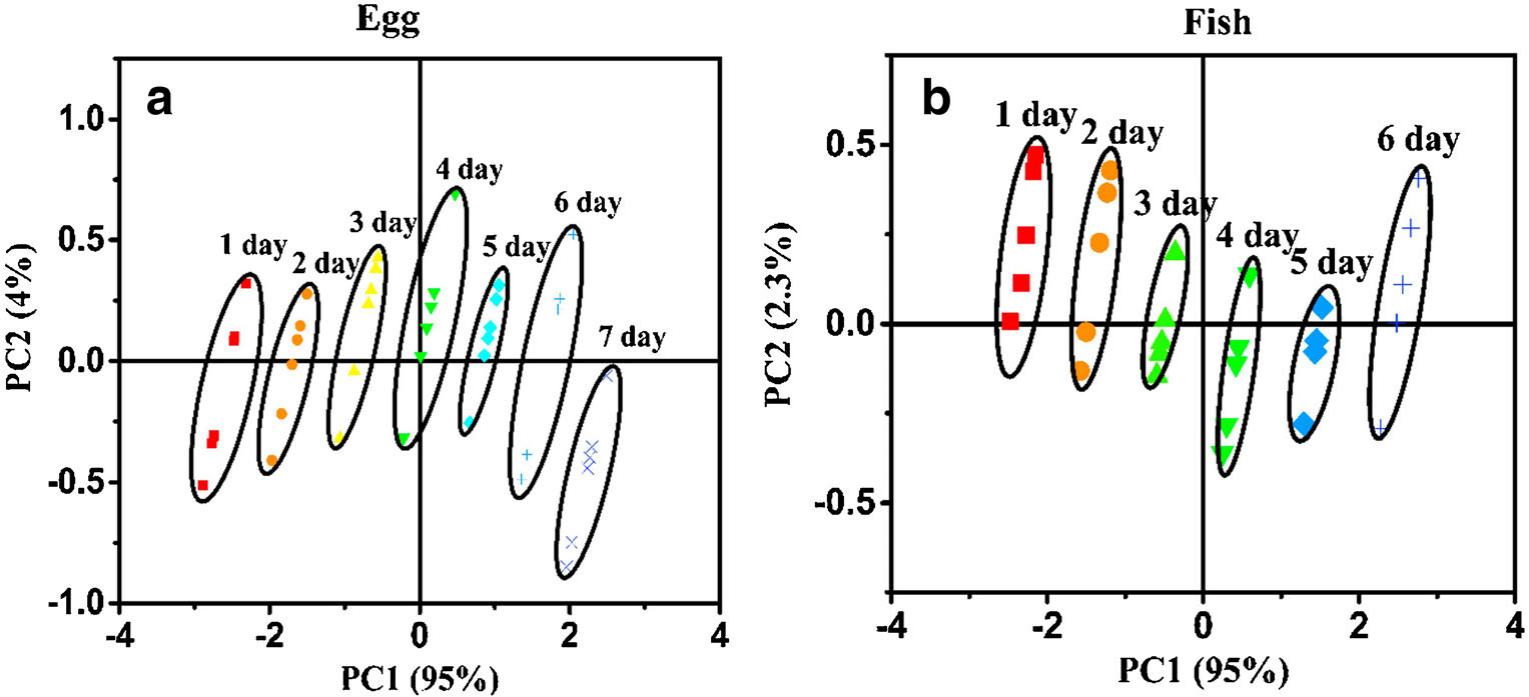
PCA score clustering for stored fruits according to storage time. Evaluation made with GGS array based on poly(pyrrole) and TiO_2_ composite. Reproduced with the kind permission of Elsevier B.V. from references [480]

**Fig. 32:**
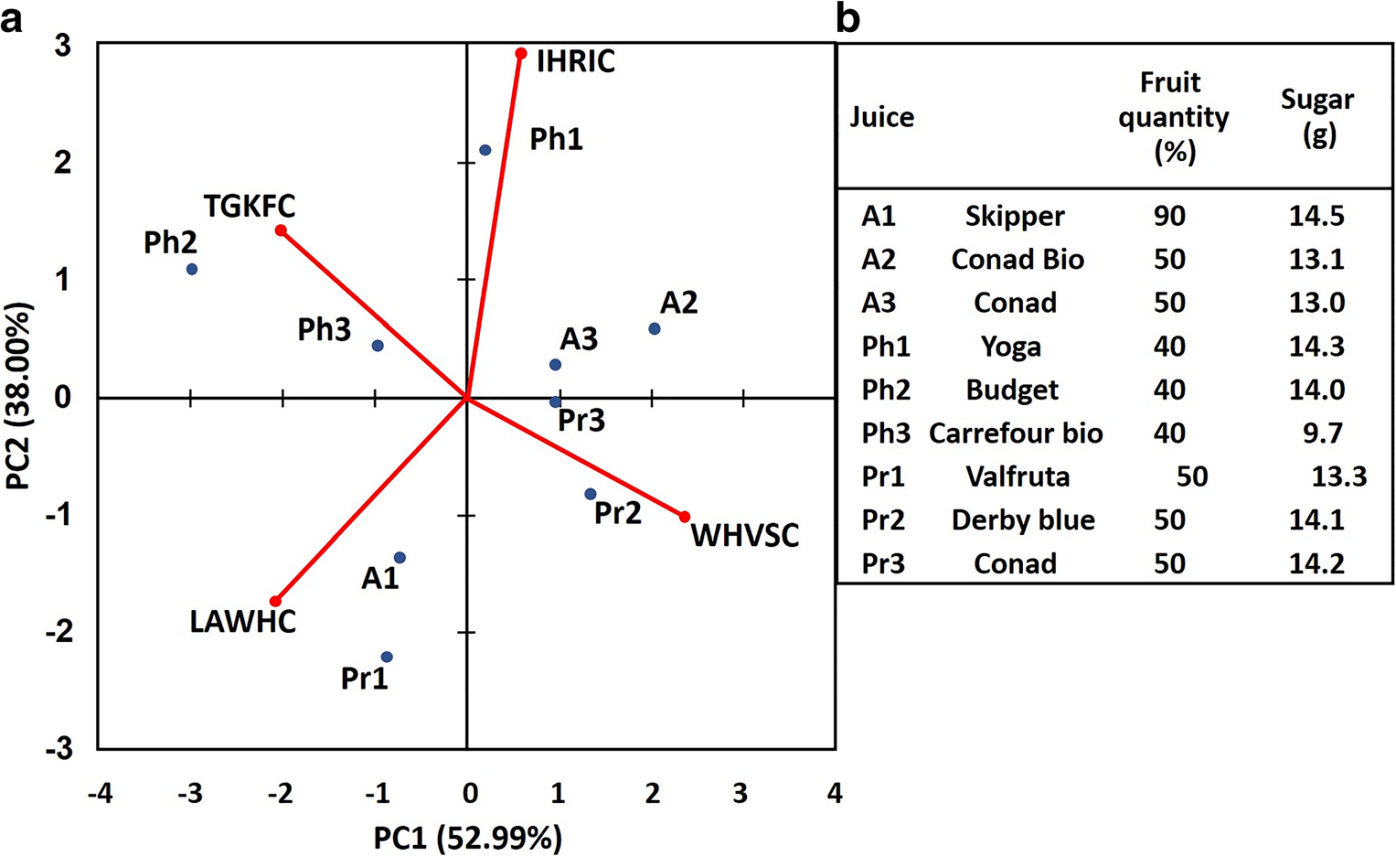
The biplot of the scores and loadings in the first two PCs resulting from the responses of a four-sensor SA when exposed to headspace samples of fruit juices. The table in the right panel indicates the average composition of these commercial beverages. Reproduced with the kind permission of Frontiers Media SA from reference [249]

Traditionally, quality assessment in the food industry has been performed by panels of human experts. Researchers in the field of gas sensing have tried to automate and simplify this process by building dedicated SAs and evaluating their data with chemometric methods. Some approaches are based on GGSs only. Sharma et al. analyzed the headspace vapor containing linalool to discern the flavor of black tea [436]. The pseudo-chemometric procedure was already explained in the section “Evaluating the performance of the gas sensors and sensor arrays”, last paragraph (“Unconventional theoretical and numerical approaches to sensor data analysis”). The fermentation of black tea has been monitored through a similar approach [505]. TSMRs coated with poly(dimethyl siloxane) were employed to evaluate the ripening degree of mango fruits after the released amount of 3-carene [506]. The assessment of virgin oil quality through gravimetric arrays was reported by Escuderos et al. [338]. A percentage of 91.7% correct classification was obtained in the first two PCA components. Compagnone et al. used two types of TSMR-based GGSs for quality control of chocolate [103]. Both the porphyrin and the gold-nanoparticle-peptide GGS were able to separate the batches with standard flavor from the rest in the frame of PLS-DA. With peptide/gold nanoparticle Q-TSMR arrays, it was possible to detect/discriminate food aromas [51]. Toniolo et al. estimated food quality by means of a Q-TSMR array coated with ionic liquids [342], while Cui et al. assessed the degree of freshness/storage time of fish, eggs and mangos [480] (see Fig. 31). The TSMRs were covered with poly(pyrrole)/TiO_2_ nanocomposite assembled through layer-by-layer self-assembly. Using ZnO NPs modified with four different molecularly modeled peptides, Mascini et al. produced a Q-TSMR array with low sensitivity to humidity which could separate VOCs relevant for fruit juice identification [249]. The PCA biplot in Fig. 32 demonstrates the right choice of the sensing materials (according to the loadings of the four sensors indexed as WHVSC, LAWHC, IHRIC and TGKFC) and the good separation of the juice scores in the first two PC planes. The freshness of eggs was checked by Deng et al. using a Q-TSMR SA dip-coated with different receptor materials (multi-walled carbon nanotubes, graphene, copper oxide and poly(aniline)) [343]. The PLSR and PLSR/KPCA plots of the predicted versus actual storage times of eggs were already given as an example in Fig. 18, and confirm the success of the attempt. The potential for a polymer-functionalized SAW SA to recognize coffee flavor through the scores in the first two PCs was analyzed by Barié et al. [507].

#### Medical and biological applications

**Fig. 33:**
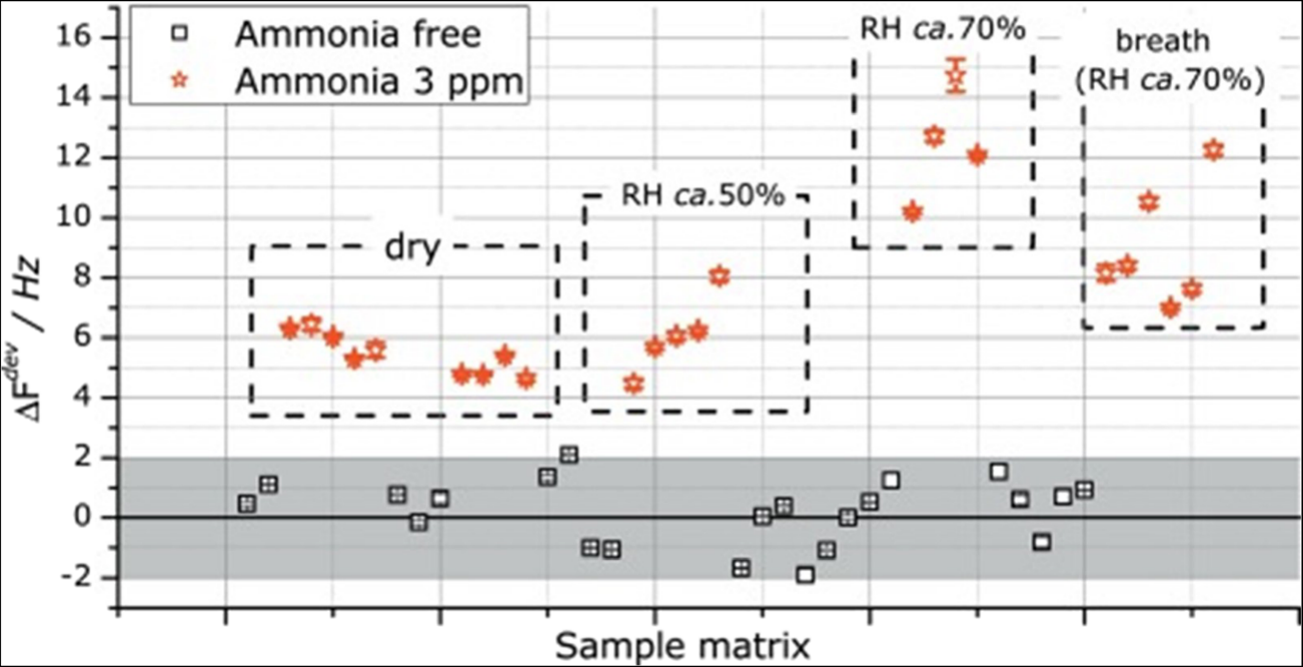
The response of a differential Q-TSMR sensor system for ammonia detection in large humidity background (refer to the text). The comparison with the exhaled ammonia in human breath demonstrates the potential of the approach. Reproduced with the kind permission of Elsevier B.V. from reference [119]

**Fig. 34:**
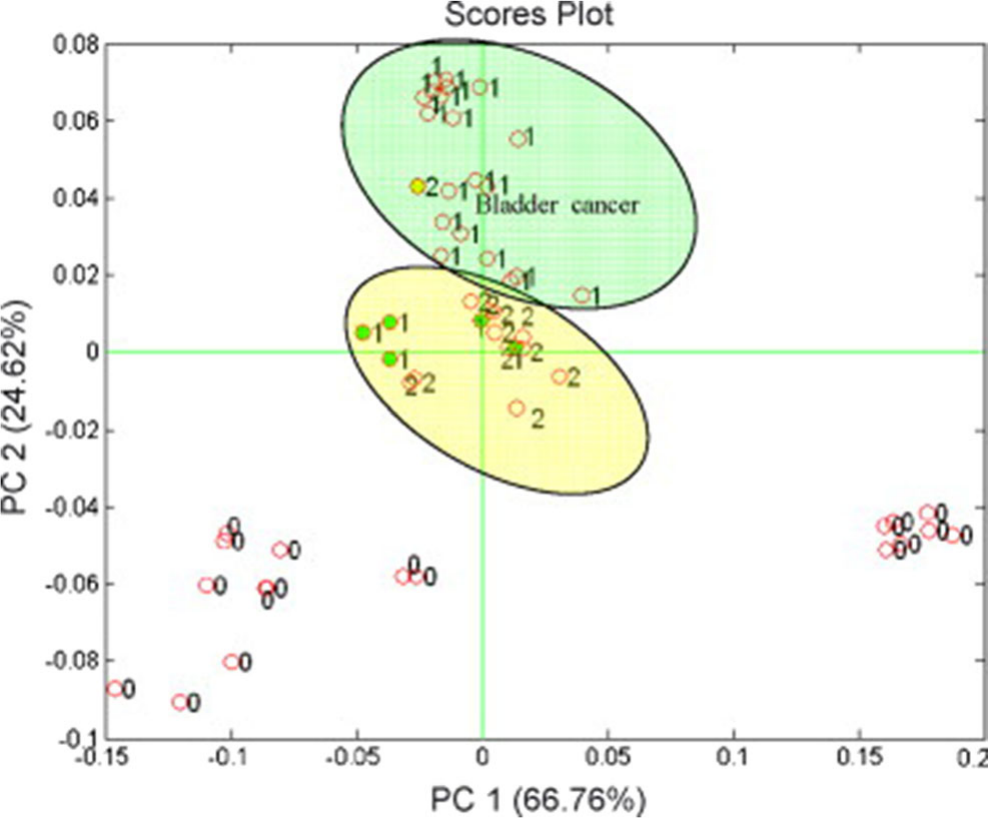
The PCA score plots with spontaneous clustering of urine analysis results. The event scores are displayed in the first two PCs as red open circles. Events numbered “0” and without cluster structure belong to healthy persons. The events numbered “1” and “2” indicate patients having bladder (green cluster) and prostate (yellow cluster) cancer, respectively. Some bladder cancer events are spread among the prostate ones. They are displayed as green filled circles with red contour. Reproduced with the kind permission of Elsevier B.V. from reference [344]

The increasing need for noninvasive and cheap investigative tools for medical use has led to growing interest in gas sensor research in this field. Because the gaseous markers of diseases are released in very small amounts from clinical samples, sensors with very low LDLs and high sensitivity are suitable. In the case of breath analysis, the sample is taken from the patient exhalation and contains water vapor almost at saturation (66,000 ppmv at human body temperature). These special operating conditions can seldom be fulfilled by individual sensors, and SAs and chemometric methods are typically employed to gather the medical information. Ogimoto et al. prepared porous films comprising silica nanoparticles/poly(allylamine hydrochloride) (SiNPs/PAH) infused with poly(acrylic acid) (PAA) on Q-TSMRs to detect ammonia in breath analysis as a biomarker of renal insufficiency or hepatic dysfunction [119]. Using two transducers, one coated with SiNPs/PAH and the other with SiNPs/PAH/PAA, the authors built a differential sensor system able to subtract the influence of breath humidity (see Fig. 33) and to measure the ammonia concentration of the physiological samples. A breath analysis array of six Q-TSMRs coated with organic and composite nanometric films was devised and tested by Selyanchyn et al. [246]. Because the sensor system considers the influence of the temperature and exhalation flow, extended health control can be performed. Jha and Hayashi succeeded in detecting organic acids or aldehyde in body odor with a Q-TSMR array covered with molecularly imprinted polymers [247, 248]. The experimental data resulted in good clustering in the first two PCA components. Also, diseases which are not “visible” in exhalation can be detected with GGSs, like urinary tract cancer. Bernabey et al. employed an EN based on eight Q-TSMR sensors with dissimilar porphyrin receptors to discriminate scents among three groups of individuals: healthy persons and bladder and prostate cancer patients [344]. The evaluation of the experimental data with PCA showed reasonable separation trends. However, a few patients with bladder cancer were included among those with prostate cancer. No healthy person was identified as ill (see Fig. 34).

De Cesare et al. employed a gravimetric EN to detect the “olfactory fingerprints” of microorganisms in soil, with potential relevance for agriculture [340]. The eight Q-TSMRs of the nose were coated with organic polymers and exposed to the headspace atmosphere generated by the investigated samples. Discrimination of PCA scores between non-inoculated and bacterially inoculated soils was possible. Using PLSR, the authors could predict, with reasonable accuracy, the number of inoculated microorganisms.

#### Metallic vapor

Industrial emissions can release metallic pollutants into the environment, often directly into the atmosphere, as metallic vapor. For example, Kabir et al. measured the concentration of metallic quicksilver vapor at the ppbv level (1.4 ppbv LDL) with a SAW transducer covered with a Au sensing layer and having Ni electrodes (to avoid amalgam formation) [508]. Instead of gold as sensing material, silver has also been used [509]. Sabri et al. created Au nanoparticles on the electrodes of a Q-TSMR to detect Hg vapor with 2.4 ppbv LDL [510].

When considering the achievements addressed in this section reflecting the results reported by several authors, one has to keep in mind that these successes have been obtained in investigations carried out under very controlled conditions, in a friendly laboratory environment, and many more real-world studies will be required before the applications can be widely used.

## Achievements and perspectives on gravimetric gas sensors

The first conclusive accounts below refer to topics contained in this part of the review and are followed by statements emerging from the whole review. They attempt to establish the degree to which the expectations in the field of GGSs have been met and to understand why the advances did not mirror the prospects.

### Efforts to improve gravimetric gas sensor performance and their practical implementation

Because of their simple transduction, appropriate for all recognition processes resulting in receptor mass change, GGSs have been and remain a very attractive choice for applications. As the whole section “Increasing the specificity of the receptors” shows, many attempts have been made to increase the strength and specificity of the analyte–receptor interaction through several types of approaches:

- increasing the complementarity of the receptor towards a given analyte by molecular tailoring, molecular imprinting, use of molecular symmetry (the chiral one, for example), use of cavitands and/or functionalized compounds, use of materials with an enhanced degree of thermodynamic non-ideality (composite, polymorphs, ionic fluids)
- by means of GGS arrays or systems with improved selectivity
- employing adequate sensor data processing at the hardware (analogic) level, or through online/offline dedicated software, based on chemometric methods (PCA, CA, DA, PLS-DA, PLSR) or artificial intelligence
- developing evaluation approaches for specific devices/applications.

The methods used to coat the MSTs with the sensing layers, chosen to be compatible with the required structure/morphology of the material, are very diverse, ranging from simple (like drop casting, spray coating) to complicated (like atomic layer deposition, biosynthetic methods), sometimes including compound preparation stages (for instance, electro-polymerization). The devised devices have been utilized for the detection of different classes of gases/vapor (flammable/toxic hydrocarbons, inorganic gases, CWAs, humidity, metal vapor), in some cases in the context of well-defined applications: freshness and quality of food, identification of human diseases and personal safety in industrial activity. However, the characterization of the reported devices or applications is not exhaustive or adequately relevant. Several researchers do not thoroughly use their experimental data to infer the sensor parameters (this can be seen in Annex, which encompasses many parameters evaluated by the authors of the present review from data already existing in the published reports). Moreover, the chemometric methods are employed to a limited extent, even for sensor arrays, where they would contribute to an increase in the sensor system specificity and accuracy.

### The actual development stage of gravimetric gas sensors: expectations, achievements and disappointments

Despite good scientific understanding and technological progress, the expected breakthrough of GGSs has occurred to a very limited extent in the application field and is almost invisible on the market. Even after increasing the effort spent for devising adsorption-based devices, the market situation did not improve. Some possible reasons are:

- the mismatch between the operational parameters required by the industrial/customer applications and the “offer” coming from the prototype GGSs.
- the large market request for the detection of certain gases (toxic and flammable/explosive ones) for which the sensing capability and mainly the specificity of GGSs is limited. The optimistic picture of GGSs selectivity in the literature is often misleading, because the cross-sensitivity tests, though performed at relatively high concentrations with respect to that of the target analyte, are not as large as TLV-TWA levels which might actually be encountered in the atmosphere. The most critical is the case of humidity, where the usual environmental level is about 10,000 ppmv.
- the questionable reliability, mainly due to the soft organic material employed for the receptor manufacture, but also to the mechanical component of the transducer operation (resonant ultra-acoustic vibration). The changes in the sensing layer stiffness, occurring together with changes in the mass, might also alter accurate responses.
- the sensor price, largely decided by the price of MTS (often using single crystals) is usually higher than the one of some competing categories of devices such as SMOX chemoresitive or polymer capacitive gas sensors.
- the accuracy of the GGSs is below the one offered by the electrochemical or optical/spectral sensors, which are superior even if more expensive and bulkier.
- the readout is still more complicated than in the case of the other sensors.

## Final remarks

The investigation on gas sensing devices, particularly gravimetric gas sensors, is continuously growing in importance, volume and geographic extent. The considerable interest in this scientific and applicative topic, witnessed by the increasing number of published reports, publications and patents, reflects the increasing market needs but also the limited success of the previous research approaches in the field. Here, one has to acknowledge the still unripe stage, at least from a market point of view, of gravimetric gas sensors, even after decades of investigations leading to pertinent scientific knowledge. This uncommon situation has, nevertheless, a positive side. It shows that many opportunities are still open, and innovative solutions for gravimetric gas sensors are needed and anticipated.

## Annex

**Table 6:** Overview of the characteristics, performance and applications of the gravimetric sensors reported in literature. The values included in the table are the best reported by the authors. The capital letter “**E**” precedes the parameters which are not given in the reference but could be evaluated by the review’s authors from the information available in that reference. The missing information, not possible to be recovered by the authors of the review, is indicated by the character “**-**“. Sometimes, when two types of gas sensing devices are reported, or to extend the space in the cells, two rows have been used

Analyte Best preform	Concentration	Sensing material	Deposition method	Structure / morphology	MST	Freq.	Single / SA	Sens.	Detect. limit	Resp / Rec times	Reported interferents	Chemometric methods	Applications	Remarks	Reference	
	ppmv					MHz		Hz/ppmv		s						
VOVs	5 – 700	Polymers	Spray coating	Thin films 0.5 – 12 μm	Si μ-Clv integrate	350	SA 4	0.1	1	120/-	Temperature	PLSR	VOCs detection	LDL=1% TLV toluene	2002	[216]
VOCs Toluene		MIPS poly(acrylic), poly(methacrylic)	Drop coating, spreading	Thin films Nano sphere	Q-TSMR	10	6	E 0.27	E 20	E 30/300	-	UC (linear)	VOCs detection	LDL=20% TLV Partition ratios	2003	[89]
VOCs toluene	> 17 000 5 – 140	SW-CNTs & Cd arachidate	LB	nano- composite	SAW, Q-TSMR	433 10	6 6	43.7 0.01	E ~5	60/- - /-	-	UC (linear)	VOCs detection	LDL=5% TLV	2005	[260]
VOCs 1-Butanol	400 - 1400	Polymers	μ-Drop coating	Tin film	Si μ-Clv	0.02 - 0.15	SA 6	0.16	-	-	-	UC (linear) PCA, PLSR	VOCs detection MC	Mixture calibration	2006	[253]
VOCs	-	Polymers	Ink jet spotting	Thin films	Si μ-Clv	0.05	SA 8	-	-	-	-	PCA sores	VOCs clustering Breath analysis	Virtual array 40 Sens. Electronic nose	2007	[263]
DMMP	0.2 - 20	MIP poly(o-phenylenediamine)	Electrochemical	Thin film 10 nm	Q-SAW	300	1	400	0.1	E 500/800	-	UC (linear)	DMMP detection	No TLV available	2007	[255]
CO, NO	25 - 75	Co porphyrin	Immersion in solution	Thin film	Q-TSMR	20	1	1.5	E <5	E 20/50	Humidity, VOCs	UC (linear)	CO, NO detection	LDL=20% TLV	2009	[511]
VOCs	> 50 000	IL Conducting polymer	Casting from solution	Thin film	Q-TSMR	10	S 4 integrated	100μ	-	-	Humidity	UC (linear)	VOCs detection	To low sensitivity	2009	[217]
VOCs Butanol	500 - 2300	monolayers on Au NP	Two phases reduction	NP	Q-TSMR	10	4	E ~35	-	-	-	UC (linear)	LSER parameters	-	2009	[512]
Nitroaromatic compound	0.01 – 0.24	Metalloporphyrin on diamond NPs	Layer by layer	Thin films 40 – 60 nm	Q-SAW	433	4	120 000	0.001	250/1600	Humidity, ethanol	UC (linear)	DNT, TNT detection	LDL=10% TLV	2010	[513]
VOCs	40 -10 000	Phthalocyanines substituted	Spray coating	Thin film 400nm	Q-TSMR	10	SA 14	E 0.08	10 - 100	-	Humidity	UC (linear) PCA biplots, CA	Partition ratios	Fundamental research focused	2010	[107]
VOCs 1-Hexanol	25 - 500	Poly(3-hexyl thiophene)	Drop coating	Thin film Regio regular	Q-TSMR	10	4	E ~0.14	1.5	180/120	-	UC (not linear) PCA scores	VOCs detection	LDL=3% AWG	2011	[341]
VOCs n-Butanol	1 - 400	TiO_2_	Sol - gel	Thin film	Q-TSMR	10	3	E ~0.7	E ~3	E 60/120	-	-	Correlation Wiener index	LDL=3% PEL	2011	[514]
Formaldehyde	0.05 - 100	Polyamide composite	Electrospun	Thin film Nanofibers	Q-TSMR	5	3	3	0.05	-	Humidity	UC (linear) lo level only	Formaldehyde detection	LDL=7% PEL LDL=300% TLV	2011	[515]
Hg	0.12 – 1.27	Au	Electro-deposition	Nanoprism 100 – 200 nm	Q-TSMR	-	1	0.0024	E 1 270	E 2000	Humidity, ammonia	UC (linear)	Hg detection	LDL=50% TLV	2011	[510]
TNT TMA, NH_3_	0.000 045 - 1	Functionalized silica SBA-15	Drop coating	Thin film Mesoporous	Si μClv	0.101	2	E 20 000 E 140	E 0.00005 E 0.05	60/120 300/-	Ethanol, Benzene	UC (linear)	Target analyte detection	Very sensitive LDL=0.5% TLV (TMA)	2011	[516]
Hydrogen	5 000 – 40 000	Pd/CuPc	PVD	Thin bilayer 18/80 - 200 nm	LiNbO_3_-SAW	0.125	1	E 0.01	E 2 000	4/-	CO, SO_2_	UC (linear and not linear)	Hydrogen detection	LDL=5% LFL	2012	[517]
Formic acid	0.5 - 10	Poly(aniline)	Electro-chemical	Thin film	Q-TSMR	1	1	1.7	E 0.7	E 2000/-	-	UC (linear)	Formic acid detection	LDL=14% TLV	2012	[518]
CWA DMMP	0.04 – 200	Polymers	Spray coating	Thin films 100 nm	Love - SAW	163	6	40 200	0.04	E 600/600	-	UC (linear)	CWAs detection	LDL=2% LD50/RT Sensitive sensor	2012	[229]
Acetic acid	10 - 100	Synthetic peptides	SAM	Roughness 2-4 nm	Q-TSMR	10	1	2.2	2.0	E 300/400	-	UC (linear)	FOOFD quality	LDL=200% TLV	2012	[59]
CWA DMMP	2 - 35	Polymers	LIFT	Thin films 4 – 10 nm	Q-SAW	392	3	649	0.015	E 60/60	Ethanol, DCM	UC (linear) PCA	CWA detection PCA clustering	No TLV data available	2012	[485]
DMMP HCN	4 - 30	Metal oxide (WO_3_) nanostructures	-	Nanostructures	Q-TSMR	-	9	70 E 3	E 0.1 E 2	30/73	-	UC (linear)	Analyte detection	No TLV data available LDL=20% TLV	2012	[484]
Hydrogen sulfide	2 – 20	SnO2/CuO composite film	RF magnetron sputtering	Composite film 40 nm	LiTaO_3_-SAW	147	1	16 900	0.53	60/45	Hydrogen, ethanol	UC (linear)	Hydrogen sulfide detection	LDL=11% AWG	2012	[519]
VOCs mesitylene	100 - 400	Poly aromatic hydrocarbons	Drop coating	-	Q-TSMR	-	SA 6	-	-	-	Humidity	UC (linear) PCA, DFA	VOCs detection discrimination	3 PCs scores 3 CVs	2013	[231]
VOCs α-Terpineol		Room temperature ionic liquids	Drop coating	Thin films 300-500 nm	Q-TSMR	8	SA 7	1130	0.002	-	-	UC (linear) PCA scores	Food quality, discrimination	Very sensitive	2013	[342]
FOOD aromas	Head space	Au-NP - peptides	Self-assembling Drop casting	Thin films 30-50 kHz shift	Q-TSMR	20	SA 7	-	-	-	-	PCA scores, loadings	VOCs, Olive oil clustering	-	2013	[51]
VOCs (polar)	130 – 13 000	Vic-dioximes & their metal complexes	Spray coating	Thin films 22 kHz shift	Q-TSMR	10	SA 8	0.1	-	E 100/100	Humidity	UC (linear) PCA biplots	VOCs detection	Partition ratios	2013	[243]
Ammonia	50 – 800	Vertically orientated ZnO nanorods	Wet chemical route	Nanorod length 1.2 – 3 μm	Q-TSMR	5.48	4	0.23	E 5	E 800/800	-	UC (not linear)	Ammonia detection	LDL=20% TLV	2013	[520]
Odors Octenol	10 – 90	Odorant binding proteins	Drop coating	Thin films 25kNz shift	Q-SAW	392	3	25.9	0.39	E 30/30	-	UC (linear)	Odor detection	No TLV available	2013	[521]
VOCs, CO, CO_2_, NO, methane	5 – 200 000	Corrole	Spray coating	Thin films 12kHz shift	Q-TSMR	10	8	E 4	5	300/-	-	UC (linear & not linear)	VOCs detection, gas detection	Enough sensitivity	2013	[101]
Dibutyl phthalate	0.02 – 1.2	Poly(aniline)	Drop coating	Nanofiber Film 3-25 μm	Q-TSMR	6	E 2	E 300	0.02	30/-	Acetone, ethanol	UC (linear)	Dibutyl phthalate detection	LDL=4% TLV	2013	[522]
TMA	5 – 1 000	AuNP–RGO	Drop coating	Thin layer porous	Si μ-Clv	0.1	3	E 0.75	E 5	30/30	HCHO, acetone, humidity	UC (linear)	TMA detection	LDL=50% AWG	2013	[138]
Hydrogen sulfide	5 - 200	SW-CNT decorated with Cu NP	Drop coating	Thin films	LiNbO_3_-SAW	104	1	E 3750	E 2	35/50	Humidity, Ethanol, H_2_	Dynamic response	Hydrogen sulfide detection	LDL=40% AWG	2014	[136]
VOCs	30 -30 000	Cyclic Tetrapyrroles	Dip coating	Thin film	Q-TSMR	5	2	0.53	0.8	35/55	-	UC (mostly linear)	VOCs detection	LDL=0.8% TLV	2014	[105]
VOCs Ethanol	40 - 200	Hexamethyl-disiloxane	PE - CVD	Thin film 320 nm	Q-TSMR	5	2	0.25	12	E 100/30	Humidity	UC (linear)	VOCs detection	LDL=1.2% TLV	2014	[523]
VOCs Xylene		Poly(aniline) (PANI) emeraldine salt	Dip coating	Thin film 100-300 nm	Q-TSMR	10	3	7.0	E ~3	180/60	Humidity	UC (linear)	VOCs detection	LDL=3% TLV	2014	[283]
VOCs Toluene	40 - 1000	MOFs	SAM	500 nm	Q-TSMR Si μ-Clv	9 0.9	2 SA 8	30 4	1 -	E 60/300	-	UC (linear)	VOCs detection	LDL=1% TLV	2014	[175]
Aliphatic amines	5 - 250	Castor oil polymerized	Dipping	Tin film	Q-TSMR	10	6	0.9	E <1	E 120/120	Ammonia	UC (linear)	Aliphatic amine detection	LDL=10% TLV	2014	[281]
Linalol	10 - 900	Poly(ethylene glycol)	Electrostatic spray	Thin films	Q-TSMR	10	1	E	0.3	E 30/-	Linalool oxide	UC (linear)	Linalool detection	No TLV data	2014	[436]
VOCs Methylamine	> 1 000	Chitosan	Drop coating	Film 2680 nm	Q-TSMR	5	2	0.5	E ~80	E >300 -	Temperature	UC (linear)	VOCs detection	LDL=800% TLV !?	2014	[524]
Organic acids in body odor	-	Molecularly imprinted poly(acrylic acid)	Spin coating Drop coating	Thin film >100 nm	Q-TSMR	9	SA 3	-	-	5/14	-	PCA, SVM	PCA scores cluster SVM discrimen.	Classification of mixtures	2014	[248]
Toluene	35 - 500	Tetra-tert-butyl copper phthalocyanine	Thermal evap. Drop casting	Thin films 400 nm	Q-TSMR Q-TSMR	5 10	3 1	0.1 -	30	180/180	NO_2_ O_3_, Xylene	UC (linear)	Toluene detection	LDL=33% TLV	2015	[115]
VOCs Octenol		Odorant-binding protein	MAPLE	Tin films 200 - 500 nm	Q-SAW	392	2	56	0.18	E 100/-	-	UC (linear)	VOCs detection	No TLV available	2014	[52]
VOCs		Peptides	Drop casting	Thin film 18-22 kHz shift	Q-TSMR	20	6	-	-	-	-	PCA Scores, biplots	Fundamental investigation	Virtual evaluation of affinity	2014	[56]
TNT	0.13	MBS, MNA, AET	Dip coating	Thin film	Si μ-Clv	static	3	-	-	-	-	-	Comparative investigation	-	2014	[525]
Ammonia	5 – 120	ZnO/SiO_2_	Spin coating	Bilayer 130 nm	Q-SAW	200	5	100	E 3	30/20	-	UC (not linear)	Ammonia detection	LDL=12% TLV	2014	[526]
Humidity	5 000 – 10 000	GO	Spin coating Drop casting	Thin films 70 -300 nm	ZnO-SAW	140 225	12	7	5 000	1/19	-	UC (exponential)	Humidity estimation	-	2014	[500]
Hg	0.02 - 0.37	Au	Evaporation	50 nm film	Q-SAW	131	1	12 k	0.0007	E > 2000	Humidity, VOCs, NH_3_	UC (not linear)	Hg detection	LDL=14% TLV	2015	[527]
VOCs Chloroform	> 10 000	Supramolecular monolayers	?	?	FBAR AlN / Si	4 400	SA 4	20	-	E 30/30	-	UC (linear)	VOCs detection	Ultrahigh frequency	2015	[232]
Ammonia	1 -40	Carboxyl Graphene poly(styrene)	Electro-spinning	Thin films composite	Q-TSMR	5	1	E 0.08	E 5	E 70	-	-	Ammonia detection	LDL=20% TLV	2015	[181]
Ammonia	0.3 - 100	Porous SiO_2_ & PAH poly(acrylic acid)	Electrostatic self-assembly	Composite Films	Q-TSMR	9	SA 2	E 18	0.5	E 30/100	Humidity	UC (linear)	NH_3_ detection Breath analysis	LDL=4% TLV	2015	[119]
HCl	100	poly(N-isopropylacrylamide)	Grafting on Au electrode	Thin film Brushes	Q-TSMR	9	many	-	-	120/900	-	-	HCl detection	No calibration provided	2015	[528]
VOCs Butanol		SW-CNT Modified surface	Spray coating	33 000 nm	Q-TSMR	10	1	E 0.07	-	E 10/20	-	UC (linear)	VOCs detection	To low sensitivity	2015	[259]
VOCs Acetic acid	-	metal porphyrins Au NP peptides	-	Films	Q-TSMR	-	SA 8	-	-	-	-	PLS-DA	Food quality, chocolate	Classification 3 LV Scores	2015	[103]
Aldehydes Hexanal		MIP Poly(acrylic acid)	Spin coating	Tin films Hundreds nm	Q-TSMR	9	SA 4	-	-	5/12	-	PCA, SVM	Body odor	Classification	2015	[247]
Illegal substances	-	PDMS & SW/MW CNT	Spin coating	Bilayers 36 – 395 nm	Q-TSMR	6	17	-	-	-	-	Chromatograph column	Chromatograph Column sensor	Can detect drugs	2015	[529]
DMMP	0.3 – 1.5	hyper-branched polymer	Self-assembling	Thin layers 110 – 200 nm	Si μ-Clv	0.025	5	E 2.2	E 0.3	E 90/30	Ethanol, NH_3_, toluene, H_2_O	UC (linear)	DMMP detection	LDL=2.5% LD50/RT Sensitive sensor	2015	[303]
Hydrogen cyanide	3 - 30	Ni(OH)_2_	Drop coating	Nano flowers	Q-TSMR	9	1	E 3.3	E 1	E 200/150	Humidity	UC (linear)	Hydrogen cyanide detection	LDL=10% PEL	2015	[530]
Hydrogen sulfide	5 – 40	SnO_2_	Sol-gel	Thin layer	Q-SAW	-	1	E 400	15	-	CO, CH_4_	UC (linear)	Hydrogen sulfide detection	LDL=30% AWG	2015	[531]
Hg	0.026 – 0.41	Ag, Au on poly(styrene)	Electron beam evaporation	Monolayer Nanospheres	Q-TSMR	10	4	E 480	0.0002	2 700/3 700	Humidity, VOCs	UC (not linear)	Mercury vapor detection	LDL=4% TLV	2015	[315]
Breath	-	Polymer and SiO_2_ NPs	Layer by layer deposition	Porous film 20 – 500 nm	Q-TSMR	9	6	-	-	-	Temperature	-	Breath detection	-	2015	[246]
VOCs	10 – 900	Glucose derivative	Electrostatic spray coating	Tin layers	Q-TSMR	10	SA 8	0.4	E <10	-	-	PCA	Food quality	-	2015	[505]
Nitrogen monoxide	0.001 – 200	Cu2+/PANI/WO3	Spin coating	300 nm	Q-SAW	98.47	1	E 22 000	E 0.0005	97/37	Humidity	UC (logarithmic)	NO detection	LDL=0.002% TLV	2015	[532]
Carbon dioxide		Graphene-nickel- L-alanine	Electrochemical	Multilayer film	Q-SAW	434	4	2.07	200	E 60/60	Ethanol	UC (linear)	CO_2_ detection Air quality	LDL=4% TLV	2015	[533]
Hydrogen cyanide	5 - 50	CuO	Drop coating	NPs	Q-TSMR	9	1	0.82	E 1	E 120/ 300	Humidity	UC (linear)	HCN detection	LDL=10% PEL	2015	[490]
Dibutyl phthalate	0.002 – 0.07	Au decorated ZnO	Drop coating	Thin layers	Q-TSMR	6	2	3 810	0.00066	50/50	VOCs	UC (linear)	Dibutyl phthal. detection	LDL=0.15% TLV	2015	[477]
VOCs Xylene	3 - 800	Pd doped ZnO	Electro-chemical	Nano rods 100–200 nm	Q-TSMR	10	5	3.3	E ~5	E 200/500	-	UC (linear)	VOCs detection	LDL=5% TLV	2016	[313]
VOCs Methanol	-	Polymers	Spin, Spray, Dip coating	-	Paper Clv	Static bend	SA 8	-	-	-	-	LDA	VOCs discrimination	-	2016	[240]
CO	5 - 90	PEDOT:PSS/PVP	Electrospun	Composite Nanofibers	Q-TSMR	8	1	E 0.8	E ~5	E 30/150	-	UC (linear)	CO detection	LDL=20% TLV	2016	[534]
Biomarker Acetone		TiO_2_ & MW-CNT	Spin coating	Thin film Composite	Q-TSMR	10	SA 3 integrated	4.6	4.5	-	-	UC (linear)	Acetone detection	LDL=2% TLV	2016	[218]
3-Carene	10 – 1 000	Poly(dimethyl-siloxane)	Electro Spray deposition	Thin film	Q-TSMR	10	5	0.06	E ~10	E 5/-	Beta-CF Furaneol	UC (linear)	3-Carene Mango ripening	Enough sensitivity	2016	[506]
Humidity	0 - 25 000	Poly(ethylenimine) GO	Dip coating	Film 3 kHz initial shift	Q-TSMR	10	3	-	-	3/5	NH_3_, NO_2_, CO_2_, H_2_S	UC (exponent.)	Humidity evaluation	Hot tested below 30% RH	2016	[183]
DMMP	0.2 - 10	Acetylcholinesterase RGO	Casting spreading	Thin film Bimorph	Q-TSMR	10	1	8	E 1	E 300/300	-	UC (linear)	DMMP detection	Enough sensitivity No TLV data	2016	[184]
Aromatic hydrocarbons	500	Phthalocyanines	Thermal evaporation	Thin film 200 – 400 nm	Q-TSMR	5	5	-	-	-	-	-	Toluene detection	One concentration only	2016	[535]
NO_2_, CO, toluene	0.05 – 4 000	MW-CNTs	Spray coating	Thin film	PZT μ-Clv	0.07	1	E 14	0.05	-	-	UC (logarithmic)	Analytes detection	LDL=10% AWG NO_2_	2016	[536]
NH_3_, TMA, H_2_S, Ethanol	10 – 2 000	Poly(pyrrole) - TiO_2_	Self-assembling Layer by layer	Polymorph composite	Q-TSMR	8	1	E 1.4	E <10	E 100/100	-	UC (linear), PCA clustering	Food quality	LDL<40% TLV Dissipation measured	2016	[480]
Ammonia	1 - 80	Cellulose acetate GO poly(ethyleneimine)	Electrospinn. Dipping	Thin film polymorph	Q-TSMR	5	4	E 3	E 0.3	E 100/-	SO_2_, H_2_S	UC (linear)	Ammonia detection	LDL=12% TLV	2016	[537]
VOCs DMMP	3 – 5 000	Ti-Phthalocyanines	Spray coating	Tin film 22 kHz shift	Q-TSMR	10	14	- E 0.3	- 6.9	150 -	-	UC (linear) PCA	Analyte detection PCA biplots	Partition ratios No TLV data	2016	[538]
Formaldehyde		Poly(dopamine)	Drop coating	Thin film Nanotubes	Q-TSMR	10	5	E 233	0.1	8.2/2.2	H_2_S, CO_2_, H_2_, CH_4_, NH_3_	UC (linear) low concentrations	Formaldehyde detection	LDL=14% PEL LDL=375% TLV	2016	[314]
Hydrogen	100 – 2 000	Tin dioxide & Pd NPs	DC magnetron sputtering	Thin bilayer	SAW LiNbO_3_	100	4	E 58	E 100	E 5/600	-	UC (not linear)	Hydrogen detection	LDL<0.25% LFL 175 °C operation	2016	[539]
Formaldehyde	0.5 – 3.5	GO	Drop coating	Thin film	Q-TSMR	9	1	22.9	0.06	E 90/120	VOCs, large	UC (linear)	Formaldehyde detection	LDL=8% PEL LDL=625% TLV	2016	[478]
Humidity	> 10 000	GO & poly(ethyleneimine)	Spray coating	Thin films 4 - 6 kHz shift	Q-TSMR	10	6	0.001	-	7/5	-	UC (not linear)	Relative humidity evaluation	-	2016	[186]
Methane	500 – 50 000	Cryptophane A	Drop coating Spin coating	Thin film	Q-SAW	300	2	0.02	500	12/20	Humidity large	UC (linear)	Methane detection	LDL=1% LFL	2016	[482]
VOCs DMMP		Poly(3-hexyl thiophene)	Electro-chemical	Porous film	Q-TSMR	10	5	11.7	0.4	E 10/10	Humidity	UC (linear)	VOCs detection	No TLV data	2017	[540]
VOCs	100 -500	Polymers	Spray coating	Thin film 0.2 -2.4 μm	Si μ-Clv C μ-Clv	0.12 0.03	SA 7	1.6	7.5	E 6/6	-	PCA	VOCs detection PCA scores	Enough sensitivity	2017	[254]
Hg	0.024 – 0.365	Au mercury	Evaporation	Thin film 50 nm	Q-SAW	131	1	E 10 000	0.0014	-	Humidity, VOCs, NH_3_	UC (not linear)	Cross sensitivity	LDL=28% TLV	2017	[508]
Nitroaromatic compounds	-	Methylated mesoporous silica	Dip coating	Thin film	Q-TSMR	100 9	1	212 000 1 167	<0.001 < 0.075	-	Humidity	Absorption isotherms	Molecular simulation	LDL=10% TLV	2017	[437]
Amine Propylamine	25 - 250	Solvate IL	Drop coating	Thin films	Q-TSMR	9	6	2 000	0.0054	E 2000/-	Humidity Ethanethiol	UC (linear)	Amine detection	LDL=3%TWA	2017	[189]
VOCs	400 – 40 000	Zn substituted porphyrins on ZnO	Drop casting	Thin layers of coated ZnO NP	Q-TSMR	20	4	E 0.07	-	E 40/60	Humidity	UC (not linear) PCA	VOCs detection PCA biplots	Cell cultures discrimination	2017	[94]
Hydrogen		ZnO	Vapor-liquid-solid technique	Films 200 nm Nano wires	Q-SAW	69.5	4	0.015	2117	9/11	-	UC (linear)	Deuterium, protium detection	LDL=5.7% LFL	2017	[541]
VOCs	-	Short peptides and Au NPs	Drop casting	Thin films 20-50 kHz shift	Q-TSMR	20	SA 5	-	-	-	-	PCA biplots	Molecular docking simulations	-	2017	[57]
Hydrogen	2 000 – 10 000	Pd	Electron beam evaporation	Thin films 25; 50 nm	Si μ-Clv	Static bend	2	-	250	300/-	-	UC(not linear)	Hydrogen detection	LDL=0.6% LFL	2017	[498]
Nitrogen dioxide	0.4 – 16	ZnO	RF Magnetron sputtering	Thin film 100 nm	Q-SAW	99.5	1	E 3500	1	-	H_2_, CO, acetone	UC (not linear)	NO_2_ detection	LDL=200% AWG D also measured	2017	[542]
VOCs	500 -100 000	IL & polymers composite	Electrospray	Thin films 2 – 16 kHz shift	Q-TSMR	5	1 5 harmonics	-	-	-	-	PCA, QDA	Fundamental research	TSMR – D	2017	[237]
CO, NO_2_, NH_3_	10 – 10 000	GO, N- substituted pyrrole deriv., Au-NP	Spin coating	Thin films	Q-TSMR	10	3	-	-	-	-	PCA	Analytes clustering	2 PCs scors	2017	[251]
Sulfur dioxide	50 -1 250	Ring-substituted poly(aniline)s	Electro-polymerization	Thin films 1 - 2 μm	Q-TSMR	10	3	0.19	E 10	1800/E 1800	-	UC (linear) Low concentration	Sulphur dioxide detection	LDL=500% TLV	2017	[494]
Methanethiol	0.05 - 1	Natrium Manganese Oxide	Casting, spreading	Thin film, Nano sheets	Q-TSMR	10	1	48	E 0.1	-	Humidity, acetic acid	UC (not linear)	Methanethiol detection	LDL=20% AWG	2017	[543]
Hydrogen	2 000 – 20 000	Pd/ZnO Bilayer	PLD	Thin film 330nm	Q-SAW	70	3	0.51	59	12/-	-	UC (linear)	Hydrogen detection	LDL=5% LFL	2017	[497]
Formaldehyde	0.05 - 50	4,4’-diaminodiphenyl sulfone Cu complex	PVD & chemical reaction	Thin films	Q-TSMR	10	4	E 50	E 0.01	9/11	Humidity, ethanol, VOCs	Dynamic response Absorp. enthalpy	Formaldehyde detection	LDL=1.3% PEL LDL=62% TLV	2017	[120]
Formaldehyde	1 - 50	Diphenyl sulfone urea dry-gel	Drop coating	Hydrophobic thin film	Q-TSMR	10	2	E 5	E 0.5	9/8	Humidity, CO_2_, H_2_S, NO_2_	UC (linear)	Formaldehyde detection	LDL=67% PEL LDL=3 000% TLV	2017	[544]
Amine Trimethylam.	5 - 150	GO/chitosan	Drop coating spreading	Nano-composite	Q-TSMR	6	3	4.8	1.3	16/75	Toluene, Acetone	UC (not linear)	Amine detection	LDL=13% TLV	2017	[182]
DMMP	0.1 - 60	TiO_2_-SiO_2_ functionalized with fluoroalcohol	Drop coating	Mesoporous film	Q-TSMR	10	4	E 0.02	E 100	E 10/10	VOCs	UC (linear) below 1 ppmv DMMP	DMMP detection	No TLV data	2017	[118]
Beta-caryophyllene	10 – 1 000	Au NP, 2- mercapto-benzothiazole	Dipping Drop coating	Polymorph film	Q-TSMR	10	2	0.17	0.32	19/-	Limonene, Ocimene	UC (linear)	Beta-CF detection	Enough sensitivity	2018	[545]
CWA	0.17 – 2.91	Organic materials Nano clay	Casting	Composite film	Q-TSMR	10	6	-	0.17	60/-	Humidity	-	CWA detection	Enough sensitivity No TLV data	2018	[179]
VOCs BTEX	200- 2 000	Organic silicate	PE-CVD	Thin film 20 – 300 nm	Q-TSMR	10	2	0.12	19	60/60	Humidity	UC (not linear)	VOCs detection	LDL=20% TLV	2018	[312]
VOCs	30 - 1200	Chitosan	Dipping	Polymorph film	Q-TSMR	10	1	0.015	70	15 -	Humidity	UC (linear)	VOCs detection	LDL>300% TLV !?	2018	[546]
VOCs Ethanol	-	ZnO NP & peptide nano composite	Drop casting	50nm NP agglomerate	Q-TSMR	20	SA 4	-	-	-	-	PCA biplot	Food analysis	Only scaled values given	2018	[249]
Ammonia	1 - 35	SiO_2_, TiO_2_, SiO_2_-TiO_2_ Composite	Sol-gel spin coating	Tin films 200 nm	Q-SAW	200	3	E 18 k	E 1	80/60	Humidity, H_2_, CO, NO_2_, H_2_S	UC (linear)	Ammonia detection	LDL=4% TLV	2018	[547]
DMMP	5 - 50	In_2_O_3_	Casting, spreading	Hollow NP Au decorated	Q-TSMR	6	2	2.1	E 5	E 100/400	VOCs	UC (linear)	DMMP detection	No TLV data Wireless TSMR	2018	[487]
Trimethyl amine	0.6 – 4.8	GO/Cu_2_O nanocomposite	Self-assembly Layer by layer	Thin film 800 nm	Q-TSMR	8		8.9	0.23	20/E 35	Humidity, NH_3_, HCHO, C_2_H_5_OH	UC (linear)	Trimethyl amine detection	LDL=2.3% TLV	2018	[481]
VOCs Xylene	5 - 20	Polymer	Spray coating	initial shift <2MHz	Q-SAW	433	8	E 10 k	-	-	-	-	VOCs detection	Polar plots Preconcentration	2018	[548]
H_2_S, NH_3_, Dimethylamine		CNTs, Graphene, PANI, CuO	Dipping	Thin film Multilayered	Q-TSMR	9	SA 4	6.5	E <5	30/E 60	-	UC (linear), PCA, LDA, PLSR	Food quality eggs	Enough sensitivity for application	2018	[343]
VOCs Toluene	1 – 100	ITO NPs	Spin coating	Thin film mesoporous	Love-SAW	160	4	E 1	E 15	-	Humidity	-	VOCs detection	Fundamental acusto-electric investigation	2018	[549]
Ammonia	0.005 – 0.3	carboxyl group functionalized silica	Ink jet printing	Thin film Porous NPs	μ-Clv Si	0.1	1	E 67	0.005	110/-	Humidity large	UC (not linear)	Ammonia detection	LDL=0.02% TLV	2018	[289]
Aniline	1.4 – 10.8	MOF-5	Ink jet printing	Thin film Porous MOF	μ-Clv Si	0.084	1	E 0.27	1.4	E 110/300	Carbon dioxide, ammonia	UC (not linear)	Aniline detection	LDL=28% PEL LDL=140% TLV	2018	[174]
CO	0.01 – 2.6	Ni-MOF-74	Ink jet printing	Thin film Porous MOF	μ-Clv Si	0.084	4	E 217	0.01	E 120/120	Ammonia, carbon dioxide	UC (not linear)	CO detection	LDL=0.03% TLV	2018	[495]
Hydrogen	2 000 – 20 000	Pd/WO3 Bilayers (nanostructured)	PLD	Thin film 620nm	Q-SAW	69.5	8	0.13	E 2000	E 70/30	-	UC (not linear)	Hydrogen detection	LDL=5% LFL	2018	[550]
Safrole	0.07 - 3	Poly(acrylonitrile)	Electro-spinning	Thin film Nanofiber	Q-TSMR	10	4	0.7	E 0.03	500/-	Methanal, ammonia	UC (linear)	Safrole detection	No TLV data Good sensitivity	2018	[551]
Methane	> 10 000	Cryptophane-E	Dispensing	Thin film	Q-SAW	204	1	E 0.015	E 2000	50/72	Methane	UC (linear)	Methane detection	LDL=4% LFL	2018	[483]
Formaldehyde	1 - 40	PODS-covered PDA	Dipping	Thin films	Q-TSMR	10	4	E 9	E 0.5	-	Humidity	Dynamic responses	Formaldehyde detection	LDL=67% PEL LDL=3125% TLV	2018	[187]
Aromatic VOCs Toluene	20 - 800	Poly(n-Octadecylsilane)	-	Hydrophobic	Q-TSMR	10	4	E 0.2	20	10/9	Humidity, VOCs large	UC (linear)	VOCs detection	LDL=20% TLV	2018	[114]
Formaldehyde	0.1 - 30	Poly(dopamine) on SiO_2_	Drop coating	Thin film Mesoporous	Q-TSMR	10	4	53	0.1	5/3	Humidity, NH_3_, acetone	UC (linear)	Food quality Formaldehyde	LDL=14% PEL LDL=600% TLV	2018	[117]
Formaldehyde	1 - 100	CuO-ZnO	Electrospinning	Film 25 nm Nanofibers	Q-TSMR	5	5	E 0.66	E 0.5	E 60/-	Ethanol, acetone	UC (linear)	Formaldehyde detection	LDL=67% PEL LDL=3125% TLV	2019	[552]
Nitroaromatic compounds	0.005 – 0.2	Poly(pyrrole)-bromophenol blue	Electrochemical	Nanostructured	Q-TSMR	10	12	2 200	0.0005	5/10	Multiple VOCs	UC (linear)	Detection of analytes	LDL=1% TLV for Trinitrotoluene	2019	[488]
Nitrogen dioxide	0.5 -30	PbS		Colloidal quantum dots	Q-SAW	200	2	910	0.032	45/58	H_2_S, NH_3_, H_2_, C_2_H_5_OH	UC (linear)	NO_2_ detection	LDL=6.4% AWG	2019	[553]
VOCs	-	Haipin DNA (hpDNA) on Au NP	Drop coating	10kHz initial freq. shift	Q-TSMR	20	SA 7	-	-	-	-	PCA scores & loadings	VOCs detection	In silico DNA screening	2019	[58]
VOCs	> 3 000	Polymers	SAM	20 Bilayers	FBAR AlN / Si	2 450	VSA 1	130	40	E 20/60	-	UC (not linear) PCA, LDA	VOCs detection, discrimination	Temperature modulation	2019	[238]
Narcotics precursors	-	Triptycene-based affinity materials	Electro-spray	10 ng, 50 kHz Initial shift	HFF Q-TSMR	195	5	-	-	-	-	-	Relative affinity evaluation	Illegal substance detection	2019	[491]
Hexanal	1 -200	MIP (Methacrylate & silica composite)	Drop coating	-	Q-TSMR	8.98	1	3	1	E 45/110	Humidity, VOCs	UC (linear)	Hexanal detection	No TLV data	2019	[180]
Hydrogen	1 – 400	Pd	Electron beam	Thin film 20 nm	Si μ-Clv	0.0188	1	-	-	4 500	-	Dynamic response	Hydrogen detection	Too long RT 38 ppbv resolution	2019	[554]
H_2_, He, O_2_. CH_4_.CO_2_, CO	1 - 5	No coating Physical sensor	-	-	Si μ-Clv	0.023	1	-	-	60/-	-	Mass and viscous load	Analyte selective detection	Sensitive enough for LFL	2019	[439]
Hydrogen sulfide	0.5 – 50	CuO	Spin coating Sol-gel	Thin film 54 nm	Q-SAW	200	1	E 4000	E 0.5	600/1100	Humidity, NH_3_, Ethanol	UC (linear)	Hydrogen sulfide detection	LDL=10% AWG	2019	[496]
Oxygen	> 10 000	ZnO	PLD	Thin film granular	LGS-SAW	335	4	E 15	E 2 000	120/170	-	UC (not linear)	Oxygen detection	High temperature sensor 750°C	2019	[555]
Ammonia	0.06 – 400	La doped AlPO-5	Drop casting	Tin film	Q-TSMR	10	2	100	0.06	228/58	Humidity, CO_2_, Acetone, NO_2_	UC (not linear)	Ammonia detection	LDL=0.24% TLV	2019	[158]
VOCs Benzene	10 - 40	Chromatographic column	-	-	Porous Si n-Clv	10.45	4	E 8	1.9	-	-	UC (linear)	VOC detection	LDL=400% TLV	2019	[356]
Hydrogen	1 000 – 40 000	Pd/Cu	Electro-deposition	Nanowires	SAW LiNbO_3_	150	1	E 6.4	E 1000	4/4	Humidity, NH_3_, H_2_S	UC (linear)	Hydrogen detection	LDL=2.5% LFL	2019	[557]

AET: 2-aminoethanethiol

Beta-CF: Beta-caryophyllene

CNT: Carbon nanotubes

Clv: Cantilever

CWA: Chemical warfare agent

D: Dissipation factor

DCM: Dichloromethane

DMMP: Dimethyl methyl phosphonate

DNT: Dinitro toluene

GO: Graphene oxide

ITO: Indium tin oxide

LDL: Low limit of detection

LGS: Langasite, La_3_Ga_5_SiO_14_

MAPLE: Matrix-assisted pulsed laser evaporation

LIFT: Laser induced forward transfer

MBA: 4-mercaptobenzoic acid

MIP: Molecularly imprinted polymer

MNA 6-mercaptonicotinic acid

NP: Nano particles

PAH: Poly(allylamine hydrochloride)

Pc: Phthalocyanine

PDA: Poly(dopamine)

PDMS: Poly(dimethyl siloxane)

PEDOT:PSS/PVP: Poly(3,4-ethylenedioxythiophene): Poly(styrene sulfonate)/poly(vinylpyrrolidone)

PLD: Pulsed Laser Deposition

PODS: Poly(n-octadecyl siloxane)

PVD: Physical vapor deposition

PZT: PbZr_x_Ti_(1-x)_O_3_ (PbZr_0.52_Ti_0.48_O_3_ in this case)

RGO: Reduced graphene oxide

SW/MW: Single wall / multiple wall

TMA: Trimethylamine

TNT: Trinitrotoluene

## Footnotes

1 Experiments making use of Fourier transform infrared external reflectance spectra and functionalized surface acoustic wave devices.

2 The authors refer to cyclodextrins for chiral recognition, nickel camphorates for Lewis bases such as pyridine or organophosphonates, and phthalocyanines for aromatic compounds for which specific chemical interactions such as metal coordination, “cage” compound inclusion, or π-stacking were expected (chiral recognition in general will be addressed in a later subsection of this review).

3 The term “substrate” is used for enzymatic reactions. For sensing processes, the appropriate concept is “analyte”.

4 In the case of insects and arthropods, the OBPs are secreted in the sensillum lymph, having accessibility to a restricted number of odorant receptors inside a sensillum only, but having greater diversity [25, 28–30].

5 Humans possess ~350 “olfactory” genes but are able to distinguish more than 100,000 smells [37].

6 LDL and all other parameter describing the performance of the sensors will be addressed at the beginning of the section “Targeted analytes” below)

7 The given value of the concentration was inferred by the authors of the present review from the saturation vapor pressure provided by [53].

8 Here, metal-free does not mean a phthalocyanine without any central atom, but a H_2_ phthalocyanine.

9 Data evaluation methods addressed are multivariate data analysis and artificial intelligence procedures.

10 The ATR-FTIR spectra were recorded for the synthesized Pcs alone and together with ad/absorbed analyte. The difference between the two above-addressed spectra (difference spectra) and the known analyte spectra enabled the identification of the vibrations specific to the absorbent–absorbate complexes.

11 The results will be addressed in the section “Targeted analytes” below.

12 DDS stands for 4,4-diaminodiphenyl sulfone, and MeOH for methanol. The compound was already addressed in the first part of the review in the section “Specific interactions and their role in receptor sensitivity and selectivity”.

13 This class of materials could also be addressed in the next section, “Functionalization of specially devised receptor materials”, due to some kind of regular free spaces inside. However, it has been included in the present paragraph because its members are one-element materials whose intrinsic regularity comes from the nature of the chemical bonding and from the symmetry it induces.

14 Both values (detection limit and cross-sensitivity) inferred by the authors of the present review from the original data of the study [136].

15 The gas sensing is not the main target application of such materials, which are mostly used as molecular sieves [142] or for gas storage, chemical compound separation and heterogeneous catalysis [143–152].

16 The structure and names of some types of MOFs can be found in references [159, 173].

17 MOF-5 is addressed, for example, by the references [143, 159].

18 Table S3 in the supporting information of reference [176] shows that the water vapor concentration in the cross-sensitivity test was 1400 ppm while the CEES concentration was 22.6 ppm. Extrapolating the water sensitivity to 50% RH at 40 °C (~20,000 ppm) will result in a response twice as large for background humidity as for CEES.

19 This type of transduction was addressed in the first part of this review in the section “MSTs combining mass sensitivity with other types of sensing”

20 Better in terms of adhesion, acoustic impedance matching, chemical compatibility and transducer protection against corrosive gases/vapor.

21 The chiral discrimination factor is the ratio of the partition ratios of the enantiomers.

22 This evaluation is possible by twice applying the equation 18 from the first part of the review.

23 This value was obtained with the authors’ definition, which consider the ratio of the sensor signals.

24 Grand canonical Monte Carlo simulations performed using the RASPA software package [233].

25 The optimization uses Kullback-Leibler divergence evaluations [235].

26 This topic exceeds the frame of the present review and is kept at a minimal extent. The formal terminology and the mathematical rigor were not the main priority of this section. Complementary information about the considered topics can be found in the addressed references.

27 The imprecise term “variability” is used here, because the “variance” of the data has not been addressed here.

28 In the design matrix, as in the data matrix, the rows express events (design events in this case), but the columns, in contrast, contain factor values (or products of factor values for the second-order terms) as they appear in the polynomial approximation given by Eq. (1).

29 This term might also contain the errors.

30 The article was cited exclusively for multivariate data analysis purposes. The authors delimit themselves from the legally and morally inadequate content of the article and publishing journal.

31 The algebraic trick of multiplication with the transpose matrix in the following equation is required because ***X*** is generally not a square matrix and therefore is not invertible.

32 The PLSR variant presented here is known as PLSR II and allows one to tackle all factors ***C*** at once. An older version, PLSR I, introduces the factors one by one as column vectors ***c***_***α***_ (one element for each event).

33 The term “perceptron” is used either to define a certain type of AN, or to address a class of simple ANNs containing perceptron-ANs in its nodes.

34 In the classification methods, the feature space of the data set becomes an input space, while the output space is categorical. For SVM, the term “feature space” is therefore employed for a higher-dimensional space obtained by the mapping of the input space (see later in the text). Thus, the SVM feature space is in fact a space of features of features.
